# Development of Fluorescent 4-[4-(3*H*-Spiro[isobenzofuran-1,4′-piperidin]-1′-yl)butyl]indolyl Derivatives as High-Affinity Probes to Enable the Study of σ Receptors via Fluorescence-Based Techniques

**DOI:** 10.1021/acs.jmedchem.2c01227

**Published:** 2023-03-15

**Authors:** Francesca Serena Abatematteo, Maria Majellaro, Bianca Montsch, Rubén Prieto-Díaz, Mauro Niso, Marialessandra Contino, Angela Stefanachi, Chiara Riganti, Giuseppe Felice Mangiatordi, Pietro Delre, Petra Heffeter, Eddy Sotelo, Carmen Abate

**Affiliations:** † Dipartimento di Farmacia-Scienze del Farmaco, Via Orabona, 4, 79125 Bari, Italy; ‡ Centro Singular Investigación Quimica Biologica e Materiales Moleculares (CIQUS), Departamento de Quimica Orgánica, Facultade de Farmacia, Universidade de Santiago de Compostela, 15782 Santiago de Compostela, Spain; § Center for Cancer Research and Comprehensive Cancer Center, Medical University of Vienna, Borschkegasse 8a, 1090 Vienna, Austria; ∥ Department of Oncology, University of Torino, via Santena 5/bis, 10126 Torino, Italy; ⊥ Consiglio Nazionale delle Ricerche (CNR), Istituto di Cristallografia, Via Amendola, 70126 Bari, Italy

## Abstract

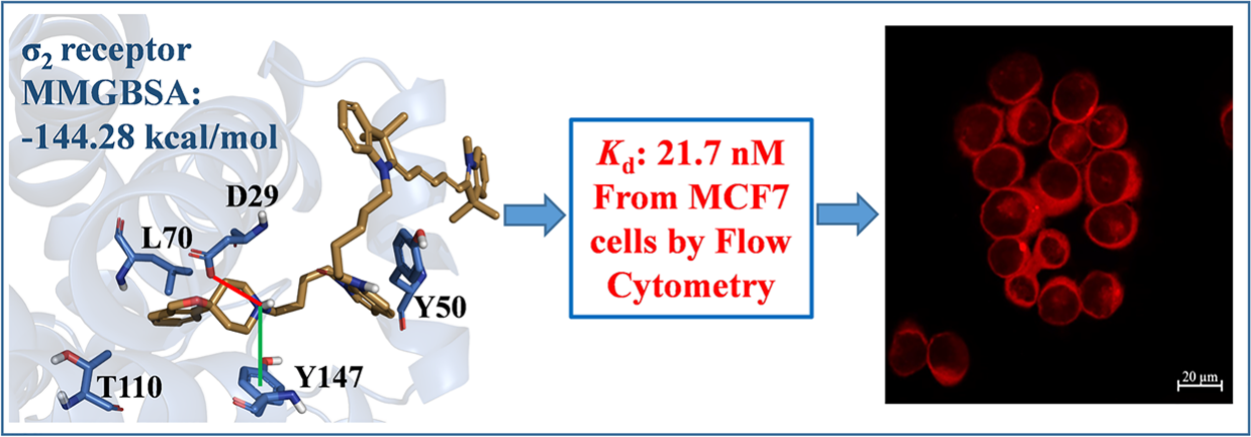

Sigma (σ) receptor subtypes, σ_1_ and σ_2_, are targets of wide pharmaceutical interest. The σ_2_ receptor holds promise for the development of diagnostics and therapeutics against cancer and Alzheimer’s disease. Nevertheless, little is known about the mechanisms activated by the σ_2_ receptor. To contribute to the exploitation of its therapeutic potential, we developed novel specific fluorescent ligands. Indole derivatives bearing the *N-*butyl-3*H*-spiro[isobenzofuran-1,4′-piperidine] portion were functionalized with fluorescent tags. Nanomolar-affinity fluorescent σ ligands, spanning from green to red to near-infrared emission, were obtained. Compounds **19** (σ pan affinity) and **29** (σ_2_ selective), which displayed the best compromise between pharmacodynamic and photophysical properties, were investigated in flow cytometry, confocal, and live cell microscopy, demonstrating their specificity for the σ_2_ receptor. To the best of our knowledge, these are the first red-emitting fluorescent σ_2_ ligands, validated as powerful tools for the study of σ_2_ receptors via fluorescence-based techniques.

## Introduction

Research interest in σ receptors has waxed and waned since their discovery in 1976 when they were identified as a subtype of opioid receptors (σ-opiate).^1^ Subsequent studies effectively divorced the σ proteins from the opioid receptors, and in the early 1990s, two protein subtypes were identified, σ_1_ and σ_2_, on the basis of the different pharmacology and tissue distribution.^2^ The σ_1_ subtype was cloned from different sources, including humans, in 1996, and only in 2016 was its crystal structure disclosed,^3,4^ revealing an unexpected fold and an unusual binding site. Crystallized as a trimer, each protomer includes a single transmembrane domain and a β-barrel flanked by α helices. A subsequent study has suggested how the ligands may enter the occluded binding site, determining conformational changes that elicit agonist versus antagonist activity upon binding. Several pieces of evidence show that agonists bias the receptor toward monomeric or lower-molecular weight oligomeric states, compared to antagonists that shift it toward high-molecular weight species.^5^ This subtype has been defined as a pluripotent chaperone that acts through protein–protein interactions. Several proteins have been identified as σ_1_ receptor client proteins, justifying the number of pharmacological actions elicited by σ_1_ ligands, that embrace antiamnesic, antidepressive, analgesic, and anticancer activity, among others.^5−7^ Recently, the σ_1_ receptor has been identified as a host protein for SARS-CoV-2 viral replication, and its ligands were able to exert antiviral activity.^8−10^ Although confounding factors in the *in vitro* assays were then revealed, the σ_1_ receptor represents a promising target for the development of anti-SARS-CoV-2 agents.^11−14^ Importantly, a mutation in the protein has been found in diseases such as amyotrophic lateral sclerosis (ALS)^15^ and distal hereditary motor neuropathy,^16^ shedding light on novel potential treatments for the disease. σ_1_ receptor ligands are under evaluation in clinical trials for the treatment of Alzheimer’s disease (AD),^17^ Huntington’s disease (HD),^18−20^ and neuropathic pain.^21,22^ As recently reviewed, some of these ligands have a convenient polypharmacological profile, with their action due to the interaction with more than one target whose modulation is beneficial for treating the disease.^23^ After some controversy about its identification,^24−27^ the lesser known σ_2_ subtype was finally identified as the TMEM97 protein in 2017, and its crystal structure was resolved in 2021.^28,29^ The receptor crystallizes as a homodimer, with each protomer built from four transmembrane helices and the binding site near the center of the protein. The determined identity and structure of the σ_2_ receptor will help to define the still ambiguous functional activity and develop a consensus protocol for defining the agonist or antagonist behavior of its ligands that is missing. Despite a completely different fold, the two subtypes share a convergent binding site, with functionally similar amino acids occupying similar space. This feature justifies the number of dual σ_1_ and σ_2_ receptor ligands that have been produced over the years.^29^ However, important information for the development of more selective σ_2_ receptor ligands was provided, and unexplored pharmacological functions, such as pain management, were also revealed. Despite its late identification, the σ_2_ receptor has attracted substantial interest, mainly because of its overexpression in a wide variety of tumors, and due to the cytotoxic action exerted by its ligands that hold promise as anticancer agents and as cancer diagnostics.^7,30−38^ Its high level of expression in the central nervous system (CNS) has also prompted research in the CNS disease field. Accordingly, the σ_2_ subtype has emerged for the treatment of AD as compounds from Cognition Therapeutics have been demonstrated to inhibit the binding of the Aβ 1–42 oligomer to neurons *in vitro* and to stop the subsequent neurotoxic cascade.^39−43^ Among these compounds, CT1812, named Elayta, emerged as the most promising. Defined as an allosteric antagonist of the σ_2_ receptor, Elayta has undergone several clinical studies and is now in the clinical phase for the treatment of mild to moderate AD.^44^

All of these features demand a better understanding of both poorly known proteins to fully exploit their therapeutic potential as targets of wide pharmaceutical interest. Specific fluorescent ligands may represent important tools for the investigation and characterization of these receptors in different biological contexts, such as cancer or neurodegeneration.

**Figure 1:**
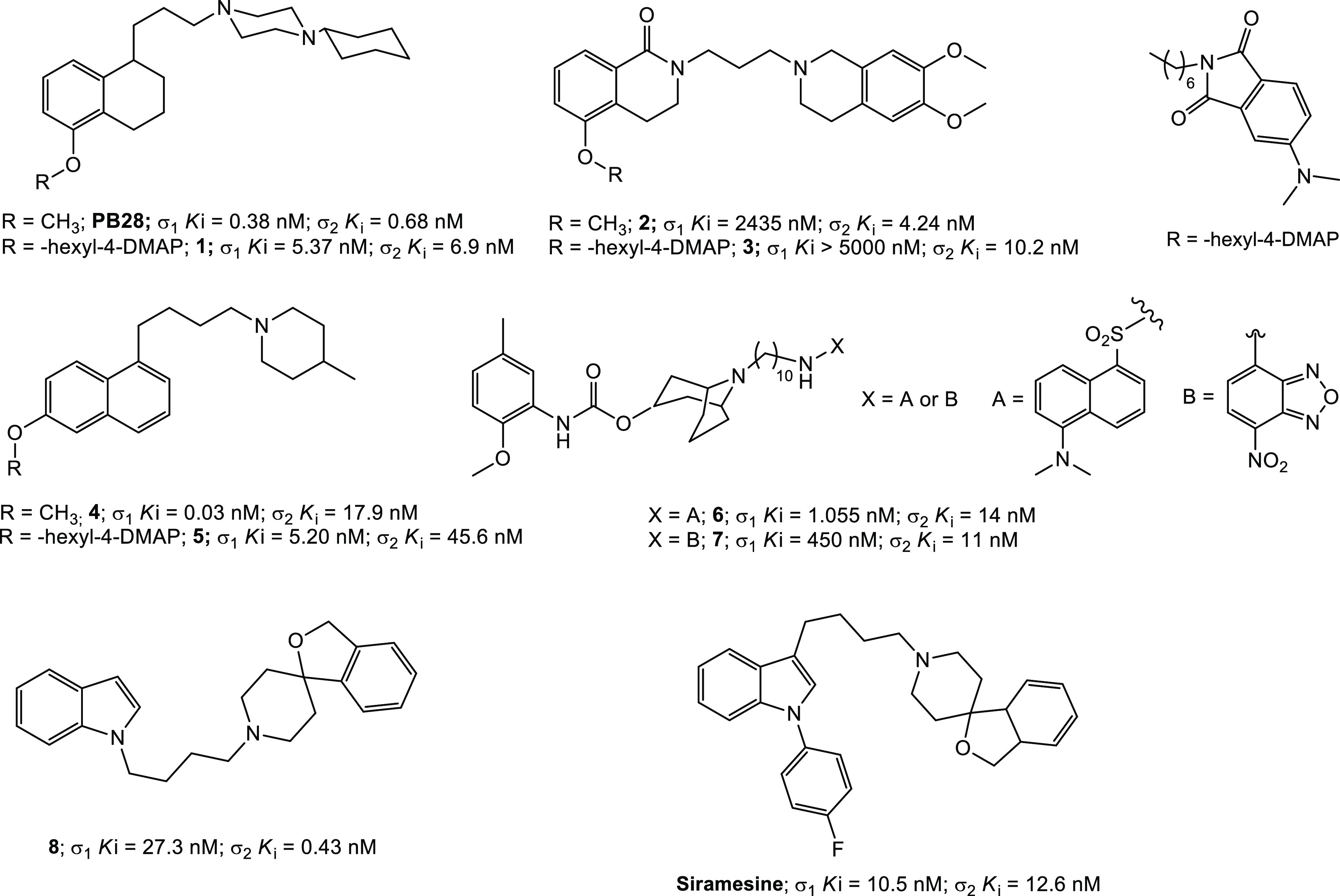
σ_2_ receptor reference compounds and fluorescent ligands.

With the aim of producing fluorescent σ_2_ ligands, in 2007 we started our investigation from structural modifications of the reference ligand PB28 (Figure 1).^45^ To keep the pharmacodynamic properties of PB28 unchanged, in the first attempts we only slightly modified its structure, by replacing the methoxytetralin moiety with a β-hydroxynaphthyl one.^46^ Despite the high affinity of some ligands for the σ_2_ receptor, the fluorescent properties were not appropriate for fluorescence studies in cell cultures. Thus, we identified the appropriate position on PB28 for conjugating different green-emitting fluorophores, through linkers bearing fluorescent tags at the ω position.^47,48^ A hexamethylene linker appeared to be the best compromise in terms of pharmacodynamic properties in particular when the fluorescent tag was the 4-(dimethylamino)phthalimmide (4-DMAP),^49^ as in compound **1** (Figure 1), having less impact on the pharmacodynamic properties of the parent compound PB28.^48^ Accordingly, this same decoration (i.e., the hexamethylene linker bearing a 4-DMAP at the ω position) was then used to obtain fluorescent ligands based on structurally different lead compounds targeting different receptors. In particular, the σ_2_ selective ligand **2** (Figure 1)^50^ was modified to obtain the highly σ_2_ receptor selective fluorescent ligand **3** (Figure 1).^51^ Similarly, the σ_1_ selective piperidine derivative **4**, known as PB212 (Figure 1),^52^ was modified to obtain the σ_1_ selective fluorescent ligand **5** (Figure 1).^53^ All of these ligands were successfully used in flow cytometry to detect the presence of the bound proteins and to perform binding assays avoiding the use of radioligands. Fluorescent ligands **1** and **3** were also used in confocal microscopy studies to validate σ_2_-targeting quantum dots (QDs) with superior fluorescent properties and to support the distinction of the σ_2_ receptor from the PGRMC1 protein complex.^26,54^ Interestingly, compound **3** showed promising cytotoxic properties against triple-negative breast cancer cells by engaging the σ_2_ receptor and deserves further investigation in this context.^34^

Fluorescent ligands based on a different σ_2_ receptor selective reference compound and diverse green-emitting fluorescent tags were also developed by another group, with valuable σ_2_ receptor affinity and selectivity [**6** and **7** (Figure 1)].^55,56^ However, all of these ligands are characterized by limited quantum yields and brightness, which may hamper the visualization of the receptors when poorly expressed. Additionally, the need for excitation with a 405 nm laser, which is not common in confocal microscopes or flow cytometers, could represent a limitation to visualizing the compounds bearing a 4-DMAP moiety.

Therefore, with the aim of widening the availability and applicability of fluorescent σ_2_ receptor ligands, we investigated scaffolds other than PB28 or **2**. Thus, next to the green-emitting small tag 4-DMAP, we inserted more powerful fluorescent tags emitting in the red and near-infrared (NIR) range of the light spectrum. In particular, derivatives of the σ reference compound siramesine (Figure 1 and Table 1)^57−60^ were generated, by functionalizing either the N-1 or C-6 position of the indole ring with fluorescent dyes as suggested by previous structure affinity relationship (SAfiR) studies.^33,57,61^ The *N-*butyl-3*H*-spiro[isobenzofuran-1,4′-piperidine] portion was either kept at the C-3 indole position as in siramesine or moved to the N-1 position. This latter change was made in agreement with previous studies that show how the shift of the butyl-spiropiperidine portion from the C-3 indole position to the N-1 position leads to a subnanomolar affinity and moderate σ_2_ receptor selectivity [compound **8** (Figure 1)].^33,61^ The selected fluorescent tags would enable more confident confocal microscopy and live cell microscopy studies, extending their use as imaging tools *in vivo*, as well.

**Table 1:**
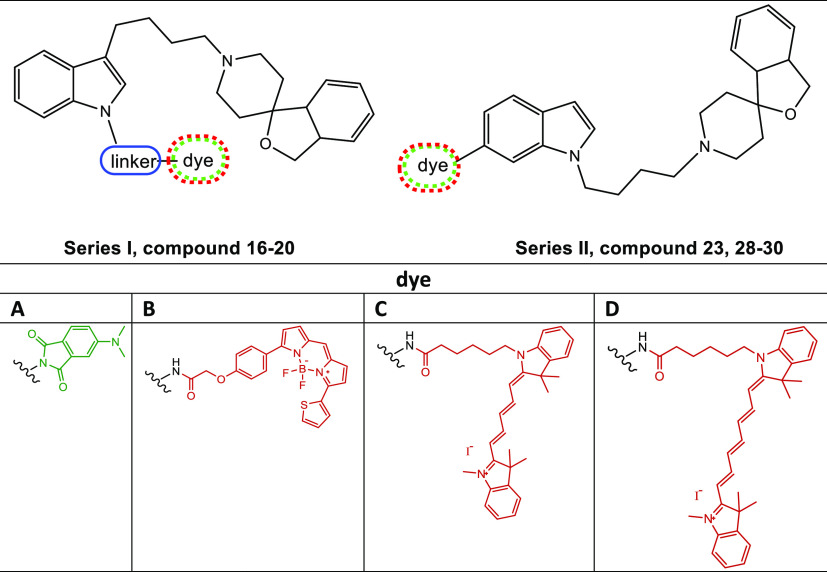
σ Receptor Affinities of Final Fluorescent Ligands and Reference Compounds and Their Photophysical Properties

			*K*_i_ ± SEM (nM)^a^		photophysical properties (CHCl_3_)		
	linker	dye	σ_1_	σ_2_	λ_ex_ (nm)	λ_em_ (nm)	quantum yield (%)
**16**	(CH_2_)_3_	A	39.3 ± 9.0	10.1 ± 1.1	395	490	6.85
**17**	(CH_2_)_6_	A	38.1 ± 5.2	3.84 ± 0.8	395	490	6.80
**18**	(CH_2_)_6_	B	473 ± 30	220 ± 12	587^b^	630^b^	ND^c^
**19**	(CH_2_)_6_	C	51.3 ± 3.2	30.2 ± 3.5	657	678	52.86
**20**	(CH_2_)_6_	D	88.8 ± 15.1	39.8 ± 4.2	762	787	39.08
**23**	–	A	296 ± 71	5.07 ± 1.07	395	490	1.31
**28**	–	B	>5000	–	587^b^	630^b^	ND^c^
**29**	–	C	448 ± 80	51.1 ± 5.1	657	678	47.83
**30**	–	D	569 ± 85	39.4 ± 6.1	762	787	40.24
siramesine	–	–	10.5	12.6			
**8**	–	–	27.3	0.43			
DTG	–	–		19.5 ± 1.5			
(+)-PTZ	–	–	3.10 ± 0.4				

a Values represent the mean of at least two separate experiments in duplicate ± SEM.

b Compounds dissolved in CH_3_OH for measurements of λ_ex_ and λ_em_.

c Not determined.

## Results and Discussion

### Chemistry

**Scheme 1:**
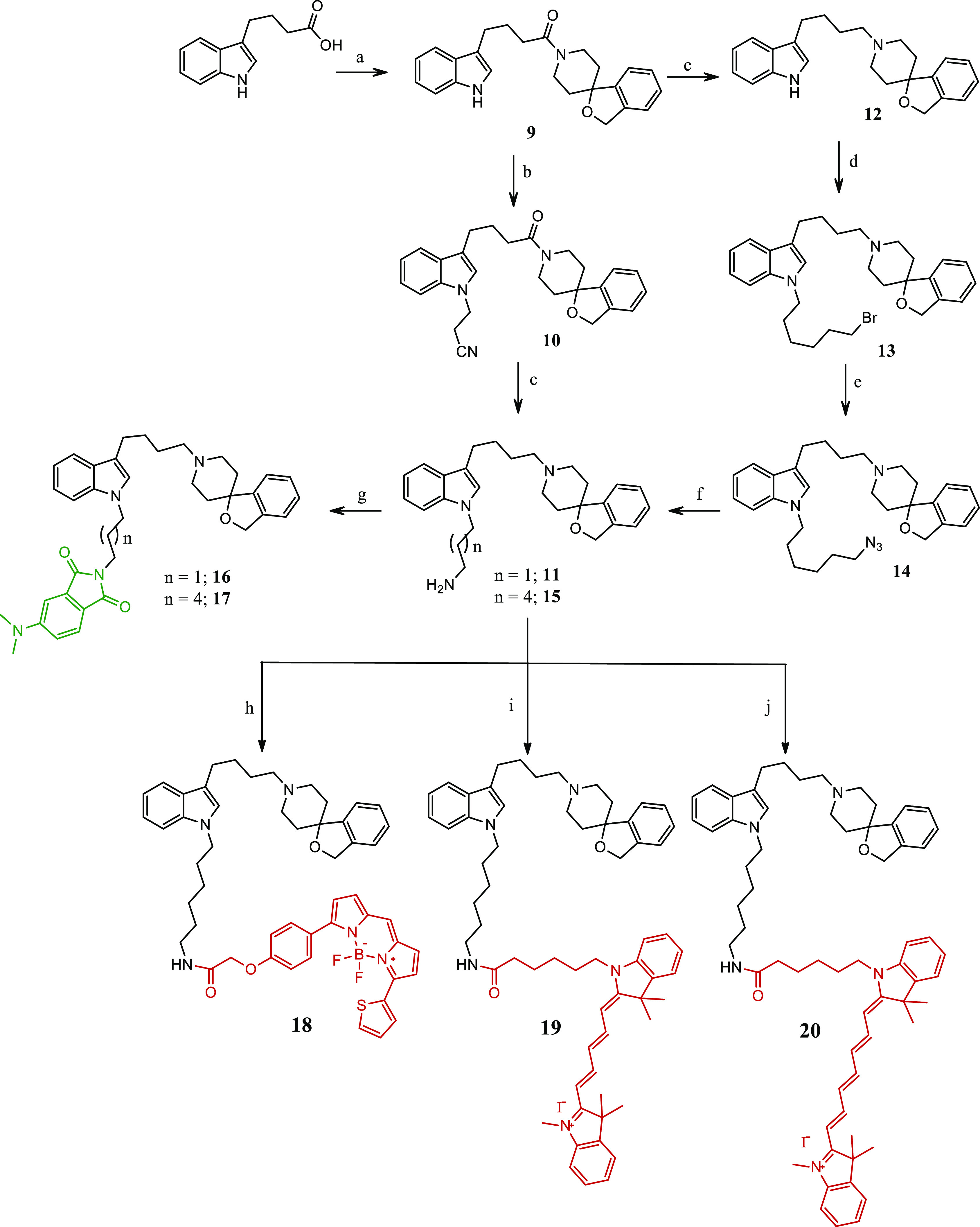
Synthetic Pathways for the Synthesis of Final Compounds **16**–**20** Reagents: (a) 3*H*-spiro[isobenzofuran-1,4′-piperidine], CDI, dry THF, RT, overnight; (b) 3-bromopropanenitrile, KOH, K_2_CO_3_, CH_3_CN, MW, 150 °C, 1 h; (c) BH_3_·DMS, dry MeOH, reflux, 4 h; (d) 1,6-dibromohexane, TBAB, KOH, dry DMF, RT, 2 h; (e) NaN_3_, dry DMF, 60 °C, 20 h; (f) PPh_3_, dry MeOH, 80 °C, 1 h; (g) 4-(dimethylamino)phthalic acid, CDI, dry DMF, RT, overnight; (h) BODIPY-TR-COOH, HATU, DIPEA, CH_2_Cl_2_, 30 °C, O/N; (i) Cy-5-COOH, HATU, DIPEA, CH_2_Cl_2_, 30 °C, overnight; (j) Cy-7-COOH, HATU, DIPEA, CH_2_Cl_2_, 30 °C, overnight.

**Scheme 2:**
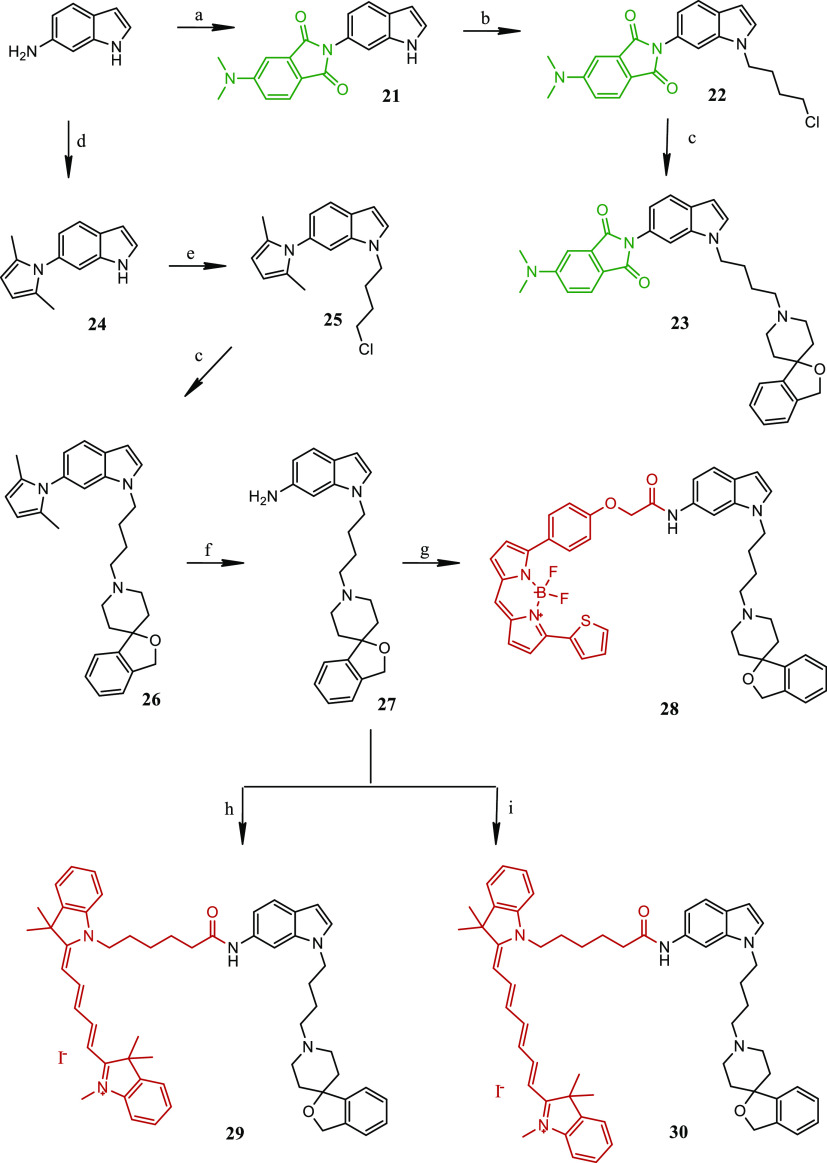
Synthetic Pathways for the Synthesis of Final Compounds **23** and **28**–**30** Reagents: (a) 4-(dimethylamino)phthalic acid, CDI dry DMF, RT, overnight; (b) 1-bromo-4-chloro-butane, *t*-BuOK, dry DMF, RT, 1 h; (c) 3*H*-spiro[isobenzofuran-1,4′-piperidine], K_2_CO_3_, CH_3_CN, reflux, overnight; (d) 2,5-hexanedione, dry toluene, reflux, 6 h; (e) 1-bromo-4-chloro-butane, TBAB, KOH, dry DMF, RT, 2 h; (f) NH_2_OH·HCl, 2:1 EtOH/H_2_O, 30 °C, overnight; (g) BODIPY-TR-COOH, HATU, DIPEA, CH_2_Cl_2_, 30 °C, overnight; (h) Cy-5-COOH HATU, DIPEA, CH_2_Cl_2_, 30 °C, overnight; (i) Cy-7-COOH, HATU, DIPEA, CH_2_Cl_2_, 30 °C, overnight.

The synthesis of novel fluorescent compounds **16**–**20**, **23**, and **28**–**30** is reported in Schemes 1 and 2. Key intermediate amide **9** was obtained by reaction between 3*H*-spiro[isobenzofuran-1,4′-piperidine]^57^ and the commercially available 4-(1*H*-indol-3-yl)butanoic acid, upon activation of the latter with 1,1′-carbonyldiimidazole (CDI) (Scheme 1). The indole nitrogen in **9** was alkylated with 3-Br-proprionitrile to provide intermediate **10**, whose nitrile and amide functions were reduced in one step with BH_3_·DMS to afford amine derivative **11**.

The hexamethylene homologue was obtained by previous reduction of the key amide **9** to the already known ammine **12**(57) with BH_3_·DMS and subsequent alkylation of the indole N atom with 1,6-dibromohexane. Hexyl bromide **13** underwent nucleophile substitution with NaN_3_ to afford intermediate azide **14**, which provided hexylamine derivative **15** upon reduction with PPh_3_, through the Staudinger reaction. Activation of the already known 4-(dimethylamino)phthalic acid^49^ with CDI followed by addition of amine **11** or **15** afforded the propyl- or hexyl-bearing fluorescent imide **16** or **17**, respectively, according to a previously used procedure^48^ (Scheme 1). Upon activation with 1-[bis(dimethylamino)methylene]-1*H*-1,2,3-triazolo[4,5-*b*]pyridinium-3-oxide hexafluorophosphate (HATU) in the presence of *N*,*N*-diisopropylethylamine (DIPEA) of the carboxylic functions of the Bodipy-TR (BDP-TR) or Cyanine 5 (Cy-5) or Cyanine 7 (Cy-7) fluorophores, the key intermediate amine **15** was transformed into the corresponding fluorescent amides **18**–**20** (Scheme 1). The synthetic pathways leading to the functionalization of 6-amino-indole are reported in Scheme 2. Reaction between 1*H*-indol-6-amine and 4-(dimethylamino)phthalic acid, previously activated by CDI, led to phthalic imide **21**. Alkylation of the indole N atom with 1-bromo-4-chloro-butane in the presence of tetrabutylammonium bromide (TBAB) and KOH led to butyl chloride intermediate **22** that was used to alkylate 3*H*-spiro[isobenzofuran-1,4′-piperidine] and provided final compound **23**. (Scheme 2).

The synthetic pathway for the synthesis of the corresponding red-emitting fluorescent ligands **28**–**30** started with the protection of the amine group in C-6 on the 1*H*-indole with 2,5-hexanedione upon removal of water from the reaction mixture, to afford pyrrole derivative **24**. As for final compound **23**, alkylation of **24** with 1-bromo-4-chloro-butane in the presence of TBAB and KOH led to butyl chloride intermediate **25** that was used to alkylate 3*H*-spiro[isobenzofuran-1,4′-piperidine] to achieve intermediate compound **26**. Treatment of this last compound with hydroxylamine hydrochloride under MW conditions provided key amine **27**, which underwent acylation through a previously described method to afford final fluorescent compounds **28**–**30** (Scheme 2).

### σ_1_ and σ_2_ Receptor Binding Affinities

The results from the radioligand binding assays are expressed as inhibition constants (*K*_i_ values) in Table 1, while the corresponding radioligand competition binding curves are depicted in Figure S1. Insertion of the green-emitting 4-DMAP fluorophore (dye A) retains or slightly ameliorates the affinity for the σ_2_ receptor for siramesine-like ligands **16** and **17** (*K*_i_ values of 10.1 and 3.84 nM, respectively) compared to siramesine (*K*_i_ value of 12.6 nM). Only a slight reduction in the σ_1_ affinity has been recorded with these ligands (*K*_i_ values of 39.3 and 38.1 nM, respectively) compared to siramesine (*K*_i_ value of 10.5 nM), thus resulting in a moderate (3–10-fold) σ_2_ selectivity. While siramesine is reported as a selective σ_2_ ligand, in our hands, this ligand has consistently shown a lack of selectivity, in classical binding protocols.^60^ The functionalization of **8** with dye A at position 6 of the indole ring results in ligands with high affinity and selectivity for the σ_2_ receptor, i.e., **23** (*K*_i_ values of 5.07 nM at σ_2_ and 296 nM at σ_1_). In more detail, despite a 10-fold reduction in the affinity at both receptor subtypes, the 60-fold selectivity for σ_2_ over σ_1_ of parent compound **8** is retained. The insertion of the BDP-TR fluorophore (dye B) on both of the indole structures leads to a dramatic decrease in the affinity at both receptor subtypes (*K*_i_ values ranging from 220 to >5000 nM) with the worst data displayed by compound **28**, an analogue of **8**. It is worth noticing that these BDP-TR-bearing compounds were less soluble than the other fluorescent ligands. Their 0.01 M concentration in the medium for biological assays was achieved only upon sonication. On the contrary, the red- and NIR-emitting cyanine-based fluorophores [dyes C (Cy-5) and D (Cy-7) (Table 1)] confer appreciable σ_2_ receptor affinities, in both the indole series functionalized with the fluorescent dye at the indole N-1 or C-6 position. While siramesine-like compounds **19** (*K*_i_ values of 30.2 nM at σ_2_ and 51.3 nM at σ_1_) and **20** (*K*_i_ values of 39.8 nM at σ_2_ and 88.8 nM at σ_1_) bind almost equally well both σ receptor subtypes, the **8**-like counterparts **29** (*K*_i_ values of 51.1 nM at σ_2_ and 488 nM at σ_1_ receptors) and **30** (*K*_i_ values of 39.4 nM at σ_2_ and 569 nM at σ_1_ receptors) show an ∼10-fold σ_2_ receptor selectivity. Thus, analogues of **8** always retain a certain degree of σ_2_ receptor selectivity toward the σ_1_ subtype, whereas the siramesine analogues are unselective, matching the profile at σ receptors of compound **8** and siramesine. These results together strongly suggest that the butyl-spiropiperidine portion at indole C-3 position strongly interacts with both σ binding sites, whereas the same functionalization at position N-1 leads to a weaker interaction with the σ_1_ receptor, while keeping the binding with the σ_2_ subtype. To gain insights into this behavior, computational studies were performed.

### Computational Studies

**Figure 2:**
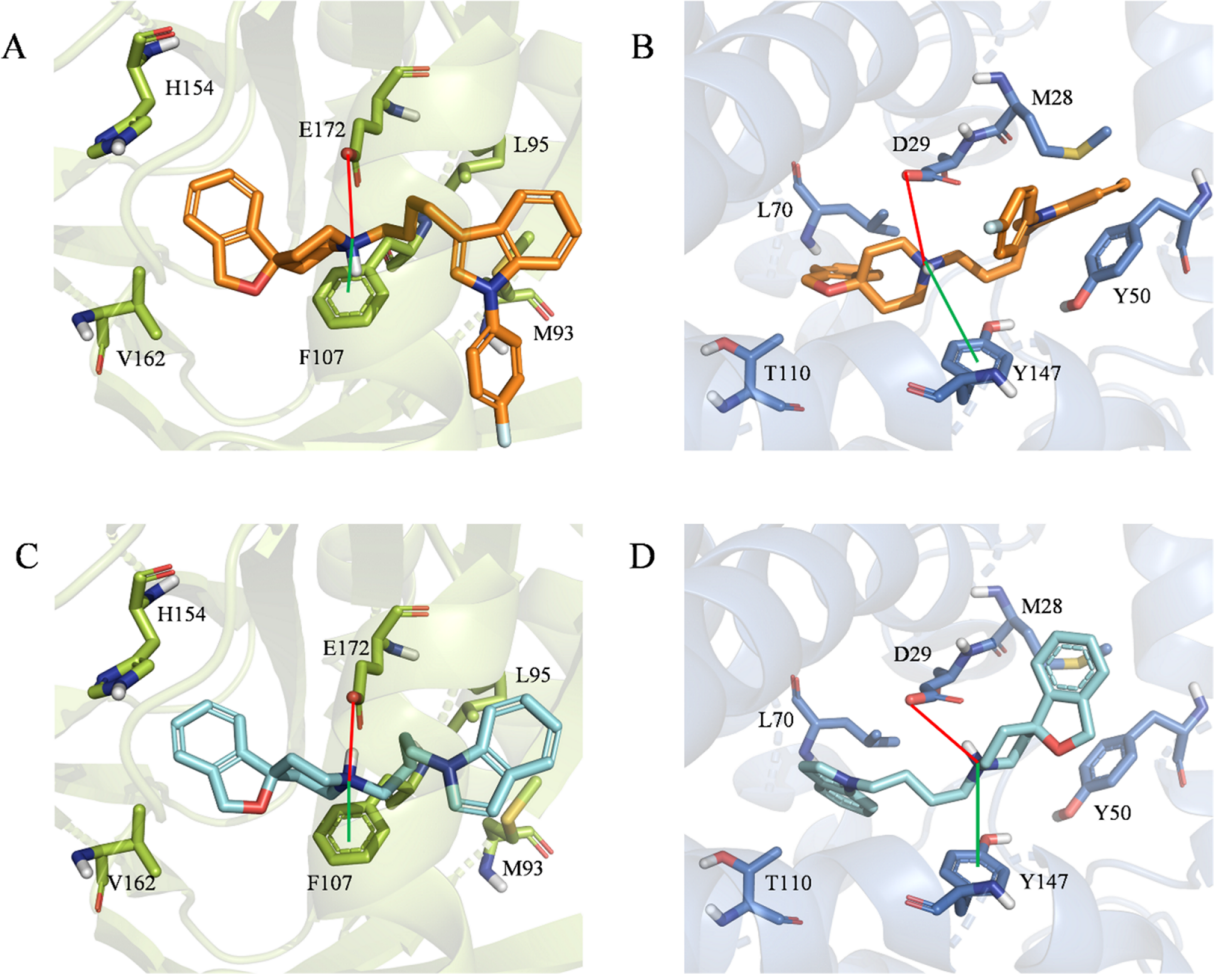
Top-scoring docking poses of (A) siramesine within the binding pocket of σ_1_ (PDB entry 6DK1), (B) siramesine within the binding pocket of σ_2_ (PDB entry 7M95), (C) **8** within the binding pocket of σ_1_ (PDB entry 6DK1), and (D) **8** within the binding pocket of σ_2_ (PDB entry 7M95). For the sake of clarity, only polar hydrogen atoms are shown. Important residues are rendered as sticks, while the proteins are represented as cartoon. Salt-bridge and cation−π interactions are depicted as red and green lines, respectively.

**Figure 3:**
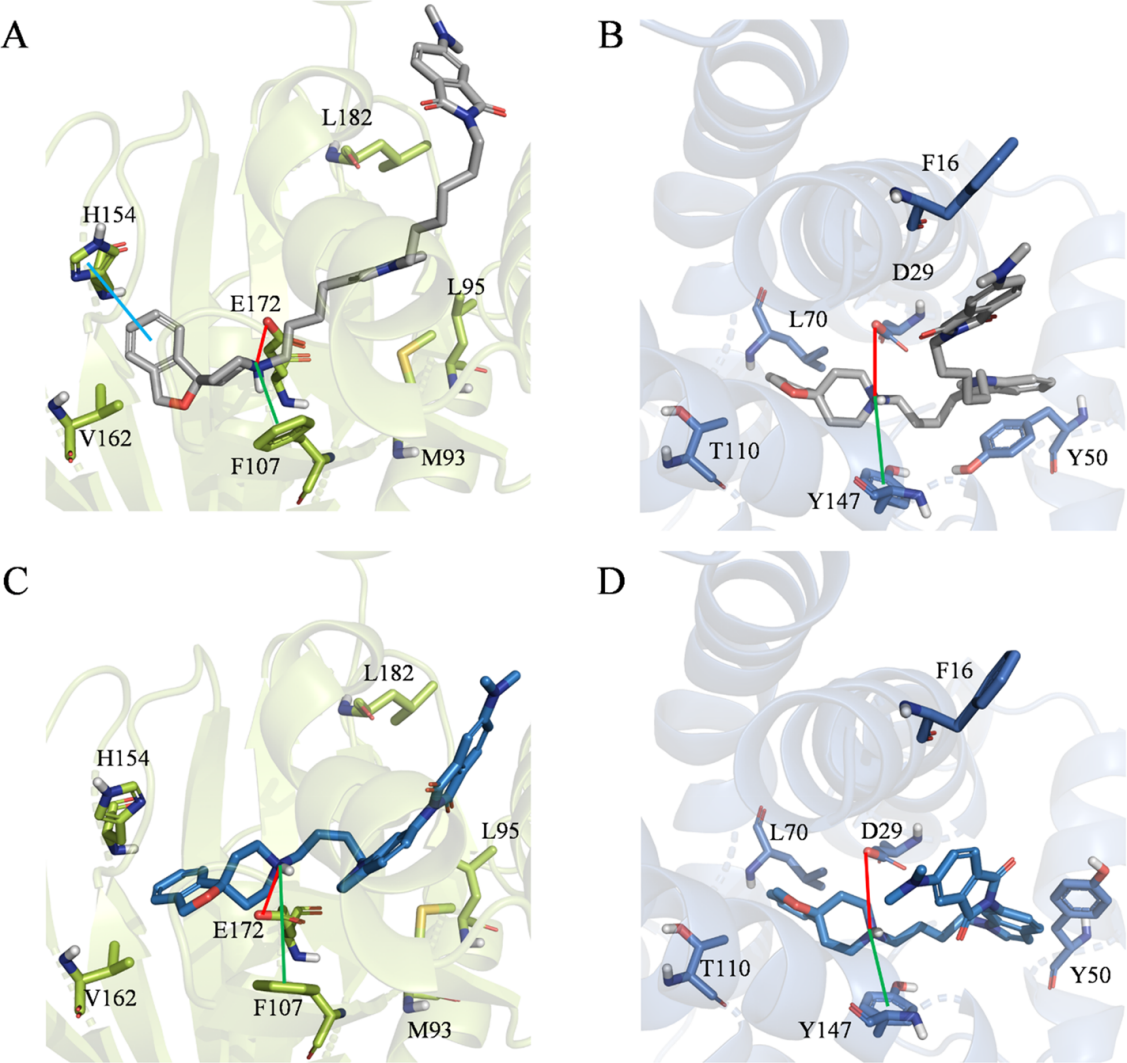
Top-scoring docking poses of (A) **17** within the binding pocket of σ_1_ (PDB entry 6DK1), (B) **17** within the binding pocket of σ_2_ (PDB entry 7M95), (C) **23** within the binding pocket of σ_1_ (PDB entry 6DK1), and (D) **23** within the binding pocket of σ_2_ (PDB entry 7M95). For the sake of clarity, only polar hydrogen atoms are shown. Important residues are rendered as sticks, while the proteins are represented as a cartoon. Salt-bridge and cation−π interactions are depicted as red and green lines, respectively.

To provide a molecular rationale behind the obtained experimental data, we performed molecular docking simulations within the binding sites of both σ receptors. It should be noted that, as far as σ_2_ is concerned, performing robust docking simulations was made possible only in 2021 by the group of Kruse et al., due to the release of the first X-ray structure of the bovine σ_2_ receptor at 2.4 Å resolution and in complex with Z1241145220.^62^ We initially focused our attention on two compounds belonging to our series, namely **17**, the derivative of our panel responsible for the highest σ_2_ affinity [*K*_i_ = 3.84 nM (Table 1)], and **23**, the ligand showing the highest σ_2_/σ_1_ selectivity [*K*_i_ of 5.07 nM vs 296 nM (Table 1)]. It is noteworthy that the decrease in the σ_1_ affinity returned by compound **23** seems to be substantially due to the presence of a butyl-spiropiperidine portion at position N-1 (**8**-like scaffold) of the indole ring (rather than at position C-3, siramesine-like scaffold) in full agreement with the activity data already published for these reference compounds [i.e., siramesine and **8** (Table 1)].^33,60^ Building on this evidence, we carried out preliminary docking simulations of both reference ligands on the σ receptors. Figure 2 shows the obtained top-scoring docking poses. Remarkably, the binding mode returned by the cognate ligands (based on the inspection of the employed crystal structures) is herein mostly confirmed. Molecular recognition is, in fact, the result of (i) an ionic interaction involving a positively charged nitrogen atom of the ligand and a negative charged residue, namely, E172 (σ_1_) and D29 (σ_2_), (ii) a cation−π interaction involving the same nitrogen atom and an aromatic residue, namely, F107 (σ_1_) and Y147 (σ_2_), and (iii) hydrophobic interactions with several residues of the pockets (M93, L95, and V162 in the case of σ_1_ and M28, Y50, and L70 in the case of σ_2_). Furthermore, in full agreement with the experimental data, siramesine returned similar MM-GBSA scores, when docked on σ_1_ (−129.77 kcal/mol) and σ_2_ (−127.48 kcal/mol). A substantial energy gap was instead observed upon comparison of the MM-GBSA scores of **8** in σ_1_ (−124.82 kcal/mol) and σ_2_ (−131.33 kcal/mol). Encouraged by these data, supporting the reliability of the predicted binding modes, the same docking protocol was applied to **17** and **23**. Figure 3 shows the obtained docking poses.

As expected, both investigated fluorescent ligands are predicted to efficiently bind the receptors by establishing the same interactions described for the reference compounds, namely, (i) an ionic interaction between the charged nitrogen atom and E172 (σ_1_) or D29 (σ_2_), (ii) a cation−π interaction with F107 (σ_1_) or Y147 (σ_2_), and (iii) several hydrophobic interactions with different residues. Even more interestingly, the computed binding free energies are again in agreement with the experimental data, thus further supporting the robustness of the performed docking simulations. In particular, **17** outperforms **23** in terms of the MM-GBSA score computed within the σ_1_ (−144.59 kcal/mol vs −135.35 kcal/mol) and σ_2_ (−165.82 kcal/mol vs −157.58 kcal/mol) binding pockets. On the basis of these results, we can here speculate that these differences might be related to the ability of **17** to establish hydrophobic interactions with L182 [σ_1_ (Figure 3A)] and F16 [σ_2_ (Figure 3B)]. These interactions in fact are not observed in the top-scoring docking posed returned by **23**.

**Figure 4:**
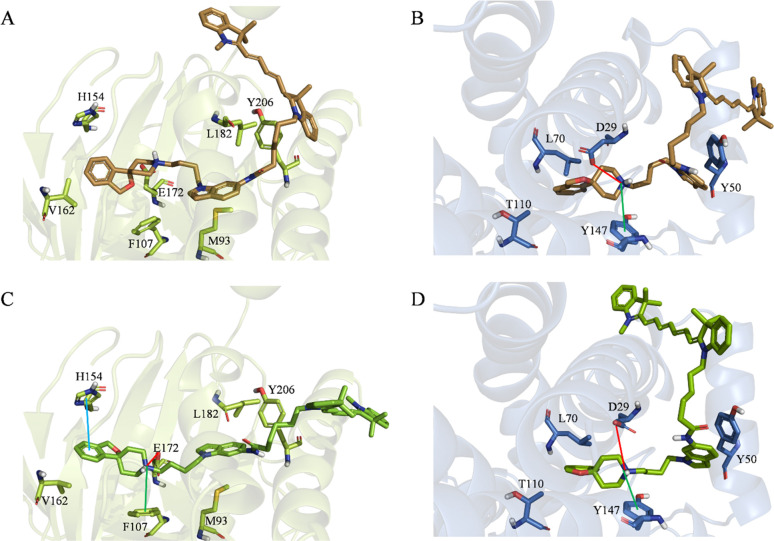
Top-scoring docking poses of (A) **29** within the binding pocket of σ_1_ (PDB entry 6DK1), (B) **29** within the binding pocket of σ_2_ (PDB entry 7M95), (C) **30** within the binding pocket of σ_1_ (PDB entry 6DK1), and (D) **30** within the binding pocket of σ_2_ (PDB entry 7M95). For the sake of clarity, only polar hydrogen atoms are shown. Important residues are rendered as sticks, while the proteins are represented as a cartoon. Salt-bridge and cation−π interactions are depicted as red and green lines, respectively.

Building on these encouraging results, we performed molecular docking simulations of fluorescence probes **29** and **30**. The aim was to provide a molecular rationale behind (i) the ability of these compounds to target both σ_1_ and σ_2_ receptors despite their very large size and (ii) their high σ_2_ selectivity (Table 1). As shown in Figure 4, the applied protocol returns reliable top-scoring docking poses (i.e., similar to those returned by reference compound **23**) for both ligands in both receptors, thus supporting the idea that the design strategy adopted for the herein presented series II (Table 1) is not endangered by a putative steric hindrance within the cavities. This is also indicated by the good MM-GBSA scores returned by the used protocol, being always better than −100 kcal/mol. In particular, and in full agreement with the experimentally observed σ_2_ selectivity, MM-GBSA scores equal to −125.04 kcal/mol (**29**–σ_1_), −108.53 kcal/mol (**30**–σ_1_), 144.28 kcal/mol (**29**–σ_2_), and −135.99 kcal/mol (**30**–σ_2_) were computed, thus putting forward the herein tuned computational protocol as valuable for a rational design of σ_2_ selective fluorescent probes.

## Fluorescence Studies

### Flow Cytometry Studies

**Figure 5:**
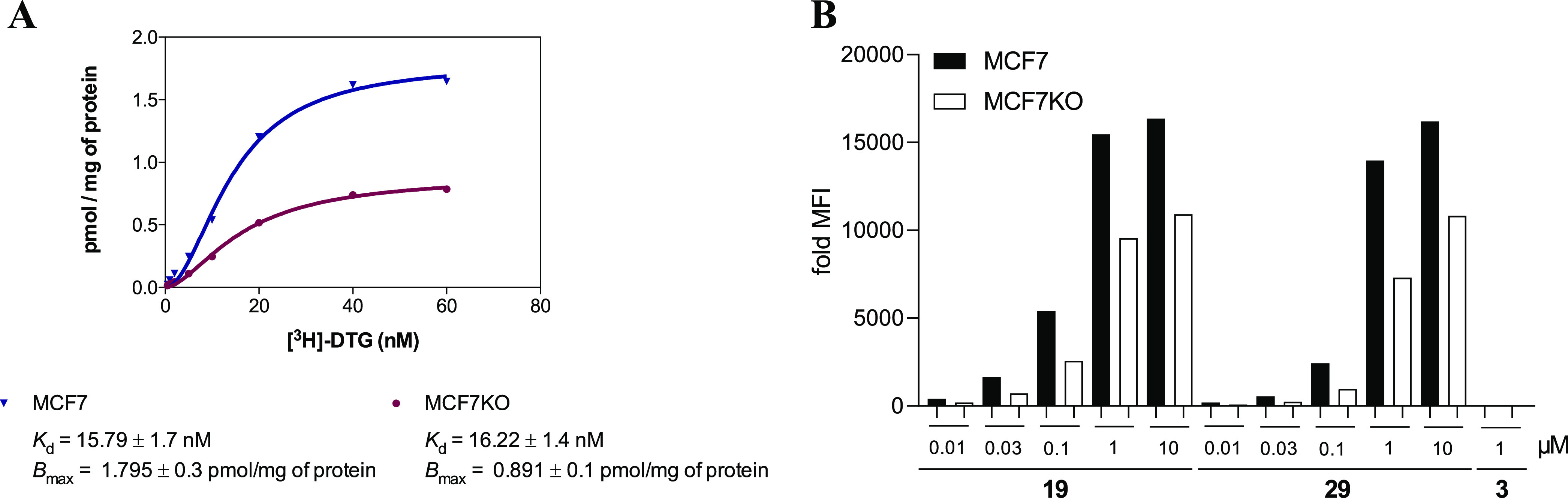
(A) Saturation binding assay on MCF7 and MCF7KO (silenced in TMEM97/σ_2_) cells. (B) Flow cytometry. Dose-dependent increase in the mean fluorescence intensity (MFI) in MCF7 and MCF7KO (one representative image of three repetitions) cells upon administration of compound **19**, **29**, or **3** at the indicated concentrations.

Cy-5-bearing ligands **19** and **29**, belonging to the two different series, were chosen for thorough characterization as σ receptor probes, because of their σ receptor binding profile and superior fluorescent properties [quantum yield (QY) values of 52.86% and 47.83%, respectively], compared to their 4-DMAP-bearing counterparts (QY values of 6.85% and 1.31% for compounds **17** and **23**, respectively). To gain insight into the biological behavior of the new ligands, flow cytometry studies were conducted in the breast adenocarcinoma cell line MCF7. This cell line was selected on the basis of its high σ_2_ receptor expression levels (*B*_max_ = 2.02 pmol/mg of protein), while the σ_1_ receptor was only marginally expressed (*B*_max_ = 0.17 pmol/mg of protein).^63^ Moreover, for comparison, a MCF7 clone (namely MCF7KO) was used, in which the σ_2_ receptor (TMEM97) was partially silenced by lentiviral transfection. The amount of residual σ_2_ receptor (*B*_max_ = 0.89 pmol/mg of protein) in the MCF7KO cells was measured by a saturation binding assay (Figure 5A) and further supported by flow cytometry studies performed with the two reference fluorescent ligands **1** and **3** (Figure S2). To mask the weakly expressed σ_1_ receptor, for the flow cytometry experiments, both cell models were preincubated with the selective σ_1_ receptor ligand (+)-pentazocine (10 μM) for 2 h. Figure 5B shows that both, **19** and **29**, accumulate in both cell models in a dose-dependent manner. In good agreement with their supposed σ_2_ affinities, the signal was visibly weaker in the MCF7KO cells. It is noteworthy that both new ligands had fluorescence properties better than those of **3**, which was included as a reference, highlighting the superior fluorescent properties of the newly developed Cy-5-bearing derivatives. In addition, flow cytometric saturation binding experiments were performed with compounds **19** and **29** to define their *K*_d_ values in MCF7 cells. Increasing concentrations of the fluorescent ligands in the absence and presence of a fixed dose of two structurally different σ_2_ receptor reference ligands such as DTG (Figure S3) and compound **2**(50) (Figure S4) were studied. In particular, in the presence of DTG, compound **19** displayed a *K*_d_ of 13.59 nM, whereas in the presence of reference ligand **2**, compound **19** had a *K*_d_ of 19.32 nM. Compound **29** displayed a *K*_d_ of 18.66 nM in the presence of DTG, whereas its *K*_d_ was 13.82 nM in the presence of compound **2**. Thus, comparable *K*_d_ values were obtained with the two reference ligands. These data were also in line with the *K*_i_ values obtained from the radioligand binding assays (Table 1), thus supporting the validity of compounds **19** and **29** as σ_2_ receptor probes. Taking advantage of the *K*_d_ values obtained for each fluorescent ligand in the saturation experiment with DTG (Figure S3), we then set up a σ_2_ receptor binding assay. Binding curves were generated for DTG and compound **2**, using **19** or **29** (100 nM) in place of the radioligand (Figure S5). Upon displacement of compound **19**, the *K*_i_ value of DTG was 3.14 nM, whereas the *K*_i_ value of compound **2** was 4.07 nM. Upon displacement of compound **29**, the *K*_i_ value of DTG was 0.58 nM, whereas the *K*_i_ value of compound **2** was 5.04 nM. The data obtained were in the one-digit nanomolar range, reliably matching the values obtained with the radioligand binding assay, in particular for compound **2**.

Due to the dual nature of compound **19** that binds both receptor subtypes, MCF7 cells overexpressing the σ_1_ receptors (MCF7σ_1_), previously obtained by a stable transfection of MCF7 cells (*B*_max_ = 3.45 pmol/mg of protein), were used.^53,63^ In these experiments, 10 μM reference σ_2_ receptor ligand **2** was added to mask the σ_2_ receptor. Also in this experiment, a dose-dependent increase in the intensity of the fluorescent signal due to compound **19** was detected (Figure S6A). The uptake of **19** was abated upon the administration of (+)-pentazocine in the MCF7σ_1_ cells (Figure S6B). This result highlights the ability of **19** to label both σ receptors: it can be used in the presence of specific σ_2_ or σ_1_ ligands as a masking compound to label σ_1_ or σ_2_, respectively.

It is worth noting that, while the promising Cy-7-bearing compounds **20** and **30** (homologues of **19** and **29**, respectively) could not be studied because the instrument was not equipped with the proper laser, all of the other 4-DMAP-bearing new compounds (**16**, **17**, and **23**) generated dose-dependent signals in MCF7 cells, which were abated upon administration of **2**,^50^ in agreement with reference ligands **1** and **3**(51) (data not shown).

### Confocal Microscopy Studies

**Figure 6:**
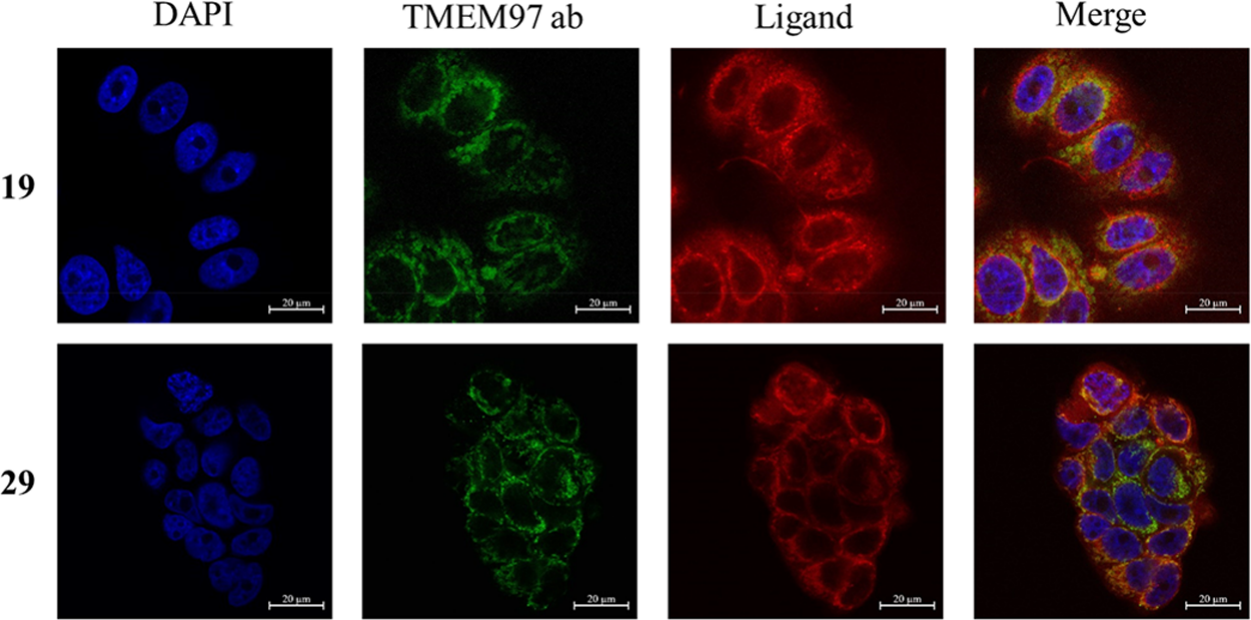
Representative confocal microscopy images showing the co-localization of **19** and **29** with σ_2_ receptor expression in MCF7 cells. Cells were preincubated with 10 μM selective σ_1_ receptor agonist (+)-pentazocine for 2 h to mask residual σ_1_ receptor expression. This was followed by incubation for 1 h with the indicated fluorescent ligands at 5 μM. Then cells were fixed with paraformaldehyde, stained for σ_2_ receptor expression (TMEM97 ab) as well as nuclei (by DAPI), and analyzed by confocal microscopy. The ligands are colored red (scale bar of 20 μm).

As a next step, the ability of **19** and **29** to visualize the σ_2_ receptor was investigated in MCF7 cells by confocal microscopy. The residual σ_1_ receptor was again masked by preincubating the cells with the selective σ_1_ receptor agonist (+)-pentazocine or 1-[2-(4-chlorophenoxy)ethyl]-4-methylpiperidine (**31**; *K*_i_ = 0.86 nM at the σ_1_ receptor; *K*_i_ = 239 nM at the σ_2_ receptor).^6^ The optimal experimental conditions were pre-evaluated using different setups, and the best performance was obtained by preincubation of the cells with 10 μM σ_1_ receptor-masking agent for 2 h followed by the administration of the fluorescent ligands. Trypan blue assays were performed to confirm that neither **19** nor **29** was cytotoxic under these conditions (data not shown). First, we checked the subcellular localization of the two fluorescent ligands. After incubation for 1 h with the fluorescent ligands (5 μM), cells were fixed with paraformaldehyde as described in the Experimental Section, stained for σ_2_ receptor expression, and analyzed by confocal microscopy (Figure 6). In line with previously published data, the σ_2_ receptor was localized around the nucleus, comparable to the immunostaining for TMEM97 (TMEM97ab).^47,48^ The two new fluorescent ligands displayed a similar pattern and overlapped with the σ_2_ receptor distribution (TMEM97ab), with compound **19** showing a slightly better overlap.

**Figure 7:**
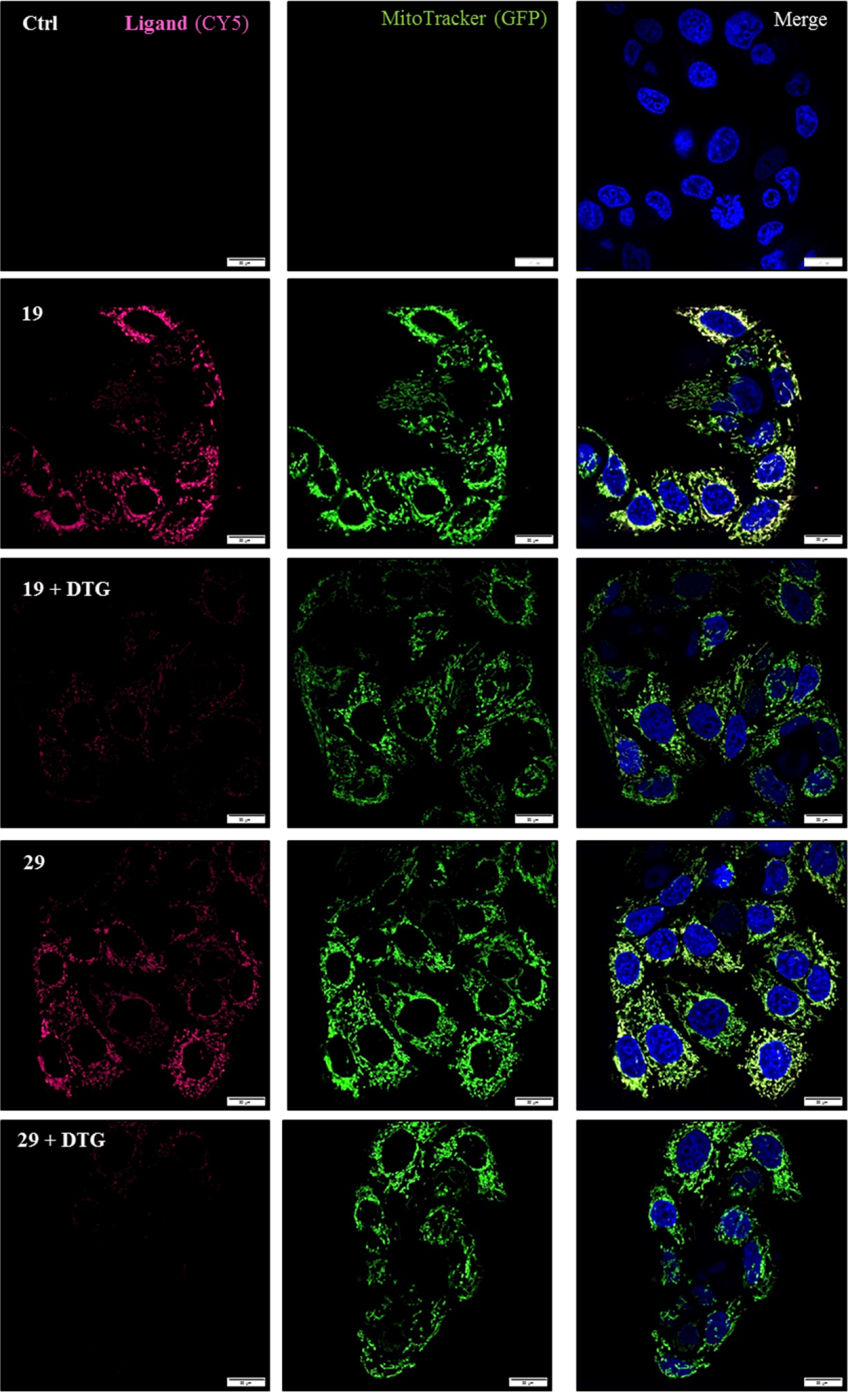
Representative spinning disk images showing the localization of ligand **19** as well as **29** with the mitochondria as well as reduction of the Cy-5 signal of the ligands in the presence of the nonfluorescent σ_2_ ligand DTG in living MCF7 cells. Cells were preincubated with 10 μM **31** to mask residual σ_1_ receptor expression with or without 20 μM DTG for 75 min. This was followed by administration of 0.05 μM **19** or **29** for 30 min (red stain in the Cy-5 channel). In addition, mitochondria were stained with 250 nM MitoTracker-Bodipy FL (green stain in the GFP channel) and the nuclei were counterstained with Hoechst 33342 (blue stain in the DAPI channel). The depicted scale bar indicates 20 μm.

Subsequent spinning disc confocal microscopy experiments on living MCF7 cells revealed a mitochondrial localization of **19** and **29** (Figure 7), which could be significantly reduced by co-treatment with nonfluorescent DTG in a competitive manner (Figure S7). Subsequently, to support the specific binding of compounds **19** and **29** on the σ_2_ receptor, MCF7 as well as MCF7KO cells were incubated with lower concentrations (0.01, 0.05, 0.1, and 1 μM) of the two compounds. The representative confocal microscopy images demonstrate a more pronounced staining at each concentration in MCF7, whereas in the MCF7KO cells, a weakened signal could be obtained (due to a partial knockdown of the σ_2_ receptor). This supports the specific binding of compounds **19** and **29** to the σ_2_ receptor (Figure S8).

**Figure 8:**
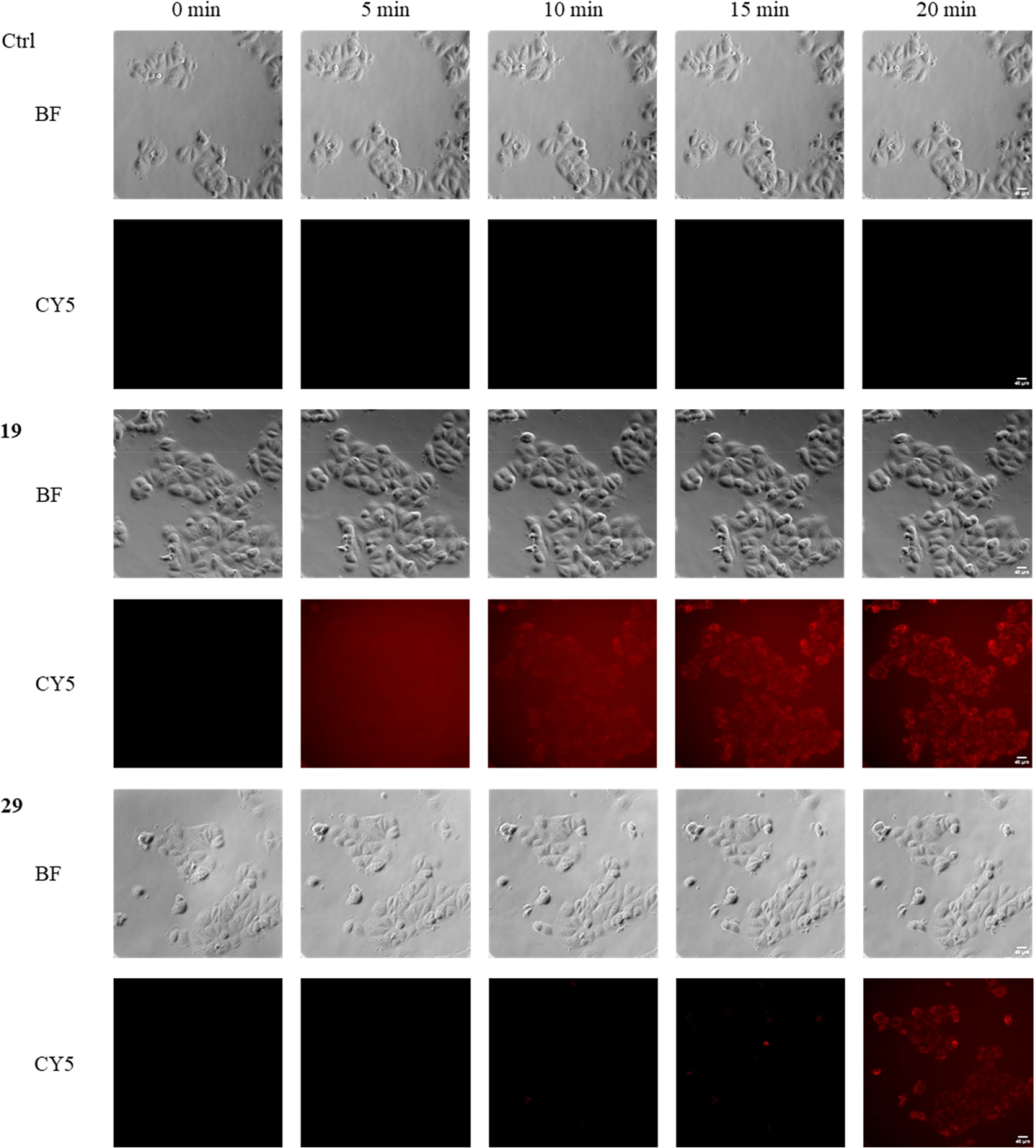
Representative microscopically live cell images showing time-dependent accumulation of ligand **19** as well as **29** in MCF7 cells from 0 to 20 min. Cells were preincubated with 10 μM selective σ_1_ receptor agonist **31** for 2 h to mask residual σ_1_ receptor expression. This was followed by administration of **19** and **29**. For time-lapse experiments, images were collected every 5 min for a period of 4 h. The bright field (BF) and the fluorescence channel Cy-5 for the ligands (red) are shown (scale bar of 40 μm).

To gain insight into the kinetics of the staining properties of our new ligands, live cell videos were performed, which showed the rapid entry of the ligands (**19** and **29**) into the cells with compound **19** showing a faster accumulation, indicating their potential use as staining tools also in this setting (Figure 8). Together, these experiments indicate that these new fluorescent ligands can be employed as potent tools for the investigation of the σ_2_ receptor in living cells. Importantly, the stability of the two compounds was demonstrated in PBS buffer at 37 °C by RP-HPLC over 24 h (Figures S9 and S10).

## Conclusions

There is a recognized need to widen the available repertoire of pharmacological tools to study σ receptor subtypes. To fill this gap, we introduced structural changes into siramesine (siramesine-like) and compound **8** (**8**-like) to develop high-affinity fluorescent σ_2_ receptor ligands, suitable for different fluorescence-based assays. While green-emitting ligands for both σ receptor subtypes are already known, no red- or NIR-emitting fluorescent σ ligands, notably characterized by better photophysical properties, have been reported, to the best of our knowledge. Flow cytometry and confocal microscopy studies in MCF7 cells (MCF7 wild type and MCF7KO) with red-emitting compounds **19** (siramesine-like) and **29** (**8**-like) demonstrated the specific targeting of the σ_2_ receptor, despite their improvable selectivity versus the σ_1_ subtype, which requires masking of the σ_1_ receptor as in the radioligand binding assay. Nevertheless, the lack of selectivity of the siramesine-like fluorescent ligand **19** may be exploited. We demonstrated that the compound probes the σ_1_ receptor in MCF7σ_1_ cells, thus resulting in a pan-σ receptor probe that can visualize both subtypes.

Additionally, the molecular rationale behind the discussed experimental observations was provided by the performed docking simulations. In particular, the obtained results (i) highlight the importance of specific interactions for the molecular recognition of both siramesine-like and **8**-like compounds and (ii) support, from a molecular point of view, the hypothesis whereby the strategy followed for designing the fluorescent probes presented in this study is not hindered by putative steric clashes within the σ receptor cavities. Interestingly, the full consistency between the computed MM-GBSA scores and the experimental data puts forward the computational protocol herein tuned as valuable for the rational design of new and better performing fluorescent ligands that can selectively probe the σ_2_ receptor subtype.

Overall, multiple advantages may be realized by exploiting these tools: detection of the receptors in live cells, localization of the receptors within the cells in their unmodified genome, evaluation of co-localizing or interacting proteins, and potential application *in vivo* by optical microscopy (NIR-emitting ligands). Lastly, the setup of novel binding assays, herein performed, highlights the power of these fluorescent ligands as probes to investigate the affinity of novel molecular entities in the absence of radioactive materials, with less danger and waste.

## Experimental Section

### Synthesis

All chemicals were purchased from Sigma-Aldrich, TCI Chemicals, Alfa Aesar, or Acros Organics. Thin layer chromatography (TLC) was performed using plates from Merck (silica gel 60 F254). Column chromatography was performed with Merck silica gel 60 Å [63–200 mm; 1:30 (w/w) crude mixture:silica gel ratio, unless otherwise stated] as the stationary phase. ^1^H NMR spectra were recorded in the indicated solvent on a Varian Mercury-VX spectrometer (300 MHz) or on an Agilent 500-vnmrs500 spectrometer (499.801 MHz). ^13^C NMR (75 MHz) spectra were recorded in the indicated solvent on a Varian Mercury-VX spectrometer (300 MHz). The following data are reported: chemical shift (δ) in parts per million, multiplicity (s, singlet; d, doublet; t, triplet; q, quartet; quint, quintet; m, multiplet; dd, doublet of doublets; dt, doublet of triplets; td, triplet of doublets; br, broad signal), integration, and coupling constant (*J*) in hertz. Mass spectra were recorded on a model HP6890-5973 MSD gas chromatograph/mass spectrometer; only significant *m*/*z* peaks, with their percentage of relative intensity in parentheses, are reported. HRMS-ESI analyses were performed on a Bruker Daltonics MicrOTOF-Q II mass spectrometer. All spectra were in accord with the assigned structures. The purity of target compounds was assessed by HPLC. Analytical HPLC analyses were performed on final compounds **16**–**20**, **23**, and **28**–**30** using a uHPLC Thermo Dionex Ultimate 3000 instrument with a WATERS XSelect PREMIER HSS T3 Column (100 Å, 2.5 μm, 2.1 mm × 100 mm) at a flow rate of 0.5 mL/min using a linear gradient of the mobile phase (CH_3_CN with 0.1% formic acid/H_2_O with 0.1% formic acid). The UV signal was detected at 220 and 250 nm. All compounds are >95% pure as determined by HPLC.

### 4-(1*H*-Indol-3-yl)-1-(3*H*-spiro[isobenzofuran-1,4′-piperidin]-1′-yl)butan-1-one (**9**)

To a solution of 4-(1*H*-indol-3-yl)butanoic acid (343 mg, 1.70 mmol, 1 equiv) in anhydrous THF (5 mL), kept under argon, was added CDI (356 mg, 2.20 mmol, 1.3 equiv). The mixture was stirred for 2 h at room temperature, and then 3*H*-spiro[isobenzofuran-1,4′-piperidine] (415 mg, 2.20 mmol, 1.3 equiv) in anhydrous THF (8 mL) was added. The reaction mixture was stirred overnight at room temperature. Subsequently, H_2_O was added, and the mixture was extracted with Et_2_O (2 × 10 mL) and EtOAc (2 × 10 mL). The organic layers were collected, dried (Na_2_SO_4_), and evaporated under reduced pressure to afford the crude mixture as a yellow oil. Purification by column chromatography with CH_2_Cl_2_/MeOH (from 98:2 to 95:5) as the eluent gave the title compound as a yellow oil (574 mg, 91% yield). GC-MS *m*/*z*: 374 (M^+^, 70), 231 (50), 146 (80), 130 (100). ^1^H NMR (500 MHz, CDCl_3_): δ 1.62–1.93 (m, 4H), 2.11 (quint, 2H, *J* = 7.3 Hz), 2.45 (t, 2H, *J* = 7.3 Hz), 2.78 (t, 2H, *J* = 6.7 Hz), 2.97–3.02 (m, 1H), 3.35–3.50 (m, 1H), 3.76 (d, 1H, *J* = 16 Hz), 4.64 (d, 1H, *J* = 11.5 Hz), 5.08 (s, 2H), 6.98 (s, 1H), 7.05 (d, 1H, *J* = 6 Hz), 7.15 (t, 1H, *J* = 7.2 Hz), 7.18–7.33 (m, 5H), 7.58–7.61 (m, 1H), 7.97 (br s, 1H, D_2_O exchanged).

### 3-{3-[4-Oxo-4-(3*H*-spiro[isobenzofuran-1,4′-piperidin]-1′-yl)butyl]-1*H*-indol-1-yl}propanenitrile (**10**)

Compound **9** (100 mg, 0.27 mmol, 1 equiv), 3-bromopropanenitrile (72 mg, 0.54 mmol, 2 equiv), KOH (22 mg, 0.392 mmol, 1.5 equiv), and K_2_CO_3_ (136 mg, 0.99 mmol, 3.5 equiv) were weighed altogether in a 2–5 mL microwave vial, and CH_3_CN (3 mL) was added. The vial was sealed, shaken vigorously, and heated in the microwave irradiator at 150 °C for 60 min. After the mixture had cooled, the solvent was removed under reduced pressure and the residue was taken up with H_2_O and extracted with CH_2_Cl_2_ (3 × 10 mL). The collected organic phases were dried (Na_2_SO_4_) and evaporated under reduced pressure to provide a dark oil that was purified by column chromatography (1:10) with CH_2_Cl_2_/MeOH (98:2) as the eluent that gave the title compound as a yellow oil (50 mg, 87% yield). GC-MS *m*/*z*: 427 (M^+^, 73.7), 231 (38.3), 146 (100). ^1^H NMR (500 MHz, CDCl_3_): δ 1.63–1.92 (m, 4H), 2.10 (quint, 2H, *J* = 7.5 Hz), 2.46 (t, 2H, *J* = 7.5 Hz), 2.76 (t, 2H, *J* = 6.7 Hz), 2.81–2.92 (m, 2H), 2.96–3.02 (m, 1H), 3.34–3.50 (m, 1H), 3.74 (d, 1H, *J* = 15.9 Hz), 4.37 (t, 2H, *J* = 6.7 Hz), 4.63 (d, 1H, *J* = 11.2 Hz), 5.07 (s, 2H), 6.98 (s, 1H), 7.04 (d, 1H, *J* = 6.1 Hz), 7.14 (t, 1H, *J* = 7.2 Hz), 7.17–7.33 (m, 5H), 7.64 (d, 1H, *J* = 7.7 Hz).

### 3-{3-[4-(3*H*-Spiro[isobenzofuran-1,4′-piperidin]-1′-yl)butyl]-1*H*-indol-1-yl}propan-1-amine (**11**)

A solution of **10** (170 mg, 0.40 mmol, 1 equiv) in anhydrous THF (10 mL) was added with a solution of 2 M BH_3_·DMS (1 mL, 2.0 mmol, 5 equiv) while being kept under a stream of N_2_ and cooled in an ice bath. After the addition, the mixture was refluxed for 3 h. After the mixture had cooled, MeOH (10 mL) and 3 N HCl were added, and the mixture was refluxed for 1 h. Then, the mixture was basified with 3 N NaOH, and the aqueous layer was extracted with CH_2_Cl_2_ (3 × 10 mL). The collected organic layers were dried (Na_2_SO_4_) and evaporated under reduced pressure to afford a crude oil as a dense yellow oil that was purified by column chromatography (1:10) with CH_2_Cl_2_/MeOH (from 98:2 to 80:20) as the eluent to afford the title compound as a pale-yellow oil (112 mg, 67% yield). GC-MS *m*/*z*: 417 (M^+^, 6.8), 202 (100). LC-MS (ESI^+^) *m*/*z*: 418 [M + H]^+^, 440 [M + Na]^+^, 456 [M + K]^+^ LC-MS-MS 418: 418 (15.57), 186 (100). LC-MS-MS 440: 440 (100), 186 (9.69). HRMS (ESI^+^) calcd for C_27_H_36_N_3_O [M + H]^+^, 418.2853; found, 418.2714. ^1^H NMR (500 MHz, CDCl_3_): δ 1.52–1.86 (m, 8H), 1.87–2.08 (m, 4H), 2.33–2.53 (m, 4H), 2.68 (t, 2H, *J* = 6.8 Hz), 2.80 (t, 2H, *J* = 7.4 Hz), 2.88 (d, 2H, *J* = 5.9 Hz), 4.14 (t, 2H, *J* = 6.8 Hz), 5.07 (s, 2H), 6.89 (s, 1H), 7.03–7.12 (m, 1H), 7.12–7.22 (m, 3H), 7.22–7.29 (m, 2H), 7.32 (d, 1H, *J* = 8.2 Hz), 7.61 (d, 1H, *J* = 7.7 Hz). ^13^C NMR (75 MHz, CDCl_3_): δ 25.01, 28.33, 31.17, 33.28, 35.95, 41.01, 46.05, 50.05, 58.55, 71.33, 82.40, 109.26, 114.85, 116.98, 118.43, 121.01, 121.05, 121.27, 125.05, 127.45, 127.72, 127.92, 136.35, 138.80, 143.38.

### 1′-[4-(1*H*-Indol-3-yl)butyl]-3*H*-spiro[isobenzofuran-1,4′-piperidine] (**12**)

A solution of **9** (150 mg, 0.40 mmol, 1 equiv) in anhydrous THF (10 mL) was added with a solution of 2 M BH_3_·DMS (1.20 mmol, 0.60 mL, 3 equiv) while being kept under a stream of N_2_ and cooled in an ice bath. After the addition, the mixture was refluxed for 3 h. After the mixture had cooled, MeOH (10 mL) and 3 N HCl were added, and the mixture was refluxed for 1 h. Then, the mixture was basified with 3 N NaOH, and the aqueous layer was extracted with CH_2_Cl_2_ (3 × 10 mL). The collected organic layers were dried (Na_2_SO_4_) and evaporated under reduced pressure to afford a crude oil as a dense yellow oil that was purified by column chromatography (1:10) with CH_2_Cl_2_/MeOH (from 98:2 to 80:20) as the eluent to afford the title compound as a pale-yellow oil (82 mg, 27% yield). GC-MS *m*/*z*: 360 (M^+^, 13), 202 (100), 130 (15), 42 (10). ^1^H NMR (500 MHz, CDCl_3_): δ 1.65–1.73 (m, 2H), 1.73–1.83 (m, 4H), 2.03 (td, 2H, *J* = 13.0, 4.9 Hz), 2.45 (t, 2H, *J* = 11.74 Hz), 2.50 (t, 2H, *J* = 7.82 Hz), 2.81 (t, 2H, *J* = 7.34 Hz), 2.93 (d, 2H, *J* = 9.3 Hz), 5.1 (s, 2H), 6.97 (s, 1H), 7.10–7.15 (m, 2H), 7.16–7.24 (m, 2H), 7.24–7.30 (m, 2H), 7.34 (d, 1H, *J* = 8.31 Hz), 7.64 (d, 1H, *J* = 7.83 Hz), 8.2 (br s, 1H).

### 1′-{4-[1-(6-Bromohexyl)-1*H*-indol-3-yl]butyl}-3*H*-spiro[isobenzofuran-1,4′-piperidine] (**13**)

A solution of **12** (159 mg, 0.44 mmol, 1 equiv), tetrabutylammonium bromide (TBAB, 4 mg, 0.013 mmol, 0.03 equiv), and KOH (99 mg, 1.77 mmol, 4 equiv) in anhydrous DMF was stirred at room temperature for 45 min. Subsequently, the solution was cooled to 0 °C and then 1,6-dibromohexane (162 mg, 0.66 mmol, 1.5 equiv) was added. The mixture was stirred at 0 °C for 15 min and then at room temperature for 1 h. Then, the solvent was removed under reduced pressure, and the residue was taken up with H_2_O and extracted with CH_2_Cl_2_ (3 × 10 mL). The collected organic phases were dried (Na_2_SO_4_) and evaporated under reduced pressure to provide a dark oil that was purified by column chromatography (1:15) with CH_2_Cl_2_ as the eluent to give the title compound as a pale-yellow oil (188 mg, 81% yield). LC-MS (ESI^+^): 523–525 [M + H]^+^, 545–547 [M + Na]^+^. LC-MS-MS 525: 525 (72.21), 523 (48.01), 336 (100), 334 (84.46). ^1^H NMR (500 MHz, CDCl_3_): δ 1.20–1.57 (m, 8H), 1.68–1.80 (m, 6H), 2.18 (td, 2H, *J* = 13.0, 4.5 Hz), 2.43–2.50 (m, 6H), 2.91–3.12 (m, 2H), 3.50 (t, 2H, *J* = 7.4 Hz), 4.10 (t, 2H, *J* = 7.1 Hz), 5.05 (s, 2H), 6.40 (s, 1H), 7.00 (t, 1H, *J* = 7.5 Hz), 7.15–7.19 (m, 4H), 7.23–7.30 (m, 2H), 7.56 (d, 1H, *J* = 7.7 Hz).

### 1′-{4-[1-(6-Azidohexyl)-1*H*-indol-3-yl]butyl}-3*H*-spiro[isobenzofuran-1,4′-piperidine] (**14**)

A solution of **13** (188 mg, 0.36 mmol, 1 equiv) and NaN_3_ (67 mg, 1.8 mmol, 5.0 equiv) in anhydrous DMF (5 mL) was stirred at 60 °C for 20 h. The solvent was removed under reduced pressure. The residue was taken up with H_2_O and extracted with CH_2_Cl_2_ (3 × 10 mL). The combined organic layers were collected and washed with brine (3 × 10 mL), dried (Na_2_SO_4_), and evaporated under reduced pressure to provide a crude oil that was used without further purification (175 mg, 100% yield). ^1^H NMR (500 MHz, CDCl_3_): δ 1.21–1.55 (m, 8H), 1.67–1.80 (m, 6H), 2.17 (td, 2H, *J* = 13.3, 4.6 Hz), 2.43–2.50 (m, 6H), 2.80 (t, 2H, *J* = 7.3 Hz), 2.91–3.12 (m, 2H), 4.10 (t, 2H, *J* = 7.1 Hz), 5.05 (s, 2H), 6.40 (s, 1H), 7.02 (t, 1H, *J* = 7.5 Hz), 7.12–7.18 (m, 4H), 7.20–7.30 (m, 2H), 7.54 (d, 1H, *J* = 7.8 Hz).

### 6-{3-[4-(3*H*-Spiro[isobenzofuran-1,4′-piperidin]-1′-yl)butyl]-1*H*-indol-1-yl}hexan-1-amine (**15**)

A solution of compound **14** (183 mg, 0.38 mmol, 1 equiv) and PPh_3_ (199 mg, 0.76 mmol, 2 equiv) in anhydrous MeOH under argon was stirred at 80 °C for 60 min. The solvent was removed under reduced pressure. The residue was taken up with a solution of 2 N NaOH (10 mL) and extracted with CH_2_Cl_2_ (3 × 10 mL). The combined organic layers were collected, washed with brine (3 × 10 mL), dried (Na_2_SO_4_), and evaporated under reduced pressure to provide a crude oil that was purified by column chromatography (1:10) with CH_2_Cl_2_/MeOH (from 95:5 to 80:20) to afford the title compound as a colorless oil (109 mg, 62% yield). HRMS (ESI^+^) calcd for C_30_H_42_N_3_O [M + H]^+^, 460.3322; found, 460.3314. ^1^H NMR (500 MHz, CDCl_3_): δ 1.23–1.86 (m, 12H), 2.05 (td, 2H, *J* = 13.3, 4.6 Hz), 2.37–2.59 (m, 8H), 2.66 (t, 2H, *J* = 7.0 Hz), 2.78 (t, 2H, *J* = 7.3 Hz), 2.85–2.95 (m, 2H), 4.05 (t, 2H, *J* = 7.1 Hz), 5.07 (s, 2H), 6.88 (s, 1H), 7.08 (t, 1H, *J* = 7.5 Hz), 7.12–7.23 (m, 3H), 7.24–7.31 (m, 3H), 7.59 (d, 1H, *J* = 7.8 Hz). ^13^C NMR (75 MHz, CDCl_3_): δ 24.93, 26.18, 26.42, 26.77, 28.23, 30.17, 32.49, 35.93, 41.59, 46.02, 50.02, 58.54, 70.85, 84.21, 109.26, 114.83, 116.96, 118.41, 120.94, 121.02, 121.25, 125.06, 127.46, 127.71, 127.96, 136.36, 138.78, 145.01.

### General Procedure for the Synthesis of Final Compounds **16** and **17**

To a solution of 4-(dimethylamino)phthalic acid (57 mg, 0.27 mmol, 1 equiv) in anhydrous DMF (2 mL), kept under argon, was added CDI (100 mg, 0.62 mmol, 2.3 equiv). The mixture was stirred for 2 h at room temperature, and then either alkyl amine **11** or **15** (0.27 mmol, 1 equiv) in anhydrous DMF (3 mL) was added. The reaction mixture was stirred overnight at room temperature. Subsequently, H_2_O was added, and the aqueous phase was extracted with CH_2_Cl_2_ (3 × 10 mL). The organic layers were collected, dried (Na_2_SO_4_), and evaporated under reduced pressure to afford the crude mixture as a yellow oil. Purification by column chromatography with CH_2_Cl_2_/MeOH (95:5) as the eluent provided the title compounds as yellow oils that were then transformed into the corresponding oxalate salt. The oxalate salt was obtained by adding a solution of oxalic acid (1.5 equiv) in Et_2_O to a solution of the free amine in CH_2_Cl_2_. The obtained solid was filtered and recrystallized in MeOH/Et_2_O to achieve the oxalate salt as yellow crystals in 80% yield.

### 2-(3-{3-[4-(3*H*-Spiro[isobenzofuran-1,4′-piperidin]-1′-yl)butyl]-1*H*-indol-1-yl}propyl)-5-(dimethylamino)isoindoline-1,3-dione (**16**)

The free base was obtained in 22% yield. ^1^H NMR (500 MHz, CDCl_3_ performed on the free base): δ 1.74–1.84 (m, 6H), 2.10–2.27 (m, 4H), 2.42–2.60 (m, 4H), 2.72–2.83 (m, 2H), 2.96–3.06 (m, 2H), 3.10 (s, 6H), 3.69 (t, 2H, *J* = 6.7 Hz), 4.12 (t, 2H, *J* = 7.1 Hz), 5.02 (s, 2H), 6.77 (dd, *J* = 8.5, 2.1 Hz, 1H), 6.98–7.11 (m, 3H), 7.13–7.22 (m, 3H), 7.22–7.33 (m, 3H), 7.57 (d, 1H, *J* = 7.8 Hz), 7.63 (d, 1H, *J* = 8.5 Hz). LC-MS (ESI^+^) *m*/*z*: 591 [M + H]^+^, 613 [M + Na]^+^. LC-MS-MS 591: 591 (100), 402 (55.99), 231 (78.43). The oxalate salt was >98% pure as determined by HPLC analysis performed by isocratic elution with 80:20 (v/v) CH_3_CN/HCOONH_4_ (20 mM, pH 5.5) at a flow rate of 1 mL/min.

### 2-(6-{3-[4-(3*H*-Spiro[isobenzofuran-1,4′-piperidin]-1′-yl)butyl]-1*H*-indol-1-yl}hexyl)-5-(dimethylamino)isoindoline-1,3-dione (**17**)

The free base was obtained in 15% yield. ^1^H NMR (500 MHz, CDCl_3_ performed on the free base): δ 1.74–1.90 (m, 12H), 2.10–2.27 (m, 4H), 2.42–2.60 (m, 4H), 2.72–2.83 (m, 2H), 2.95–3.03 (m, 2H), 3.11 (s, 6H), 3.71 (t, 2H, *J* = 6.7 Hz), 4.10 (t, 2H, *J* = 7.1 Hz), 5.02 (s, 2H), 6.78 (dd, *J* = 8.5, 2.1 Hz, 1H), 6.98–7.11 (m, 3H), 7.13–7.22 (m, 3H), 7.21–7.33 (m, 3H), 7.58 (d, 1H, *J* = 7.8 Hz), 7.62 (d, 1H, *J* = 8.5 Hz). LC-MS (ESI^+^): 633 [M + H]^+^, 655 [M + Na]^+^. LC-MS-MS 633: 633 (100), 444 (67.84). LC-MS-MS 655: 655 (2.94), 460 (100).

### 5-(Dimethylamino)-2-(1*H*-indol-6-yl)isoindoline-1,3-dione (**21**)

To a solution of 4-(dimethylamino)phthalic acid (398 mg, 1.90 mmol, 1 equiv) in anhydrous DMF (6 mL), kept under argon, was added CDI (710 mg, 4.37 mmol, 2.3 equiv). The mixture was stirred for 2 h at room temperature, and then 1*H*-indol-6-amine (250 mg, 1.90 mmol, 1 equiv) in anhydrous DMF (1 mL) was added. The reaction mixture was stirred overnight at room temperature. Subsequently, water was added, and the aqueous phase was extracted with CH_2_Cl_2_ (3 × 10 mL). The organic layers were collected, dried (Na_2_SO_4_), and evaporated under reduced pressure to afford the crude mixture as a yellow oil. Purification by column chromatography with CH_2_Cl_2_ as the eluent gave the title compound as a yellow oil (107 mg, 18% yield). ^1^H NMR (300 MHz, CDCl_3_): δ 3.15 (s, 6H), 6.58 (s, 1H), 6.86 (dd, 1H, *J*_1_ = 8.8 Hz, *J*_2_ = 2.35 Hz), 7.12–7.18 (m, 3H), 7.43 (s, 1H), 7.70–7.77 (m, 2H), 8.29 (s, 1H).

### 2-[1-(4-Chlorobutyl)-1*H*-indol-6-yl]-5-(dimethylamino)isoindoline-1,3-dione (**22**)

A solution of **21** (107 mg, 0.351 mmol, 1 equiv) and potassium *tert*-butoxide (*t*-BuOK, 79 mg, 0.702 mmol, 2 equiv) in anhydrous DMF (3 mL) was stirred at 10 °C for 15 min. Subsequently, 1-bromo-4-chloro-butane (0.09 mL, 0.702 mmol, 2 equiv) was added, and the mixture was stirred at room temperature for 1 h. Then, the solvent was removed under reduced pressure, and the residue was taken up with H_2_O and extracted with CH_2_Cl_2_ (3 × 10 mL). The collected organic phases were dried (Na_2_SO_4_) and evaporated under reduced pressure to provide a dark oil that was purified by column chromatography with CH_2_Cl_2_ as the eluent to give the title compound as a pale-yellow oil (40 mg, 29% yield). ^1^H NMR (500 MHz, CDCl_3_): δ 1.75 (quint, 2H, *J* = 6.36 Hz), 2.0 (quint, 2H, *J* = 6.85 Hz), 3.14 (s, 6H), 3.50 (t, 2H, *J* = 6.36 Hz), 4.15 (t, 2H, *J* = 6.85 Hz), 6.52 (d, 1H, *J* = 2.94 Hz), 6.85 (dd, 1H, *J*_1_ = 8.81 Hz, *J*_2_ = 2.94 Hz), 7.11–7.17 (m, 3H), 7.38 (s, 1H), 7.69 (d, 1H, *J* = 8.32 Hz), 7.75 (d, 1H, *J* = 8.32 Hz).

### 2-{1-[4-(3*H*-Spiro[isobenzofuran-1,4′-piperidin]-1′-yl)butyl]-1*H*-indol-6-yl}-5-(dimethylamino)isoindoline-1,3-dione (**23**)

A solution of **22** (40 mg, 0.101 mmol, 1 equiv), 3*H*-spiro[isobenzofuran-1,4′-piperidine] (23 mg, 0.12 mmol, 1.2 equiv), and K_2_CO_3_ (17 mg, 0.12 mmol, 1.2 equiv) in 8 mL of CH_3_CN was heated at reflux overnight. After the mixture had cooled, the solvent was removed under reduced pressure, and then H_2_O (10 mL) was added to the residue that was extracted with CH_2_Cl_2_ (2 × 10 mL) and EtOAc (2 × 10 mL). The collected organic layers were dried (Na_2_SO_4_) and evaporated to afford a residue that was purified by column chromatography with CH_2_Cl_2_/MeOH (95:5) as the eluent to give the final compound as a yellow oil (27 mg, 49% yield). LC-MS (ESI^+^): 549 [M + H]^+^, 571 [M + Na]^+^. LC-MS-MS 549: 549 (100). 417 (13.16), 360 (25.6), 244 (16.24). ^1^H NMR (500 MHz, CDCl_3_): δ 1.61–1.83 (m, 4H), 1.91 (quint, 2H, *J* = 7.34 Hz), 2.04–2.20 (m, 2H), 2.39–2.47 (m, 4H), 2.80–2.90 (m, 2H), 3.15 (s, 6H), 4.16 (t, 2H, *J* = 6.85 Hz), 5.05 (s, 2H), 6.51 (d, 1H, *J* = 2.93 Hz), 6.86 (dd, 1H, *J*_1_ = 8.32 Hz, *J*_2_ = 2.45 Hz), 7.10 (dd, 1H, *J*_1_ = 8.32 Hz, *J*_2_ = 1.95 Hz), 7.16–7.20 (m, 4H), 7.20–7.25 (m, 2H), 7.38 (s, 1H), 7.69 (d, 1H, *J* = 8.32 Hz), 7.75 (d, 1H, *J* = 8.32 Hz).

### 6-(2,5-Dimethyl-1*H*-pyrrol-1-yl)-1*H*-indole (**24**)

A solution of 1*H*-indol-6-amine (225 mg, 1.70 mmol) and 2,5-hexanedione (378 μL, 3.24 mmol, 1.9 equiv) in dry toluene (10 mL) was refluxed for 6 h with a Dean-Stark apparatus. The solvent was evaporated under vacuum, and the crude oil was purified by chromatography with EtOAc as the eluent to give the final compound as a brown oil (335 mg, 94% yield). GC-MS *m*/*z*: 210 (M^+^, 100), 195 (20.7), 168 (20.8), 154 (23.1). ^1^H NMR (300 MHz, CDCl_3_): δ 2.04 (s, 6H), 5.91 (s, 2H), 7.10–7.20 (m, 3H), 7.41 (s, 1H), 7.80 (d, 1H, *J* = 7.5 Hz), 8.30 (br s, 1H).

### 1-(4-Chlorobutyl)-6-(2,5-dimethyl-1*H*-pyrrol-1-yl)-1*H*-indole (**25**)

A solution of **24** (335 mg, 1.60 mmol, 1 equiv), tetrabutylammonium bromide (TBAB, 16 mg, 0.05 mmol, 0.03 equiv), and KOH (368 mg, 6.40 mmol, 4 equiv) in anhydrous DMF was stirred at room temperature for 45 min. Subsequently, the solution was cooled to 0 °C, and then 1-bromo-4-chloro-butane (277 μL, 2.40 mmol, 1.5 equiv) was added. The mixture was stirred at 0 °C for 15 min and then at room temperature for 1 h. Then, the solvent was removed under reduced pressure, and the residue was taken up with H_2_O and extracted with CH_2_Cl_2_ (3 × 10 mL). The collected organic phases were dried (Na_2_SO_4_) and evaporated under reduced pressure to provide a dark oil that was purified by column chromatography with *n*-hexane/EtOAc (9:1) as the eluent to give the title compound as a brown oil (465 mg, 97% yield). GC-MS *m*/*z*: 302 (30.8), 300 (M^+^, 100), 223 (19.1), 111 (22.9). ^1^H NMR (300 MHz, CDCl_3_): δ 1.70 (quint, 2H, *J* = 6.4 Hz), 1.98 (quint, 2H, *J* = 6.8 Hz), 2.05 (s, 6H), 3.51 (t, 2H, *J* = 6.4 Hz), 4.18 (t, 2H, *J* = 6.5 Hz), 5.91 (s, 2H), 7.10–7.20 (m, 3H), 7.41 (s, 1H), 7.82 (d, 1H, *J* = 7.6 Hz).

### 1′-{4-[6-(2,5-Dimethyl-1*H*-pyrrol-1-yl)-1*H*-indol-1-yl]butyl}-3*H*-spiro[isobenzofuran-1,4′-piperidine] (**26**)

A solution of **25** (144 mg, 0.49 mmol, 1 equiv), 3*H*-spiro[isobenzofuran-1,4′-piperidine] (111 mg, 0.59 mmol, 1.2 equiv), and K_2_CO_3_ (81 mg, 0.59 mmol, 1.2 equiv) in 10 mL of CH_3_CN was heated at reflux overnight. After the mixture had cooled, the solvent was removed under reduced pressure, and then H_2_O (10 mL) was added to the residue and the mixture extracted with CH_2_Cl_2_ (2 × 10 mL) and EtOAc (2 × 10 mL). The collected organic layers were dried over Na_2_SO_4_ and evaporated to afford a residue that was purified by column chromatography with *n*-hexane/EtOAc/MeOH (5:4.5:0.5) as the eluent to give the final compound as a yellow oil (111 mg, 51% yield). LC-MS (ESI^+^): 454 [M + H]^+^, 476 [M + Na]^+^, 492 [M+K]^+^. LC-MS-MS 454: 454 (59.11). 265 (100), 263 (75.3), 244 (73.55). LC-MS-MS 476: 476 (100).

### 1-[4-(3*H*-Spiro[isobenzofuran-1,4′-piperidin]-1′-yl)butyl]-1*H*-indol-6-amine (**27**)

Compound **26** (111 mg, 0.24 mmol, 1.0 equiv) and hydroxylamine hydrochloride (170 mg, 0.24 mmol, 10 equiv) were weighed altogether in a 2–5 mL microwave vial, and a mixture of EtOH and H_2_O (3 mL, 2:1) was added. The vial was sealed, shaken vigorously, and heated in the microwave irradiator at 150 °C for 60 min. After the mixture had cooled, the solvent was removed under reduced pressure, and the residue was taken up with H_2_O and extracted with CH_2_Cl_2_ (3 × 10 mL). The collected organic phases were dried (Na_2_SO_4_) and evaporated under reduced pressure to provide a dark oil that was purified by column chromatography (1:15) with CH_2_Cl_2_/MeOH (from 98:2 to 80:20) as the eluent to give the title compound as a yellow oil (80 mg, 88% yield). LC-MS (ESI^+^): 376 [M + H]^+^, 398 [M + Na]^+^. LC-MS-MS 376: 376 (5.77), 244 (46.81), 187 (100), 84 (23.76). LC-MS-MS 398: 398 (100), 76 (19.86). HRMS (ESI) calcd for C_24_H_30_N_3_O [M + H]^+^, 376.2383; found, 376.2370. ^1^H NMR (500 MHz, CDCl_3_): δ 1.61 (quint, 2H, *J* = 10.2 Hz), 1.74–1.81 (m, 2H), 1.86 (quint, 2H, *J* = 7.3 Hz), 2.03 (td, 2H, *J*_1_ = 13.2 Hz, *J*_2_ = 4.4 Hz), 2.37–2.49 (m, 4H), 2.83–2.90 (m, 2H), 3.60 (br s, 2H), 4.04 (t, 2H, *J* = 7.1 Hz), 5.07 (s, 2H), 6.36 (d, 1H, *J* = 3.1 Hz), 6.56 (dd, 1H, *J* = 8.3, 2.0 Hz), 6.65 (d, 1H, *J* = 2.0 Hz), 6.91 (d, 1H, *J* = 3.2 Hz), 7.18–7.24 (m, 1H), 7.19–7.24 (m, 1H), 7.25–7.31 (m, 2H), 7.40 (d, 1H, *J* = 8.3 Hz).

### General Procedure for the Preparation of BDP-TR Fluorescent Derivatives **18** and **28**

BDP-TR-COOH (8.51 mg, 0.020 mmol, 1 equiv), DIPEA (10.5 μL, 0.06 mmol, 3 equiv), and HATU (11.44 g, 0.03 mmol, 1.5 equiv) were dissolved in CH_2_Cl_2_ (400 μL), and the solution was stirred at 30 °C for 10 min. The appropriate amine, either **15** or **27** (0.022 mmol, 1.1 equiv), was dissolved in CH_2_Cl_2_ (400 μL) and added to the solution, and the mixture was stirred at 30 °C overnight. Subsequently, H_2_O (5 mL) was added, and the aqueous phase was extracted with DCM (3 × 5 mL). The collected organic phases were dried (Na_2_SO_4_) and evaporated under reduced pressure. The reaction mixture was purified by preparative TLC with CH_2_Cl_2_/MeOH (95:5) to afford the title compound as a purple solid.

### *N*-(6-{3-[4-(3*H*-Spiro[isobenzofuran-1,4′-piperidin]-1′-yl)butyl]-1*H*-indol-1-yl}hexyl)-2-{4-[5,5-difluoro-7-(thiophen-2-yl)-5*H*-5λ^4^,6λ^4^-dipyrrolo[1,2-*c*:2′,1′-*f*][1,3,2]diazaborinin-3-yl]phenoxy}acetamide (**18**)

The title compound was obtained in a 44% yield as a purple solid. ^1^H NMR (300 MHz, CDCl_3_): δ 1.22–1.98 (m, 14H), 2.07–2.33 (m, 2H), 2.57–2.84 (m, 4H), 3.01–3.21 (s, 2H), 3.25–3.80 (m, 4H), 4.04 (t, 2H, *J* = 7.1 Hz), 4.54 (s, 2H), 5.05 (s, 2H), 6.56 (br s, 1H), 6.63 (d, 1H, *J* = 4.5 Hz), 6.81 (d, 1H, *J* = 4.3 Hz), 6.89 (s, 1H), 6.96–7.22 (m, 9H), 7.23–7.32 (s, 4H), 7.43–7.48 (m, 1H), 7.56 (d, 1H, *J* = 7.9 Hz), 7.96 (d, 2H, *J* = 8.9 Hz), 8.10 (d, 1H, *J* = 3.9 Hz). HRMS (ESI) calcd for C_51_H_55_BF_2_N_5_O_3_S [M + H]^+^, 866.4081; found, 866.4080.

### *N*-{1-[4-(3*H*-Spiro[isobenzofuran-1,4′-piperidin]-1′-yl)butyl]-1*H*-indol-6-yl}-2-{4-[5,5-difluoro-7-(thiophen-2-yl)-5*H*-5λ^4^,6λ^4^-dipyrrolo[1,2-*c*:2′,1′-*f*][1,3,2]diazaborinin-3-yl]phenoxy}acetamide (**28**)

The title compound was obtained in a 56% yield as a purple solid. ^1^H NMR (300 MHz, CDCl_3_): δ 1.50–1.67 (m, 2H), 1.68–2.08 (m, 6H), 2.29–2.53 (m, 4H), 2.76–2.90 (m, 2H), 4.16 (t, 2H, *J* = 7.0 Hz), 4.70 (s, 2H), 5.04 (s, 2H), 6.45 (d, 1H, *J* = 3.1 Hz), 6.65 (d, 1H, *J* = 4.3 Hz), 6.81 (d, 1H, *J* = 4.3 Hz), 6.98 (d, 1H, *J* = 8.4 Hz), 7.02–7.30 (m, 11H), 7.46 (d, 1H, *J* = 5.1 Hz), 7.56 (d, 1H, *J* = 8.4 Hz), 7.94–8.06 (m, 3H), 8.06–8.15 (m, 1H), 8.36 (s, 1H). HRMS (ESI) calcd for C_54_H_43_BF_2_N_5_O_3_S [M + H]^+^, 782.3142; found, 782.3148.

### General Procedure for the Preparation of Cy-5 Fluorescent Derivatives **19** and **29**

Cy-5-COOH (12.21 mg, 0.020 mmol, 1 equiv), DIPEA (10.5 μL, 0.06 mmol, 3 equiv), and HATU (11.44 g, 0.03 mmol, 1.5 equiv) were dissolved in dry CH_2_Cl_2_ (500 μL), and the obtained solution was stirred at 30 °C for 10 min. The appropriate amine, either **15** or **27** (0.022 mmol, 1.1 equiv), was dissolved in dry CH_2_Cl_2_ (500 μL) and added to the solution, and the mixture was stirred at 30 °C for 12 h. Subsequently, H_2_O (5 mL) was added, and the mixture was extracted with CH_2_Cl_2_ (3 × 5 mL). The collected organic phases were dried (Na_2_SO_4_) and evaporated under reduced pressure. The mixture was purified by preparative TLC with CH_2_Cl_2_/MeOH (94:6) to afford the title compound as a dark-blue solid.

### 2-{(1*E*,3*E*)-5-[(*E*)-1-{6-[(6-{3-[4-(3*H*-Spiro[isobenzofuran-1,4′-piperidin]-1′-yl)butyl]-1*H*-indol-1-yl}hexyl)amino]-6-oxohexyl}-3,3-dimethylindolin-2-ylidene]penta-1,3-dien-1-yl}-1,3,3-trimethyl-3*H*-indol-1-ium (**19**)

The title compound was obtained in a 52% yield as a dark-blue solid. ^1^H NMR (300 MHz, CDCl_3_): δ 1.42–2.38 (m, 36H), 2.60–2.83 (m, 6H), 3.03–3.30 (m, 4H), 3.53 (s, 3H), 3.96 (t, 2H, *J* = 7.7 Hz), 4.05 (t, 2H, *J* = 6.9 Hz), 5.06 (s, 2H), 6.11 (br s, 1H), 6.17–6.27 (m, 2H), 6.72–6.87 (m, 1H), 6.95 (s, 1H), 7.01–7.47 (m, 15H), 7.55 (d, 1H, *J* = 8.0 Hz), 7.72–7.88 (m, 2H). HRMS (ESI) calcd for C_62_H_79_N_5_O_2_ [M + H]^2+^, 462.8111; found, 462.8108.

### 2-[(1*E*,3*E*)-5-{(*Z*)-1-[6-({1-[4-(3*H*-Spiro[isobenzofuran-1,4′-piperidin]-1′-yl)butyl]-1*H*-indol-6-yl}amino)-6-oxohexyl]-3,3-dimethylindolin-2-ylidene}penta-1,3-dien-1-yl]-1,3,3-trimethyl-3*H*-indol-1-ium (**29**)

The title compound was obtained in a 55% yield as a dark-blue solid. ^1^H NMR (300 MHz, CDCl_3_): δ 1.59–1.72 (m, 12H), 1.74–1.93 (m, 12H), 1.97–2.12 (m, 2H), 2.40–2.66 (m, 6H), 2.89–3.02 (m, 2H), 3.50 (s, 3H), 3.98 (t, 2H, *J* = 7.5 Hz), 4.10 (t, 2H, *J* = 7.0 Hz), 5.03 (s, 2H), 6.18 (t, 2H, *J* = 14.3 Hz), 6.37 (d, 1H, *J* = 3.1 Hz), 6.67–6.80 (m, 1H), 6.98–7.15 (m, 4H), 7.15–7.28 (m, 6H), 7.28–7.41 (m, 4H), 7.44 (d, 1H, *J* = 8.5 Hz), 7.77 (t, 2H, *J* = 13.0 Hz), 7.88 (s, 1H), 8.02 (s, 1H). HRMS (ESI) calcd for C_56_H_67_N_5_O_2_ [M + H]^2+^, 420.7642; found, 420.7647.

### General Procedure for the Preparation of Cy-7 Fluorescent Derivatives **20** and **30**

Cy-7-COOH (12.73 mg, 0.020 mmol, 1 equiv), DIPEA (10.5 μL, 0.06 mmol, 3 equiv), and HATU (11.44 g, 0.03 mmol, 1.5 equiv) were dissolved in dry CH_2_Cl_2_ (500 μL), and the solution was stirred at 30 °C for 10 min. The appropriate amine, either **15** or **27** (0.022 mmol, 1.1 equiv), was dissolved in dry CH_2_Cl_2_ (500 μL) and added to the solution, and the mixture was stirred at 30 °C overnight. Subsequently, H_2_O (5 mL) was added, and the mixture was extracted with CH_2_Cl_2_ (3 × 5 mL). The collected organic phases were dried (Na_2_SO_4_) and evaporated under reduced pressure to provide a dark-red solid that was purified by preparative TLC with CH_2_Cl_2_/MeOH (94:6) to afford the title compound as a dark-green solid.

### 2-{(1*E*,3*E*,5*E*)-7-[(*E*)-1-{6-[(6-{3-[4-(3*H*-Spiro[isobenzofuran-1,4′-piperidin]-1′-yl)butyl]-1*H*-indol-1-yl}hexyl)amino]-6-oxohexyl}-3,3-dimethylindolin-2-ylidene]hepta-1,3,5-trien-1-yl}-1,3,3-trimethyl-3*H*-indol-1-ium (**20**)

The title compound was obtained in a 53% yield as a dark-green solid. ^1^H NMR (300 MHz, CDCl_3_): δ 1.43–2.32 (m, 36H), 2.64–2.83 (m, 6H), 3.05–3.26 (m, 4H), 3.46 (s, 3H), 3.94 (t, 2H, *J* = 7.6 Hz), 4.05 (t, 2H, *J* = 7.0 Hz), 5.05 (s, 2H), 5.92–6.07 (m, 2H), 6.19 (d, 1H, *J* = 13.6 Hz), 6.50 (t, 1H, *J* = 13.0 Hz), 6.64 (t, 1H, *J* = 12.4 Hz), 6.78–7.43 (m, 16H), 7.43–7.60 (m, 2H), 7.67–7.89 (m, 2H). HRMS (ESI) calcd for C_64_H_81_N_5_O_2_ [M + H]^2+^, 475.8190; found, 475.8198.

### 2-[(1*E*,3*E*,5*E*)-7-{(*Z*)-1-[6-({1-[4-(3*H*-Spiro[isobenzofuran-1,4′-piperidin]-1′-yl)butyl]-1*H*-indol-6-yl}amino)-6-oxohexyl]-3,3-dimethylindolin-2-ylidene}hepta-1,3,5-trien-1-yl]-1,3,3-trimethyl-3*H*-indol-1-ium (**30**)

The title compound was obtained in a 59% yield as a dark-green solid. ^1^H NMR (300 MHz, CDCl_3_): δ 1.50–1.72 (m, 14H), 1.73–2.23 (m, 12H), 2.48 (t, 2H, *J* = 7.1 Hz), 2.55–2.73 (m, 4H), 2.92–3.06 (m, 2H), 3.49 (s, 3H), 3.98 (t, 2H, *J* = 7.5 Hz), 4.11 (t, 2H, *J* = 7.0 Hz), 5.03 (s, 2H), 5.94 (d, 1H, *J* = 13.5 Hz), 6.18 (d, 1H, *J* = 13.7 Hz), 6.29–6.45 (m, 2H), 6.55 (t, 1H, *J* = 12.9 Hz), 6.96–7.54 (m, 15H), 7.72 (s, 2H), 7.83 (s, 1H), 7.94 (s, 1H). HRMS (ESI) calcd for C_58_H_69_N_5_O_2_ [M + H]^2+^, 433.7720; found, 433.7719.

### Fluorescence Spectroscopy and Quantum Yield Measurements

The fluorescence properties of compounds **16**–**20**, **23**, and **28**–**30** were determined using an Edinburgh FS5 spectrofluorometer, equipped with an integrating sphere holder (SC-30) for QY measurements, and a standard cuvette holder (SC5) for excitation and emission spectra. Compounds were dissolved in CHCl_3_ unless otherwise stated. In excitation and emission measurement experiments, the excitation and the emission bandwidth was set to a maximum of 3 nm. The emission spectra were obtained from λ_exc,max_ + 50 nm to 900 nm, with excitation set at the appropriate excitation wavelength. Fluorescence quantum yields were obtained through the “direct excitation” method, comparing the integrals of the scans of the excitation scatter regions and emission scatter region of the blank (solvent alone) and the compound solution with an optical density lower than 0.1. λ_exc_ and λ_em_ were set both at the λ_exc,max_ with the λ_exc_ bandwidth corresponding to 10% of the λ_em_ bandwidth. The calculation was performed using Fluoracle.

### Computational Studies

#### Molecular Docking Simulations

Siramesine, **8**, **17**, **23**, **29**, and **30** were docked on the recently published X-ray structures of the σ_1_ receptor (resolution of 3.12 Å, PDB entry 6DK1(65)) and σ_2_ receptor (resolution of 2.41 Å, PDB entry 7M95(62)). The retrieved PDB files were prepared using the Protein Preparation Wizard tool, available from Schrodinger Suite 2021-2,^66^ for adding missing hydrogen atoms, reconstructing incomplete side chains, assigning favorable protonation states at physiological pH, and performing a force field based minimization of the 3D protein structures. All of the ligands were prepared using the LigPrep tool^67^ for generating all of the possible ionization states and tautomers at pH 7.0 ± 2.0. The obtained files were employed for docking simulations performed by grid-based ligand docking with energetics (GLIDE).^68^ As a first step, siramesine and **8** were subjected to standard docking simulations. Full flexibility was allowed for the ligands while the protein was held fixed. More specifically, the default force field OPLS_2005^69^ and the standard precision (SP) protocol were employed. For each crystal structure, a cubic grid centered on the cognate ligands [i.e., (+)-pentazocine in 6DK1 and Z1241145220 in 7M95] and obtained by setting an edge of 10 Å for the inner box was used. Furthermore, to properly explore the conformational space of the ligands during the simulations, the number of poses per ligand generated in the initial phase of docking was increased from 5000 (default setting) to 50000 and the number of poses per ligand kept for energy minimization was increased from 400 (default setting) to 4000. This protocol was tested by redocking the cognate ligands into their corresponding binding site. Remarkably, both compounds moved back, during the simulations, to the original X-ray positions with a root-mean-square deviations (RMSDs), computed taking into account all of the heavy atoms, of 0.24 Å (6DK1) and 0.42 Å (7M95). These data strongly supported the robustness of the selected docking procedure. Because both siramesine and **8** share a common substructure with the cognate ligands, docking simulations were performed restricting the exploration of the conformational space so that only those poses consistent with the crystallographic coordinates of the shared substructure were generated (tolerance of 4.0 Å). **17**, **23**, **29**, and **30** were subjected to induced fit docking (IFD) simulations to properly take into account putative conformational rearrangements of the protein binding site during molecular recognition.^70^ As recently shown by a co-authored paper,^71^ it is wise to consider such a docking procedure when the compounds to be docked are characterized by a different shape with respect to the cognate ligands. More specifically, a cubic grid centered on the cognate ligand having an inner box of 10 Å × 10 Å × 10 Å irrespective of the considered protein structure and an outer box of 30 Å × 30 Å × 30 Å (28 Å × 28 Å × 28 Å) in 6DK1 (7M95) was employed. Docking simulations were performed using the SP mode and all of the default settings. It is noteworthy that as a consequence of the very high number of rotamers, **17**, **23**, **29**, and **30** are challenging ligands to be docked (too large conformation space to be investigated). For this reason, we decided to restrict the search so that only poses matching those returned by the common substructure of siramesine and **8** were considered for **17** and **23**, respectively (tolerance of 4.0 Å), while **29** and **30** were docked restricting the conformational space to the poses whose maximum common substructure matches (tolerance of 4.0 Å) the pose returned by reference compound **23**.

#### Refinement of Protein–Ligand Complexes

All of the obtained top-scoring docking poses were subjected to a refinement by using the refine protein–ligand complex tool, available from Schrodinger Suite 2021-2.^72^ In particular, the refinement was restricted to all of the atoms within 5 Å of the ligand, using OPLS-4 as the force field, using VSGB as the implicit model of the solvent, and introducing an implicit membrane to simulate the physiological environment of the σ_1_ and σ_2_ receptors.

#### MM-GBSA Calculations

All of the refined complexes were subjected to molecular mechanics/generalized Born surface area calculations,^73^ following an approach recently published.^74^ Notice that during this calculation, no flexibility was allowed for the residues of the binding site; all of the atoms within 5 Å of the ligand were already subjected to a conformational refinement using the refine protein–ligand complex tool.^72^

### Biology

#### Materials

[^3^H]DTG (29 Ci/mmol) and (+)-[^3^H]pentazocine (40 Ci/mmol) were purchased from PerkinElmer Life Sciences (Zavantem, Belgium). Wistar Hannover rats and male Dunkin guinea pigs (250–300 g) were from Envigo. DTG was purchased from Tocris Cookson Ltd., U.K. (+)-Pentazocine, puromycin and TMEM97 MISSION shRNA (SHCLND-NM_006667), and G418 (Geneticin) were obtained from Sigma-Aldrich-RBI s.r.l. (Milan, Italy). Cell culture reagents were purchased from EuroClone (Milan, Italy). FuGENE HD Transfection Reagent was purchased from Promega (Milan, Italy). Opti-MEM was obtained from Life Technologies Italia (Monza, Italy).

#### σ Receptor Radioligand Binding Assays

All of the procedures for the binding assays were previously described. σ_1_ and σ_2_ receptor binding assays were carried out according to the literature.^25^ The specific radioligands and tissue sources were as follows: (a) σ_1_ receptor, (+)-[^3^H]pentazocine, guinea pig brain membranes without cerebellum (*K*_d_ = 2.82 nM; *B*_max_ = 500 fmol/mg of protein); and (b) σ_2_ receptor, [^3^H]DTG in the presence of 1 μM (+)-pentazocine to mask σ_1_ receptors, rat liver membranes (*K*_d_ = 16.52 nM; *B*_max_ = 1200 fmol/mg of protein). For the σ_2_ receptor binding assay, 10 mg of rat liver membranes, 2 nM [^3^H]DTG, 10 μM DTG (to determine nonspecific binding) or test compounds, and 1 μM (+)-pentazocine (to mask σ_1_ receptors) were equilibrated in a final volume of 500 μL [50 mM TRIS (pH 8.0)] for 120 min at 25 °C. Incubations were stopped by addition of 1 mL of ice-cold buffer [50 mM TRIS (pH 8.0)], and then the suspension was filtered through GF/B presoaked in 0.3% polyethylenimine (PEI) for at least 60 min prior to use. The filters were washed twice with 1 mL of ice-cold buffer. For the σ_1_ receptor binding assay, 15 mg of guinea pig brain membranes, 1.5 nM (+)-[^3^H]pentazocine, and 10 μM (+)-pentazocine (to determine nonspecific binding) or test compounds were equilibrated in a final volume of 500 μL [50 mM TRIS (pH 8.0)] for 120 min at 25 °C. Incubations were stopped by addition of 1 mL of ice-cold buffer [50 mM TRIS (pH 8.0)], and then the suspension was filtered through GF/B presoaked in 0.5% polyethylenimine (PEI) for at least 60 min prior to use. The filters were washed twice with 1 mL of ice-cold buffer. The following compounds were used to define the specific binding reported in parentheses: (a) (+)-pentazocine (73–87%) for σ_1_ receptors and (b) DTG (85–96%) for σ_2_ receptors. Concentrations required to inhibit 50% of radioligand specific binding (IC_50_) were determined by using six to nine different concentrations of the drug studied in two or three experiments with samples in duplicate. Scatchard parameters (*K*_d_ and *B*_max_) and apparent inhibition constants (*K*_i_) were determined by nonlinear curve fitting using GraphPad Prism (version 5.0).^64^

#### Cell Culture

The MCF7 human breast adenocarcinoma cell line was purchased from ICLC (Genoa, Italy). The MCF7KO cell line was produced in our laboratory as reported below. The MCF7σ1 cell line was produced in our laboratory starting from MCF7.^63^ MCF7, MCF7σ1, and MCF7KO cells were cultured in DMEM high glucose with 10% FBS, penicillin (100 μg/mL), and streptomycin (100 μg/mL). G418 (0.4 mg/mL) was added in media of MCF7σ1 cells, while 2 μg/mL puromycin was added in media of MCF7KO cells. Cells were maintained in a humidified incubator at 37 °C with 5% CO_2_.

#### MCF7KO Transfection with sh_RNA Targeting TMEM97

The procedure for stably developing MCF7 with a reduced σ_2_ receptor, identified as TMEM97 (MCF7KO cell line), was carried out according to the procedure described in ref (25) with minor modifications. MCF7 cells were plated at a density of 3 × 10^6^ cells/well in 10 mL of growth medium in 100 mm Petri dishes and incubated at 37 °C overnight. Cells were transfected with 17 μg of the pLKO.1 vector containing sh_RNA targeting TMEM97, as per the standard protocol using FuGENE HD Transfection Reagent in Opti-MEM medium without serum. Vector-silencing cells were selected using puromycin. After transfection, cells were placed in normal DMEM growth medium. After 1 day, cells were detached with trypsin/EDTA, replated into DMEM growth medium containing puromycin (2 μg/mL), and cultured for 25 days. Surviving cell clones were picked out and propagated separately in 60 mm Petri dishes in the same medium, with 2 μg/mL puromycin. To suppress reversion of the phenotype, all subsequent cell culturing was carried out in DMEM growth medium as described above, supplemented with 2 μg/mL puromycin.

#### Saturation Binding Assay with [^3^H]DTG

The saturation experiments were carried out as described by Abate et al.^25^ with minor modifications in human MCF7 and MCF7KO adenocarcinoma breast cancer cell membranes. σ_2_ receptors were radiolabeled using [^3^H]DTG concentrations of 0.5–60 nM. Samples containing 200 μg of membrane protein, a radioligand, 10 μM DTG (to determine nonspecific binding), and 1 μM (+)-pentazocine (to mask σ_1_ receptors) were equilibrated in a final volume of 500 μL [50 mM TRIS (pH 8.0)] for 120 min at 25 °C. Incubations were stopped by addition of 1 mL of ice-cold buffer [50 mM TRIS (pH 7.4)], and then the suspension was filtered through GF/C presoaked in 0.5% polyethylenimine (PEI) for at least 30 min prior to use. The filters were washed twice with 1 mL of ice-cold buffer. Scatchard parameters (*K*_d_ and *B*_max_) were determined by nonlinear curve fitting using GraphPad Prism (version 5.0).^64^

#### Flow Cytometry Studies

MCF7 and MCF7KO cells (2 × 10^5^ cells/well) were seeded in a 12-well plate and allowed to recover for 24 h at 37 °C. The next day, the cells were incubated with 10 μM (+)-pentazocine (to mask the σ_1_ receptor) for 60 min at 37 °C, followed by treatment with 10 nM, 30 nM, 100 nM, 1 μM, and 10 μM fluorescent ligand (**19** and **29**) and 1 μM **3** for 45 min at 37 °C. Subsequently, the cells were harvested by trypsinization and resuspended in 250 μL of FACS-PBS. The fluorescence intensity was measured by flow cytometry using a BD LSRFortessaTM X-20 cell analyzer (Becton Dickinson, Palo Alto, CA). For this, in total 20000 cells per sample were evaluated. The results were analyzed and quantified using BD FACSDivaTM software.

Similarly, 2 × 10^5^ MCF7 cells/well were seeded in a 12-well plate and allowed to recover for 24 h at 37 °C. The next day, the cells were incubated with 10 μM (+)-pentazocine (to mask the σ_1_ receptor) for 60 min at 37 °C, followed by a treatment with 10 nM, 30 nM, 100 nM, 1 μM, and 10 μM fluorescent ligand (**16**, **17**, or **23**) for 45 min at 37 °C. The same procedure and conditions were applied to MCF7σ_1_ cells which were incubated with compound **2**(51) 10 μM (to mask σ_2_ receptor) for 60 min at 37 °C, followed by treatment with 10 nM, 30 nM, 100 nM, 1 μM, and 10 μM fluorescent ligand **19**. To calculate the *K*_d_ value, MCF7 wild type cells were incubated with increasing concentrations (1, 10, 50, and 100 nmol/L and 1, 5, and 10 μmol/L) of fluorescent ligands for 75 min at 37 °C. When indicated, cells were treated with 20 μM DTG or 10 μM compound **2** followed by 10 μM fluorescent ligand. Otherwise, cells were treated with increasing concentrations (1, 10, 50, and 100 nmol/L and 1, 5, and 10 μmol/L) of DTG or compound **2** followed by 100 nmol/L fluorescent compound for 75 min at 37 °C. To mask σ_1_ receptors, (+)-PTZ (10 μmol/L) was co-incubated. To calculate the *K*_i_ values, cells were treated with increasing concentrations (1, 10, and 100 nmol/L and 1 and 10 μmol/L) of DTG or compound **2** for 75 min at 37 °C followed by 100 nmol/L fluorescent ligand for the same time. To mask σ_1_ receptors, (+)-PTZ (10 μmol/L) was co-incubated.

At the end of the incubation periods, cells were washed twice with PBS, detached with 200 mL of Cell Dissociation Solution (Sigma Chemical Co.) for 10 min at 37 °C, centrifuged at 13000*g* for 5 min, and resuspended in 500 mL of PBS. The fluorescence was recorded using a Bio-Guava easyCyte 5 Flow Cytometry System (Millipore, Billerica, MA), with a 530 nm band-pass filter. For each analysis, 50000 events were collected and analyzed with the InCyte software (Millipore).

#### Confocal Microscopy Studies

MCF7 cells (1 × 10^5^ cells/well) were seeded in a 12-well plate and allowed to recover for 24 h at 37 °C. The next day, the cells were incubated with 10 μM (+)-pentazocine or compound **31**(6) as the selective σ_1_ ligand for 2 h at 37 °C. Subsequently, the cells were treated with the indicated concentrations of **19** or **29** for 1 h at 37 °C. Then, the cells were washed once with PBS, harvested by trypsinization, and resuspended in 1 mL of PBS. Then, the cell preparation for confocal microscopy by using Cytospin was carried out as described by Koh et al.,^75^ followed by cell fixation with 4% paraformaldehyde at room temperature for 30 min. For the staining of σ_2_ receptor expression, cells were permeabilized and blocked with 0.5% Triton X-100 and 1% BSA, respectively, in PBS for 30 min at room temperature followed by incubation of the primary TMEM97 antibody (diluted 1:200 in 1% BSA/0.3% Triton-PBS, Novusbio, catalog no. NBP1-30436) overnight at 4 °C in a wet chamber. The next day, the cells were washed three times with PBS and incubated with a 1:500 dilution of a secondary anti-rabbit AlexaFluor488-labeled antibody (Invitrogen, catalog no. A-11034) for 1 h at room temperature in a wet chamber. After being washed twice with PBS, the cells were incubated with a solution containing DAPI (2.5 μg/mL, D9542) for 15 min at room temperature, followed by three washes with PBS as well as ddH_2_O and embedding with Vectashield (Vectashield Antifade Mounting Media, Vector Laboratories, caalog no. H-1000-10). Then, confocal microscopy was performed on a Zeiss LSM 700 instrument (Carl Zeiss AG, Oberkochen, Germany), equipped with 405, 488, 555, and 639 nm solid state laser diodes using a Plan-Apochromat 63×/NA 1.4/oil lens for staining of σ_2_ receptor expression. The pinhole size was set to 1 AU. The samples were illuminated with 405, 555, and 639 nm lasers, and 1024 × 1024 pixel images were acquired using the PMT detector. A line average of 2 was applied to all channels. In total, three pictures per spot were obtained.

#### Live Cell Imaging

MCF7 cells (4 × 10^4^ cells/well) were seeded on IBIDI slides (coated with polymers, Science Services) and incubated at 37 °C with 5% CO_2_ for two nights. Subsequently, the cells were incubated with 10 μM (+)-pentazocin as a selective σ_1_ ligand for 2 h at 37 °C. The next day, the cells were treated with 1 μM **19** or **29** (diluted in phenol red-free RPMI1640 medium with 10% FBS) in a heated chamber warmed to 37 °C with 5% CO_2_. All images were collected with a visitron live cell inverted widefield brightfield and fluorescence automated microscope, equipped with a super plan fluor ELWD 20×/NA 0.6/DIC Ph2 lens, by a Nikon Eclipse Ti instrument (detection system, Visicam CCD color camera) and the perfect focus system for maintenance of focus over time. For time-lapse experiments, images were collected every 5 min for a period of 4 h. Cy-5 fluorescence was excited with the Lumencor SPECTRACOLOR LED (exposure time of 100 ms), by using a 640/30 nm filter. The bright field pictures were acquired with a white light LED with an exposure time of 40 ms. The videos were created and evaluated with the open source software Fiji.

#### Spinning Disk Microscopy

MCF7 cells (2.4 × 10^4^ cells/well) were seeded on IBIDI slides (coated with polymers, Science Services) and incubated at 37 °C with 5% CO_2_ for 48 h. Afterward, ProlongLife (Invitrogen, catalog no. P36974) was added in a 1:100 dilution to the cells and incubated for 1 h. Subsequently, the medium was removed, and the cells were stained with 250 nM MitoTracker-Bodipy FL (Invitrogen, catalog no. P36974) in phenol red-free medium for 30 min at 37 °C with 5% CO_2_. Then, the MitoTracker was removed, and the cells were incubated with 10 μM (+)-pentazocine with or without 20 μM DTG in phenol red-free medium for 75 min, followed by addition of 0.05 μM **19** and **29** for 30 min at 37 °C with 5% CO_2_. In addition, Hoechst 33342 (0.05 μM) was added 15 min prior to measurement to counterstain for nuclei. Finally, z-stacks of the living cells were performed using an Olympus IXplore SpinSR spinning disk confocal microscope. In total, three pictures per spot were obtained. The Cy-5 intensities of the ligands based on the localization of the GFP- signal of the mitochondria were calculated by the commercially available software cellSens from Olympus. Statistical analysis was performed using GraphPad Prism (version 8).

## Supporting Information Available

Flow cytometry curves in MCF7 and MCF7KO cells with **1** and **3** and flow cytometry curves in MCF7σ_1_ cells with **19**, displacement radioligand binding curves of new fluorescent compounds, σ_2_ flow cytometry saturation binding assay of compounds **19** and **29** in MCF7 cells using DTG or compound **2** as the nonfluorescent σ_2_ reference ligand, σ_2_ flow cytometry binding assay with compounds **19** and **29** in MCF7 cells to determine *K*_i_ values of reference ligands DTG and **2**, flow cytometry curves in MCF7σ_1_ with **19**, representative confocal microscopy images in living cells with **19** and **29** at lower concentrations, RP-HPLC analysis of the final fluorescent ligands, RP-HPLC degradation study in buffer of compounds **19** and **29**, and ^1^H NMR spectra of key intermediates and final representative compounds (PDF)
Sigma-2 receptor PDB (ZIP)
Sigma-1 receptor PDB (ZIP)
Molecular formula strings (CSV)

## Abbreviations Used

4-DMAP: 4-(dimethylamino)phthalimide
AD: Alzheimer’s disease
ALS: amyotrophic lateral sclerosis
BDP-TR: Bodipy-TR
BH_3_·DMS: borane dimethyl sulfide complex
BSA: bovine serum albumin
CDI: 1,1′-carbonyldiimidazole
CNS: central nervous system
Cy-5: cyanine 5
Cy-7: cyanine 7
D: aspartic acid
DAPI: 4′,6-diamidino-2-phenylindole
ddH_2_O: doubly distilled water
DIPEA: *N,N*-diisopropylethylamine
DMEM: Dulbecco’s modified Eagle’s medium
DMF: dimethylformamide
DTG: 1,3-di-*o*-tolylguanidine
E: glutamic acid
EDTA: ethylenediaminotetraacetic acid
ER: endoplasmic reticulum
F: phenylalanine
HATU: 1-[bis(dimethylamino)methylene]-1*H*-1,2,3-triazolo[4,5-*b*]pyridinium 3-oxide hexafluorophosphate
HD: Huntington’s disease
IC_50_: half-maximal inhibitory concentration
IFD: induced fit docking
*K*_d_: dissociation constant
*K*_i_: inhibition constant
KO: knockout
L: leucine
M: methionine
MCF7: breast adenocarcinoma cell line
MCF7σ_1_ cells: MCF7 cells overexpressing σ_1_ receptors
MM-GBSA: molecular mechanics with generalized Born and surface area solvation
MW: microwave
NIR: near-infrared
PBS: phosphate-buffered saline
PDB: Protein Data Bank
PEI: polyethylenimine
PFA: paraformaldehyde
PGRMC1: progesterone receptor membrane component 1
PTZ: pentazocine
QDs: quantum dots
RMSD: root-mean-square deviation
RT: room temperature
SAfiR: structure–affinity relationship
SP: standard precision
TBAB: tetrabutylammonium bromide
THF: tetrahydrofuran
TMEM97: transmembrane protein 97
TRIS: tris(hydroxymethyl)aminomethane
V: valine
Y: tyrosine
