# Supplementary material for: Development of Fluorescent 4-[4-(3H-Spiro[isobenzofuran-1,4′-piperidin]-1′-yl)butyl]indolyl Derivatives as High-Affinity Probes to Enable the Study of σ Receptors via Fluorescence-Based Techniques
Source: J Med Chem. 2023 Mar 15;66(6):3798–817. doi: 10.1021/acs.jmedchem.2c01227 (PMC10041534; doi:10.1021/acs.jmedchem.2c01227)
Supplement: Supplementary file 1 — jm2c01227_si_001.pdf [file jm2c01227_si_001.pdf]

Supporting Information

Development of Fluorescent 4-(4-(3H-Spiro[isobenzofuran-1,4'-piperidin]-1'-yl)butyl)indolyl Derivatives as High-affinity Probes to Enable σ Receptors Study in Fluorescence-Based Techniques

Francesca Serena Abatematteo, [a#] Maria Majellaro,[b#] Bianca Montsch,[c] Rubén Prieto-Díaz,[b] Mauro Niso,[a] Marialessandra Contino,[a] Angela Stefanachi,[a] Chiara Riganti,[d] Giuseppe Mangiatordi,[e] Pietro Delre,[e] Petra Heffeter,[c] Eddy Sotelo[b] and Carmen Abate[a,e]*

[a]Dipartimento di Farmacia-Scienze del Farmaco, Via Orabona, 4 79125 BARI, Italy;

[b]Centro Singular Investigación Quimica Biologica e Materiales Moleculares (CIQUS), Departamento de Quimica Orgánica, Facultade de Farmacia, Universidade de Santiago de Compostela, 15782 Santiago de Compostela, Spain

[c]Center for Cancer Research and Comprehensive Cancer Center, Medical University of Vienna, Borschkegasse 8a, 1090 Vienna, Austria

[d]Department of Oncology, via Santena 5/bis, 10126, Torino, Italy

[e]Consiglio Nazionale delle Ricerche (CNR), Istituto di Cristallografia, Via Amendola, 70126, BARI, Italy

Correspondance to: carmen.abate@uniba.it

ASSOCIATED CONTENT

Molecular Formula String Table; Page S2

Displacement radioligand binding curves of new fluorescent compounds; Page S3

Flow cytometry curves in MCF7 and MCF7KO with **1** and **3**; Page S4

σ_2 Flow Cytometry saturation binding assay of compounds **19** (A) and **29** (B) in MCF7 cells using DTG as the non-fluorescent σ_2 reference ligand; Page S5

σ_2 Flow Cytometry saturation binding assay of compounds **19** (A) and **29** (B) in MCF7 cells using compound **2** as the non-fluorescent σ_2 reference ligand; Page S6

σ_2 Flow Cytometry binding assay with compounds **19** (A) and **29** (B) in MCF7 cells to determine K_i values of reference ligands DTG and **2**; Page S7

Flow cytometry curves in MCF7σ_1 with **19**; Page S8

Quantification of **19** and **29** fluorescence with and without DTG; Page S9

Representative confocal microscopy images: incubation with **19** and **29**; Page S10

RP-HPLC analysis of final fluorescent ligands; Page S11

RP-HPLC degradation study in buffer of **19** and **29**; Page S12

Representative ^{1}HNMR spectra of key intermediates and representative final compounds: Pages S13-S23.

Molecular Formula String Table

Compound	SMILE
**16**	O=C1C2=C(C=C(N(C)C)C=C2)C(N1CCCN3C4=CC=CC=C4C(CCCCN5CCC6(OCC7=C6C=CC=C7)CC5)=C3)=O
**17**	O=C1C2=C(C=CC(N(C)C)=C2)C(N1CCCCCCN3C4=CC=CC=C4C(CCCCN5CCC6(OCC7=C6C=CC=C7)CC5)=C3)=O
**18**	O=C(NCCCCCCN1C2=C(C=CC=C2)C(CCCCN3CCC4(C(C=CC=C5)=C5CO4)CC3)=C1)COC6=CC=C(C7=CC=C8N7[B-](F)(F)[N+]9=C(C%10=CC=CS%10)C=CC9=C8)C=C6
**19**	O=C(NCCCCCCN1C2=C(C=CC=C2)C(CCCCN3CCC4(C(C=CC=C5)=C5CO4)CC3)=C1)CCCCCN6/C(C(C)(C)C7=C6C=CC=C7)=C\C=C\C=C\C8=[N+](C)C(C=CC=C9)=C9C8(C)C.[I-]
**20**	O=C(NCCCCCCN1C2=C(C=CC=C2)C(CCCCN3CCC4(C(C=CC=C5)=C5CO4)CC3)=C1)CCCCCN6/C(C(C)(C)C7=C6C=CC=C7)=C\C=C\C=C\C=C\C8=[N+](C)C(C=CC=C9)=C9C8(C)C.[I-]
**23**	O=C1C2=C(C=C(N(C)C)C=C2)C(N1C3=CC=C4C(N(CCCCN5CCC6(C(C=CC=C7)=C7CO6)CC5)C=C4)=C3)=O
**28**	O=C(COC1=CC=C(C(C=C2)=[N+]3C2=CC4=CC=C(C5=CC=CS5)N4[B-]3(F)F)C=C1)NC6=CC=C7C(N(CCCCN8CCC9(C(C=CC=C%10)=C%10CO9)CC8)C=C7)=C6
**29**	O=C(CCCCCN1/C(C(C)(C)C2=C1C=CC=C2)=C/C=C/C=C/C3=[N+](C)C(C=CC=C4)=C4C3(C)C)NC5=CC=C6C(N(CCCCN7CCC8(C(C=CC=C9)=C9CO8)CC7)C=C6)=C5.[I-]
**30**	O=C(CCCCCN1/C(C(C)(C)C2=C1C=CC=C2)=C/C=C/C=C/C=C/C3=[N+](C)C4=C(C=CC=C4)C3(C)C)NC5=CC=C6C(N(CCCCN7CCC8(C(C=CC=C9)=C9CO8)CC7)C=C6)=C5.[I-]

Figure S1. Displacement radioligand binding curves of new fluorescent compounds

σ1

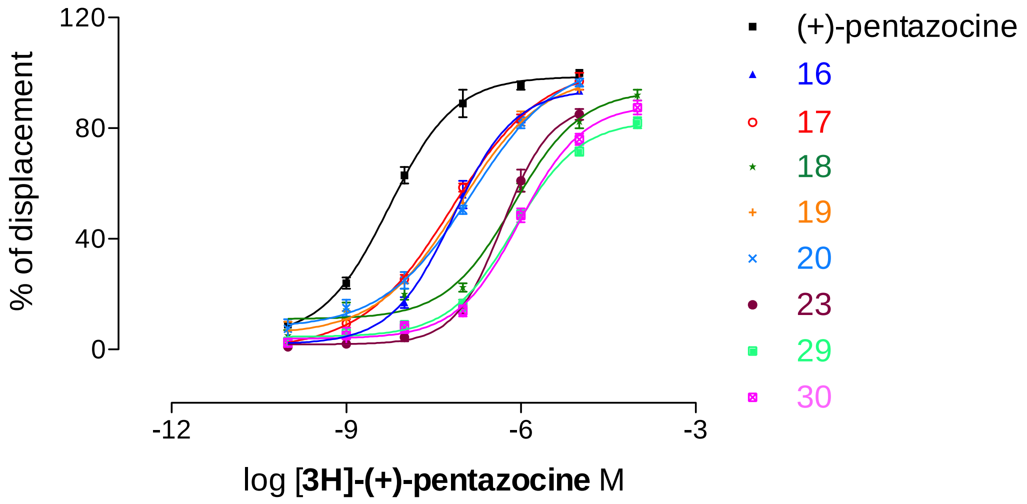

σ2

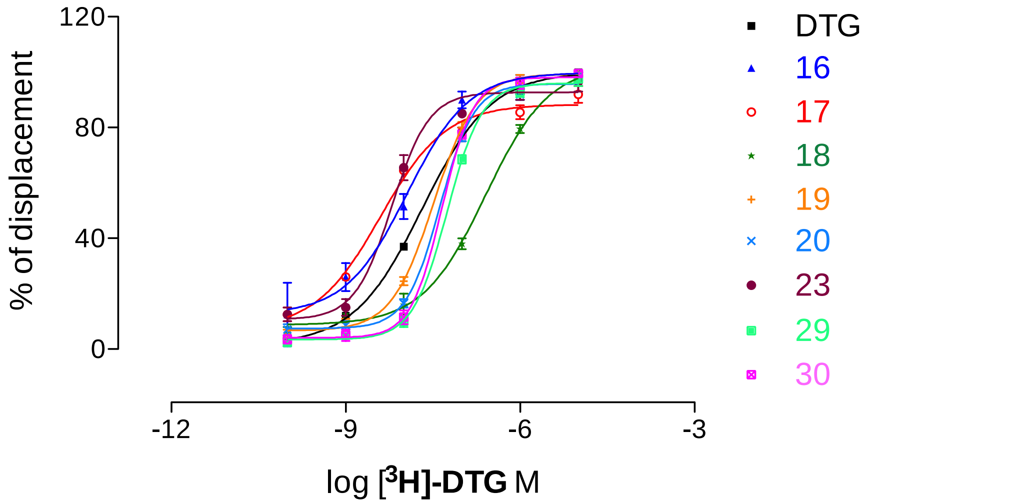

Figure S2. Flow cytometry studies in MCF7 and MCF7 silenced in TMEM97/σ$_2$ (MCF7KO) with reference fluorescent ligands **1** and **3**

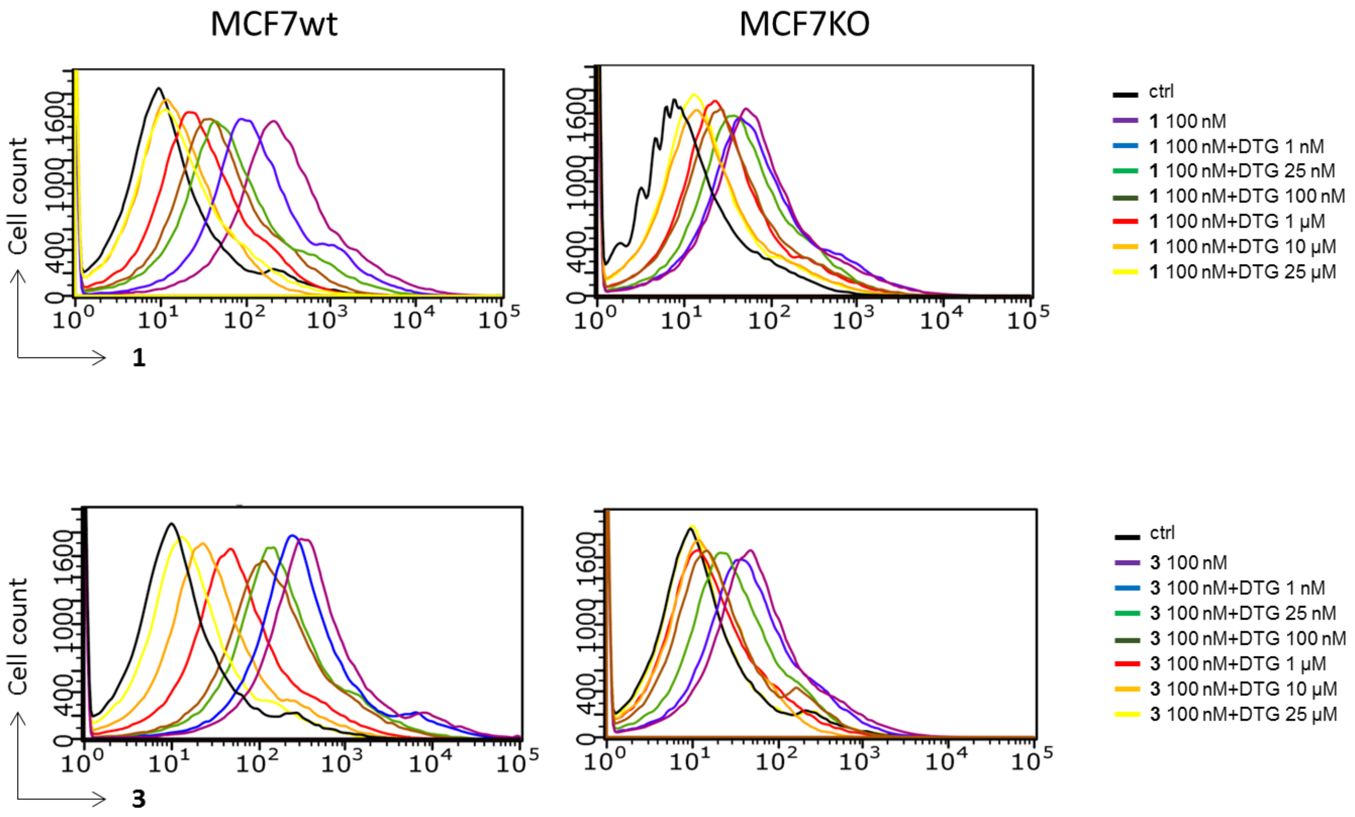

Figure S3. σ_2 Flow cytometry saturation binding assay of compounds **19** (A) and **29** (B) in MCF7 cells using DTG as the non-fluorescent σ_2 reference ligand.

A

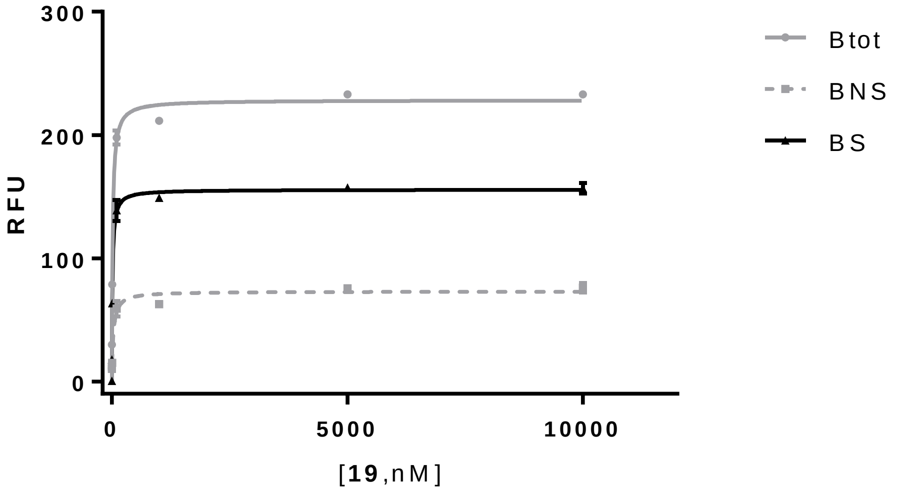

B

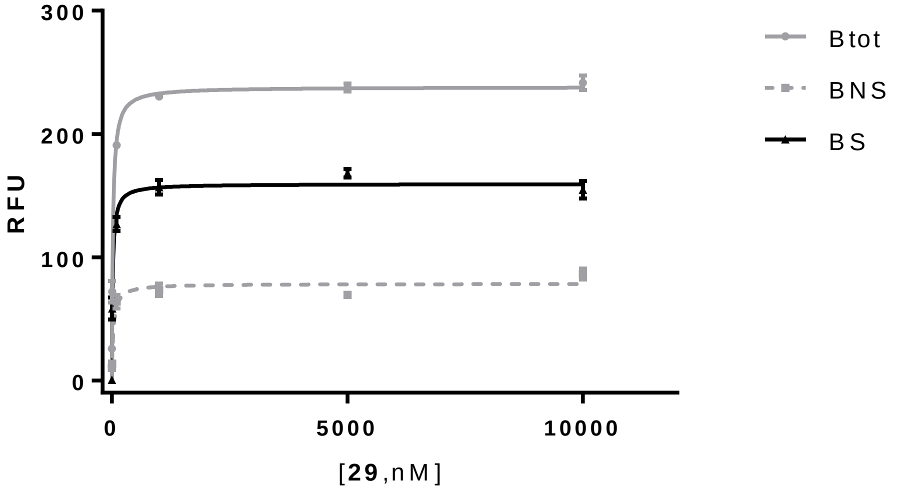

σ_1 subtype is masked by 1 μM (+)-pentazocine. Specific binding data (BS) are represented as RFU (y-axis) and concentration (x-axis). BS on the y-axis are measured by subtracting the non-specific binding BNS (obtained in the presence of the not fluorescent ligand DTG 20 μM) from the total binding Btot (obtained in the presence of the fluorescent ligand alone at each studied concentration). Standard deviation for BNS and Btot and propagated error for BS is reported for each point. The dissociation constant K_d were measured by the nonlinear fitting of the specific binding *vs* fluorescent ligand concentration using Prism software: K_d = 13.59 nM for compound **19**; K_d = 18.66 nM for compound **29**.

Figure S4. σ_2 Flow cytometry saturation binding assay of compounds **19** (A) and **29** (B) in MCF7 cells using compound **2** as the non-fluorescent σ_2 reference ligand.

A

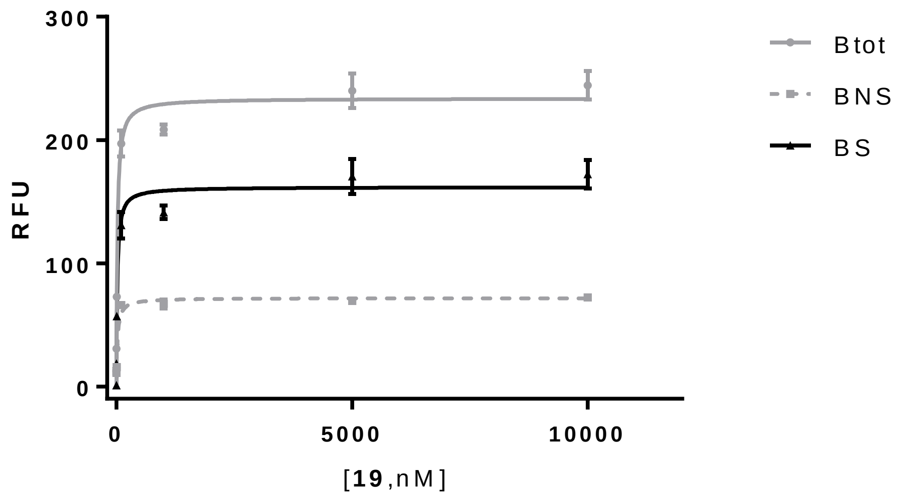

B

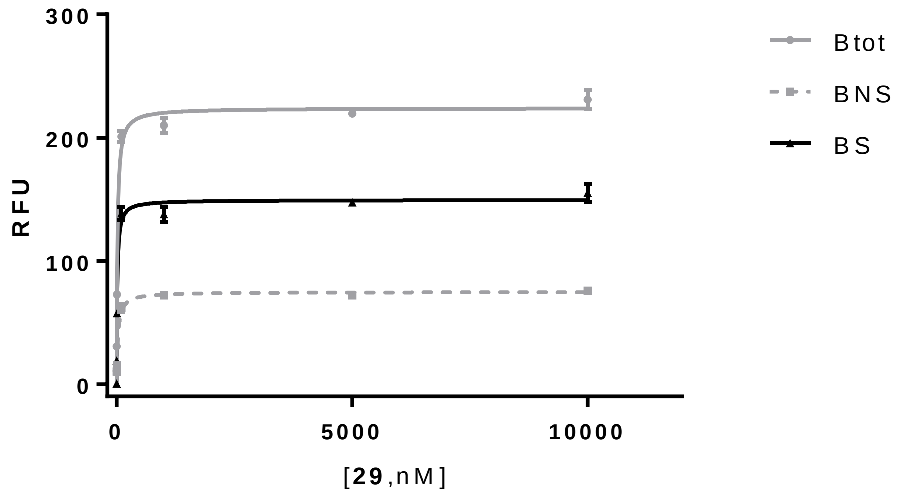

σ_1 subtype is masked by 1 μM (+)-pentazocine. Specific binding data (BS) are represented as RFU (y-axis) and concentration (x-axis). BS on the y-axis are measured by subtracting the non-specific binding BNS (obtained in the presence of the not fluorescent ligand **2** 10 μM) from the total binding Btot (obtained in the presence of the fluorescent ligand alone at each studied concentration). Standard deviation for BNS and Btot and propagated error for BS are reported for each point. The dissociation constant K_d were measured by the nonlinear fitting of the specific binding *vs* fluorescent ligand concentration using Prism software: K_d = 19.32 nM for compound **19**; K_d = 13.82 nM for compound **29**.

Figure S5. σ_2 Flow cytometry binding assay with compounds **19** (A) and **29** (B) in MCF7 cells to determine K_i values of reference ligands DTG and **2**. K_d values of **19** and **29** using DTG were employed to determine K_i values.

A

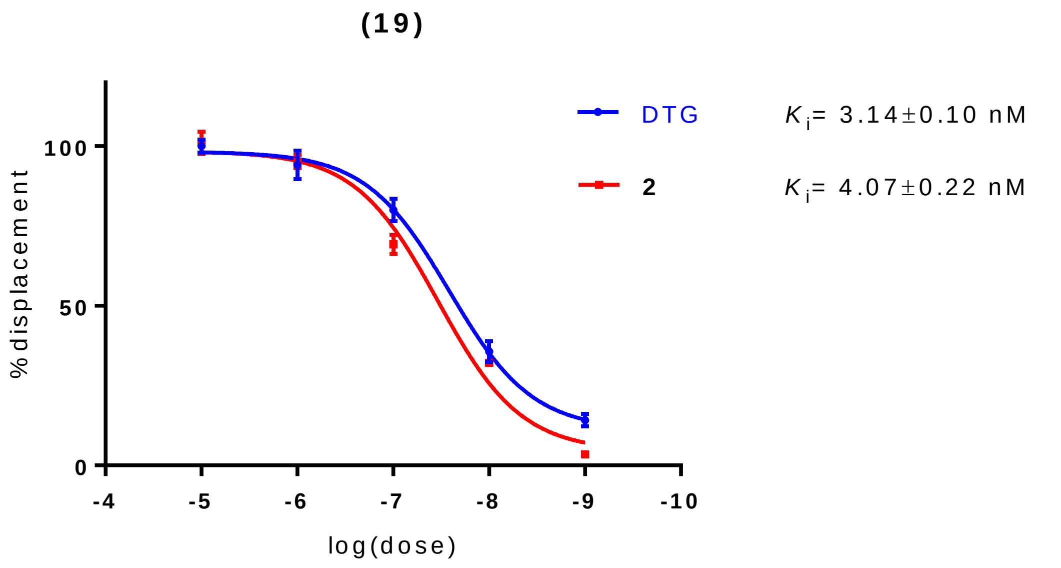

B

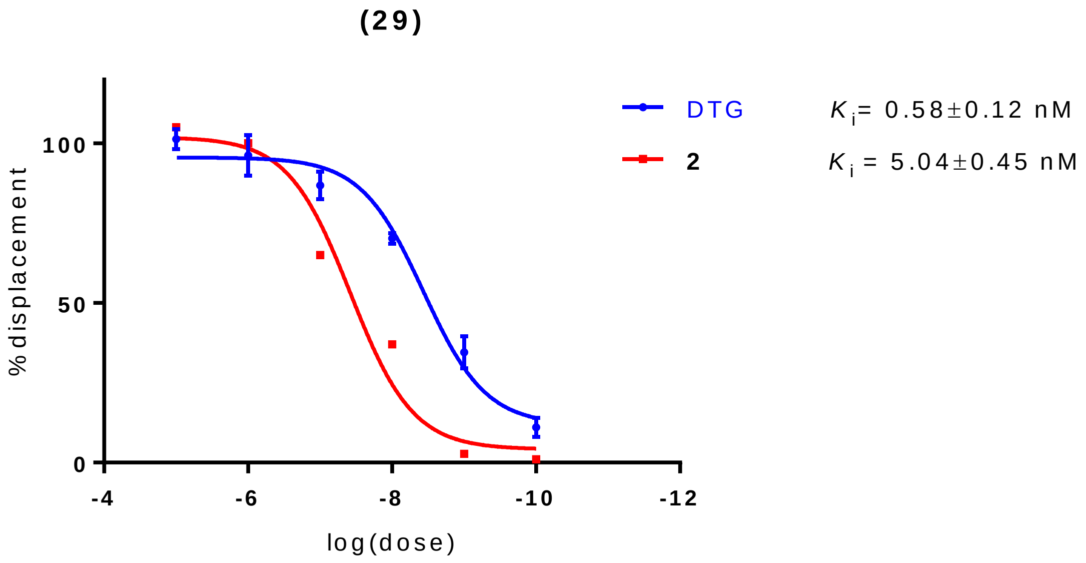

Figure S6. Representative flow cytometry curves obtained with compound **19** in MCF7σ_1. A) Increasing concentrations of **19**, upon masking σ_2 receptors with **2** (10 μM); B) Increasing concentrations of **19**, upon masking σ_2 receptors with **2** (10 μM) and σ_1 receptors with (+)-pentazocine (10 μM).

A B

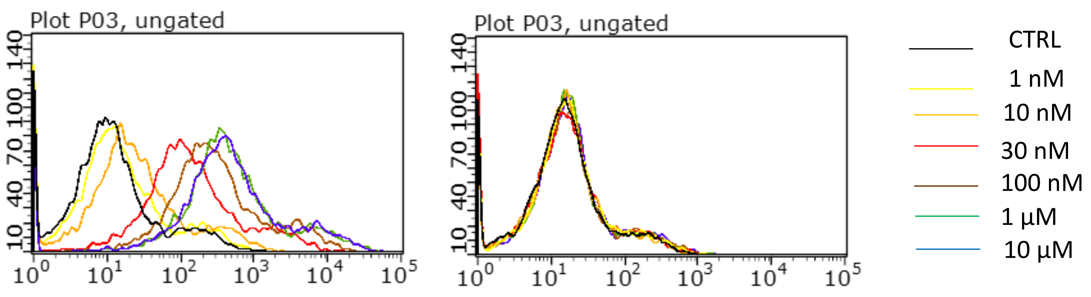

Figure S7. Quantification of the mean gray values of the Cy-5 fluorescence intensities based on the mitochondrial localization of the images (n=3). Statistical significance was calculated by One-way Anova with Tukey´s post analysis (** P < 0.01)

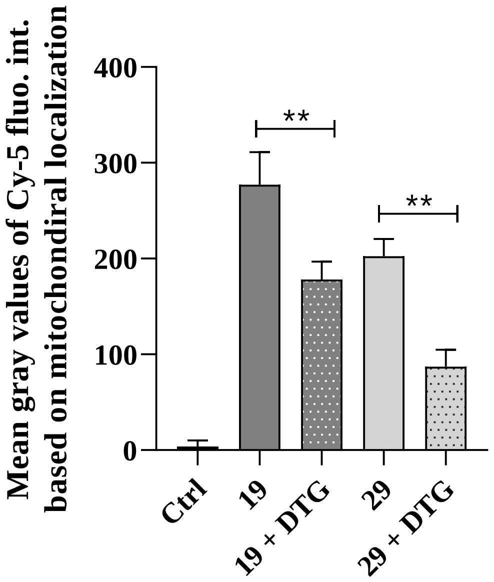

Figure S8. Representative confocal microscopy images show distinct fluorescence intensity of compound **19** and **29** at lower concentrations in MCF7 and MCF7KO cells. Moreover, a reduced signal of the ligands could be observed in MCF7KO compared to MCF7 cells. Cells were pre-incubated with 10 μM of the selective σ_1 receptor agonist **31** for 2 h to mask residual $\sigma1$ receptor expression. This was followed by 1 h incubation with the indicated concentrations of the fluorescent ligands. Then cells were fixed with paraformaldehyde, stained for the nuclei (by DAPI) and analyzed by confocal microscopy. The ligands are shown in red (scale bar: 20 μm).

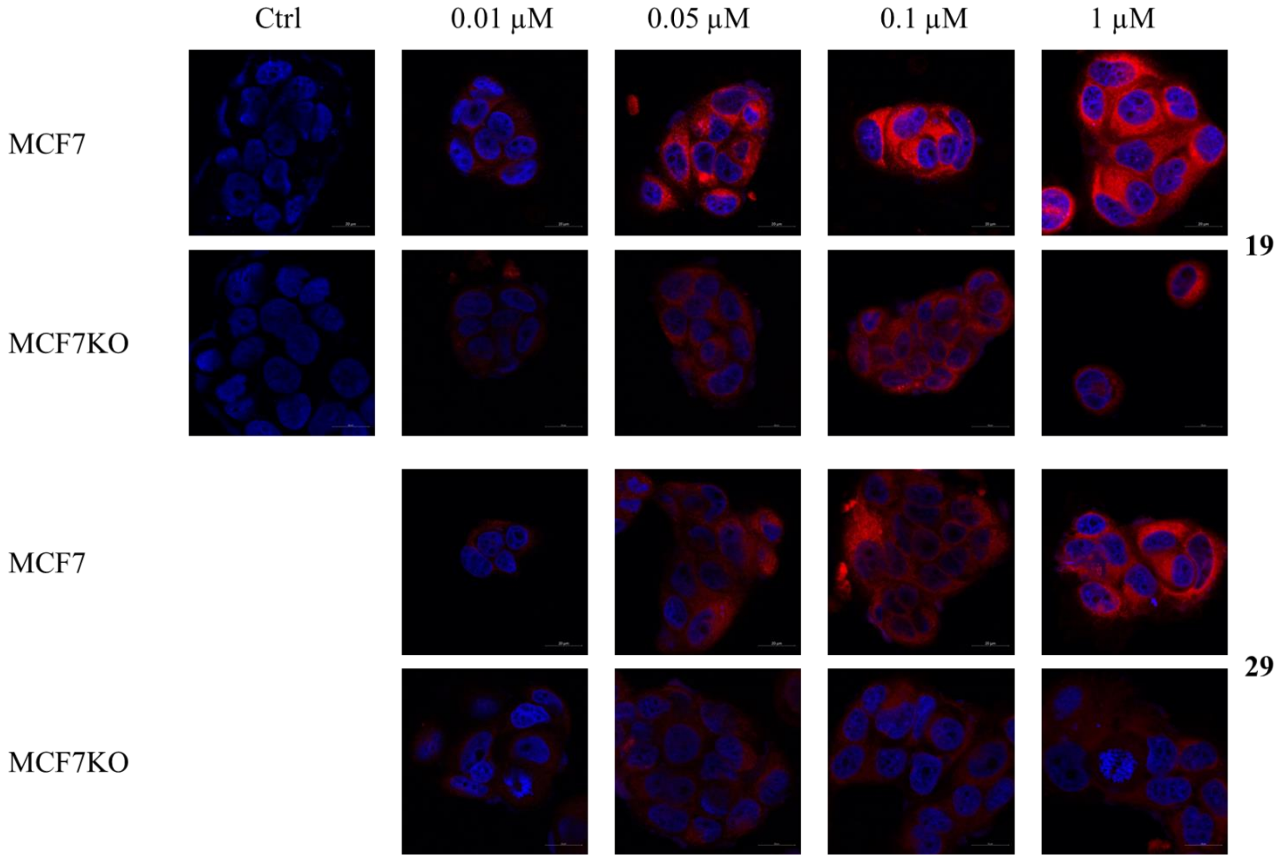

Figure S9. RP-HPLC analysis of final fluorescent ligands.

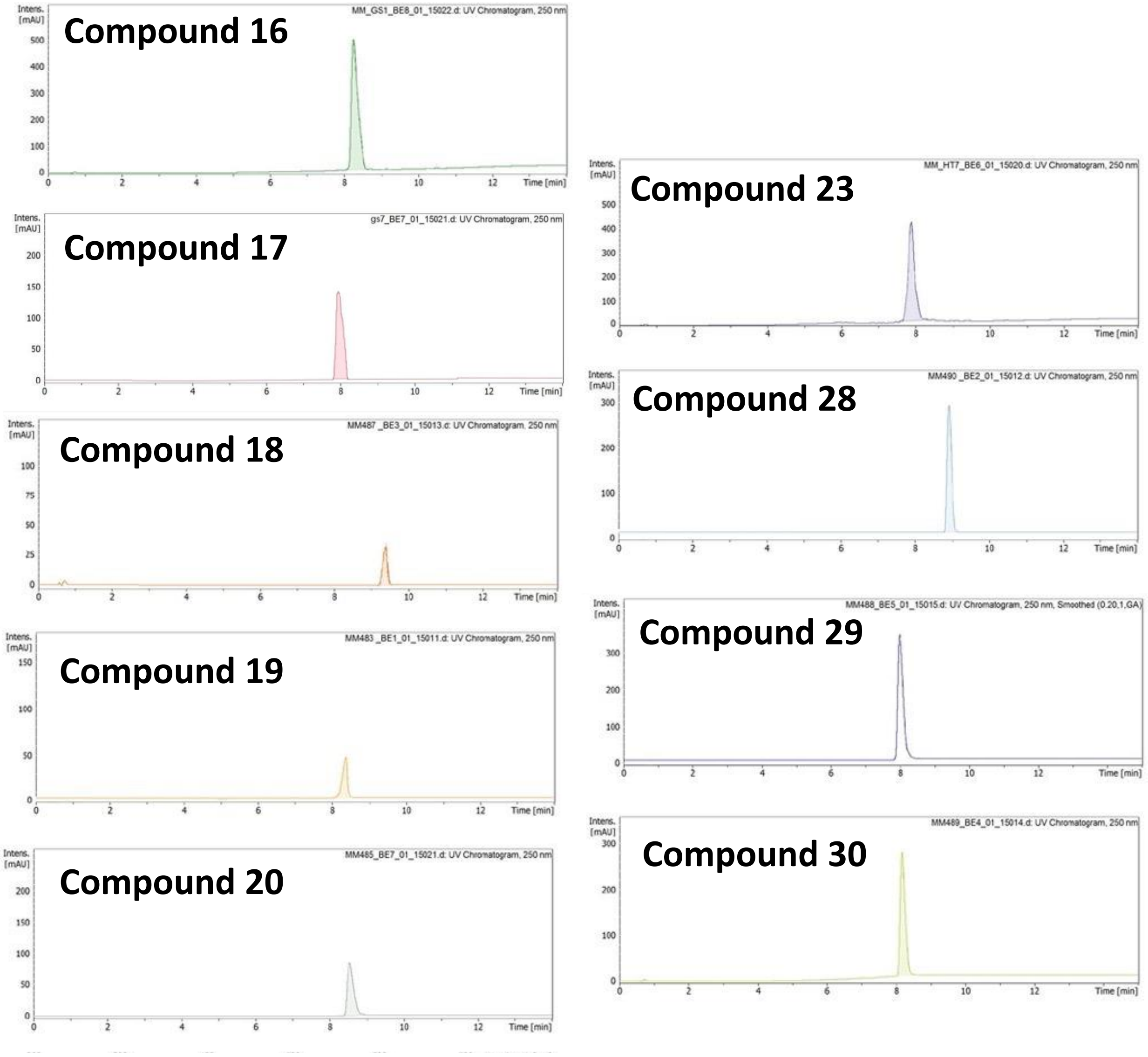

Figure S10. RP-HPLC degradation study in buffer of compounds **19** (A) and **29** (B). Compounds were dissolved in a solution of PBS with 5% of DMSO. Then the solution was divided in 4 vials, one was used to study t0 and the other three were incubated at 37ºC for 2h, 6h and 24 h, respectively.

A

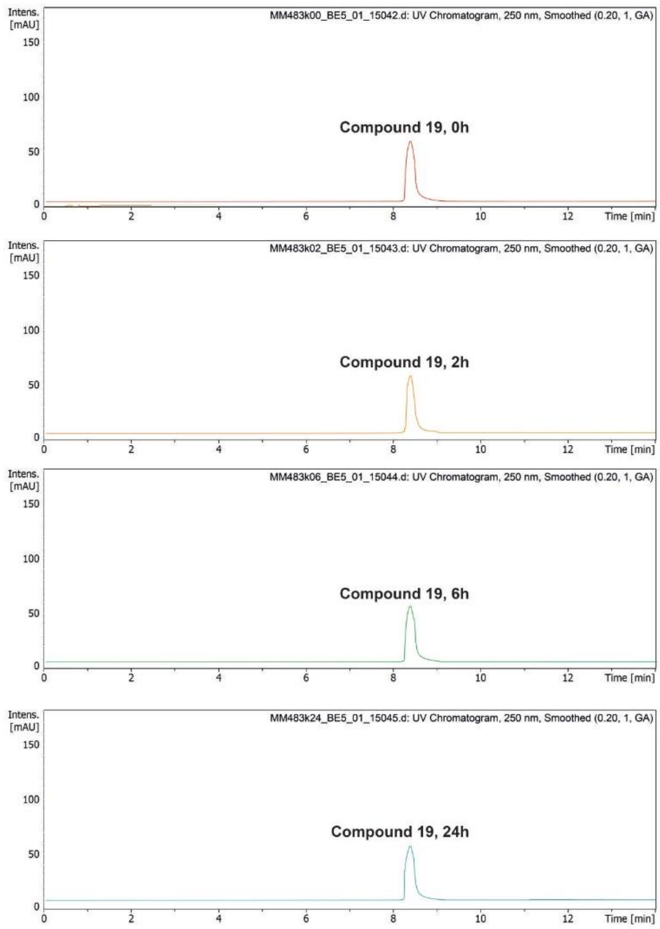

B

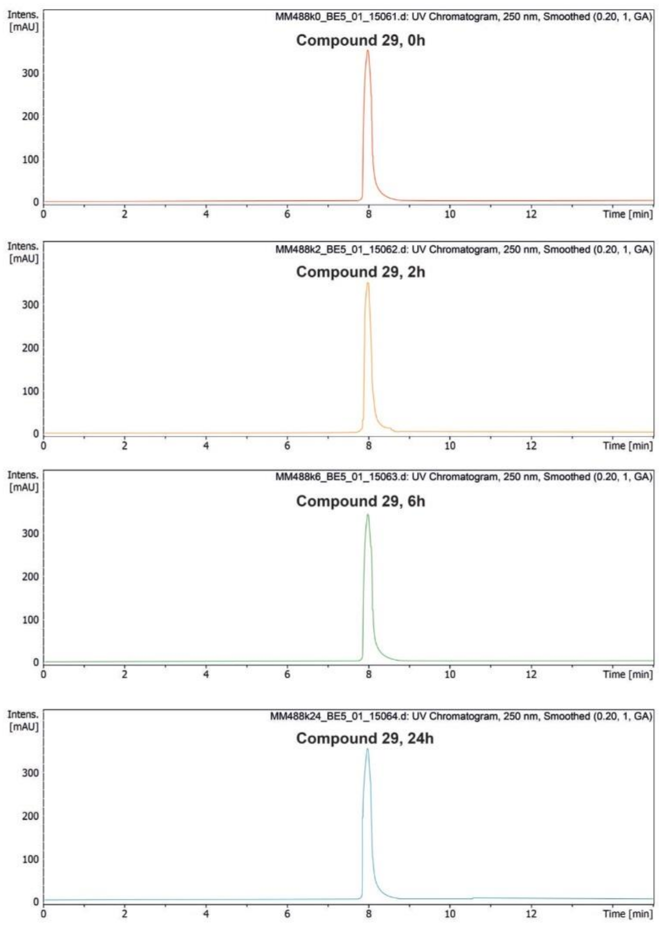

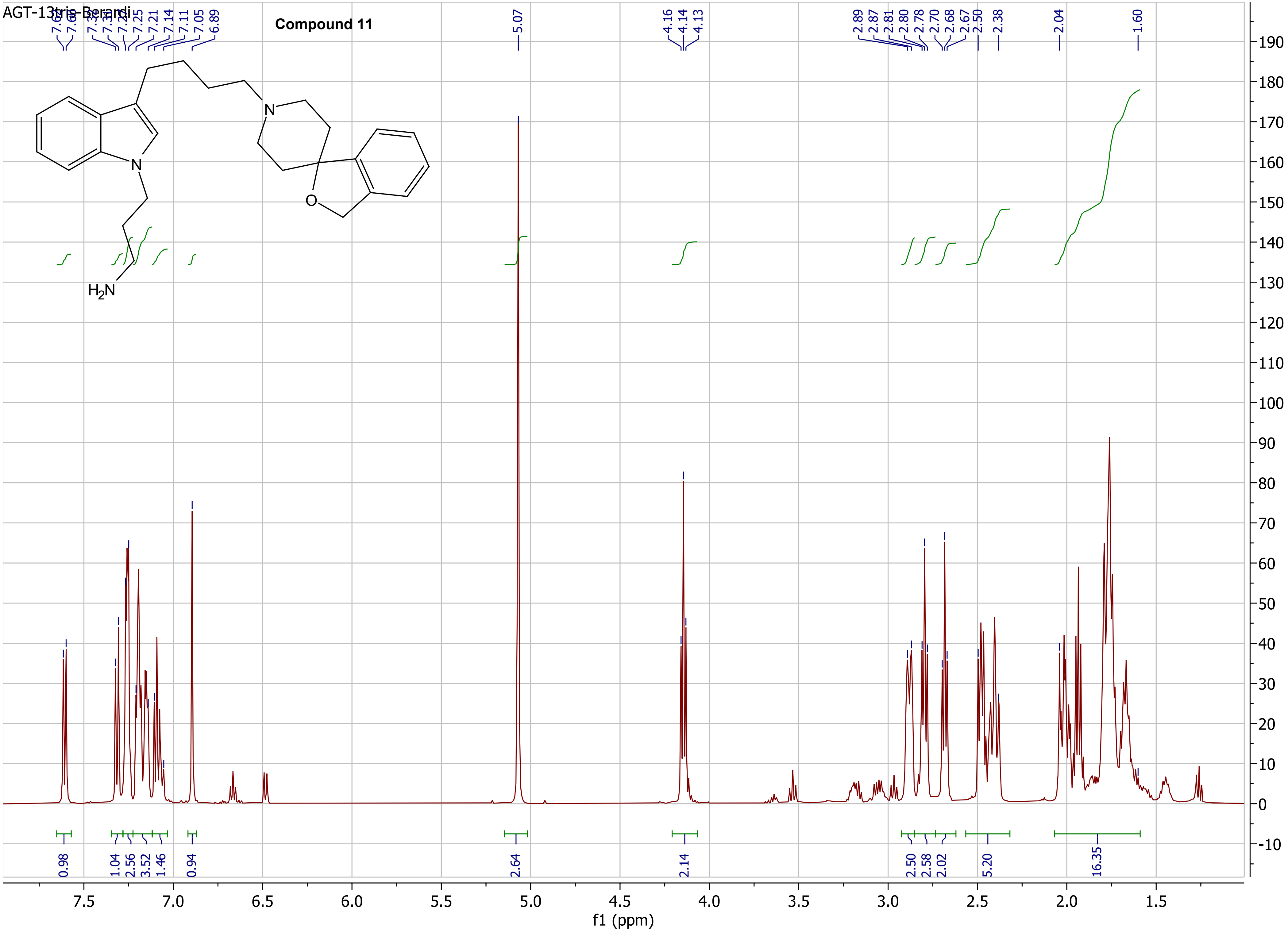

AGT-13tris-Berardi
Compound 11
N
N
O
H_2N
7.25
7.21
7.14
7.11
7.05
6.89
5.07
4.16
4.14
4.13
2.89
2.87
2.81
2.80
2.78
2.70
2.68
2.67
2.50
2.38
2.04
1.60
0.98
1.04
2.56
3.52
1.46
0.94
2.64
2.14
2.50
2.58
2.02
5.20
16.35
f1 (ppm)

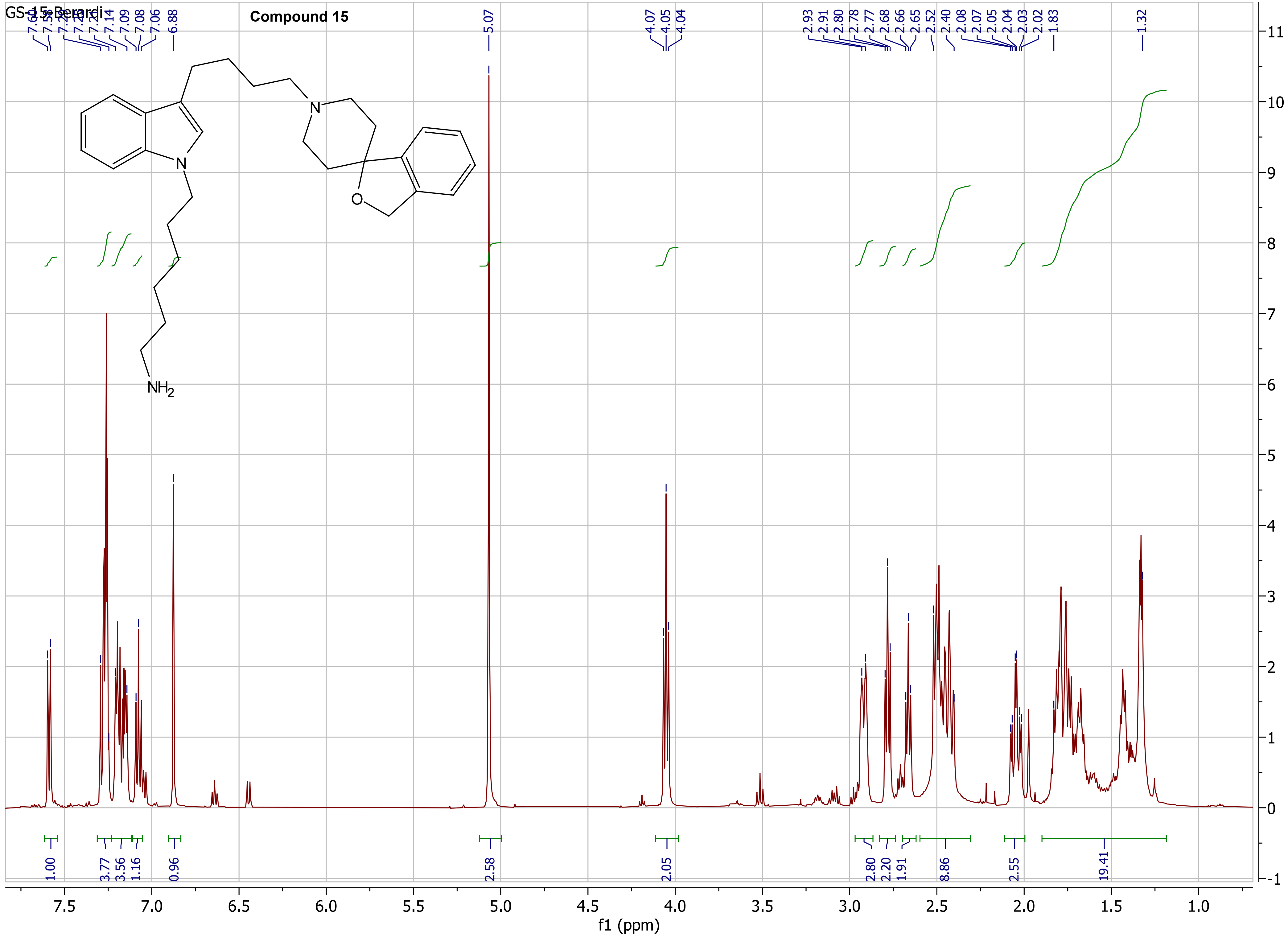
GS-15-Berardi
Compound 15
7.14
7.09
7.08
7.06
6.88
5.07
4.07
4.05
4.04
2.93
2.91
2.80
2.78
2.77
2.68
2.66
2.65
2.52
2.40
2.08
2.07
2.05
2.04
2.03
2.02
1.83
1.32
N
N
O
NH2
1.00
3.77
3.56
1.16
0.96
2.58
2.05
2.80
2.20
1.91
8.86
2.55
19.41
f1 (ppm)

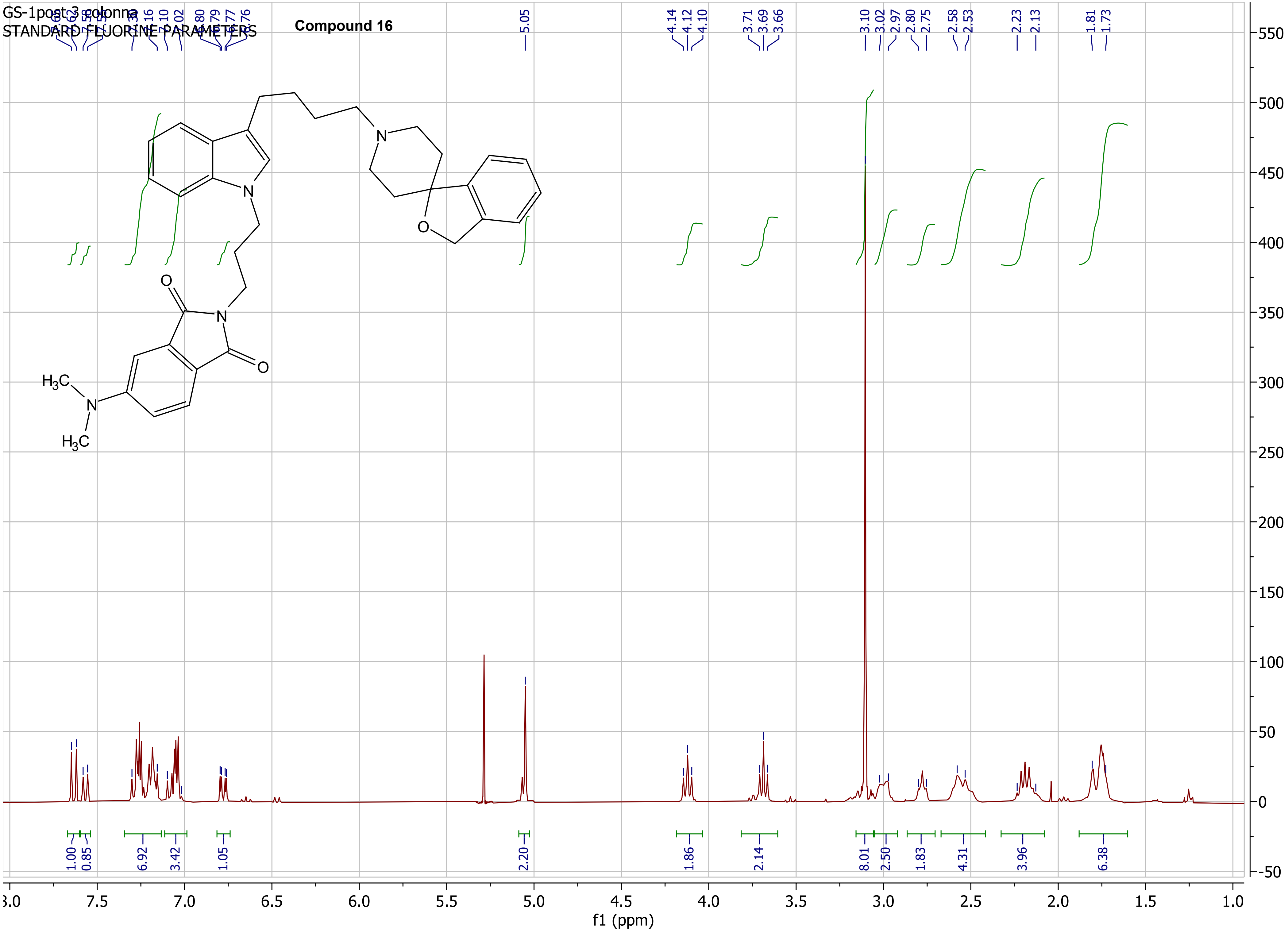
GS-1post 3 colonna
STANDARD FLUORINE PARAMETERS
Compound 16
7.16
7.10
7.02
6.80
6.79
6.77
6.76
5.05
4.14
4.12
4.10
3.71
3.69
3.66
3.10
3.02
2.97
2.80
2.75
2.58
2.53
2.23
2.13
1.81
1.73
1.00
0.85
6.92
3.42
1.05
2.20
1.86
2.14
8.01
2.50
1.83
4.31
3.96
6.38
f1 (ppm)

Compound 23

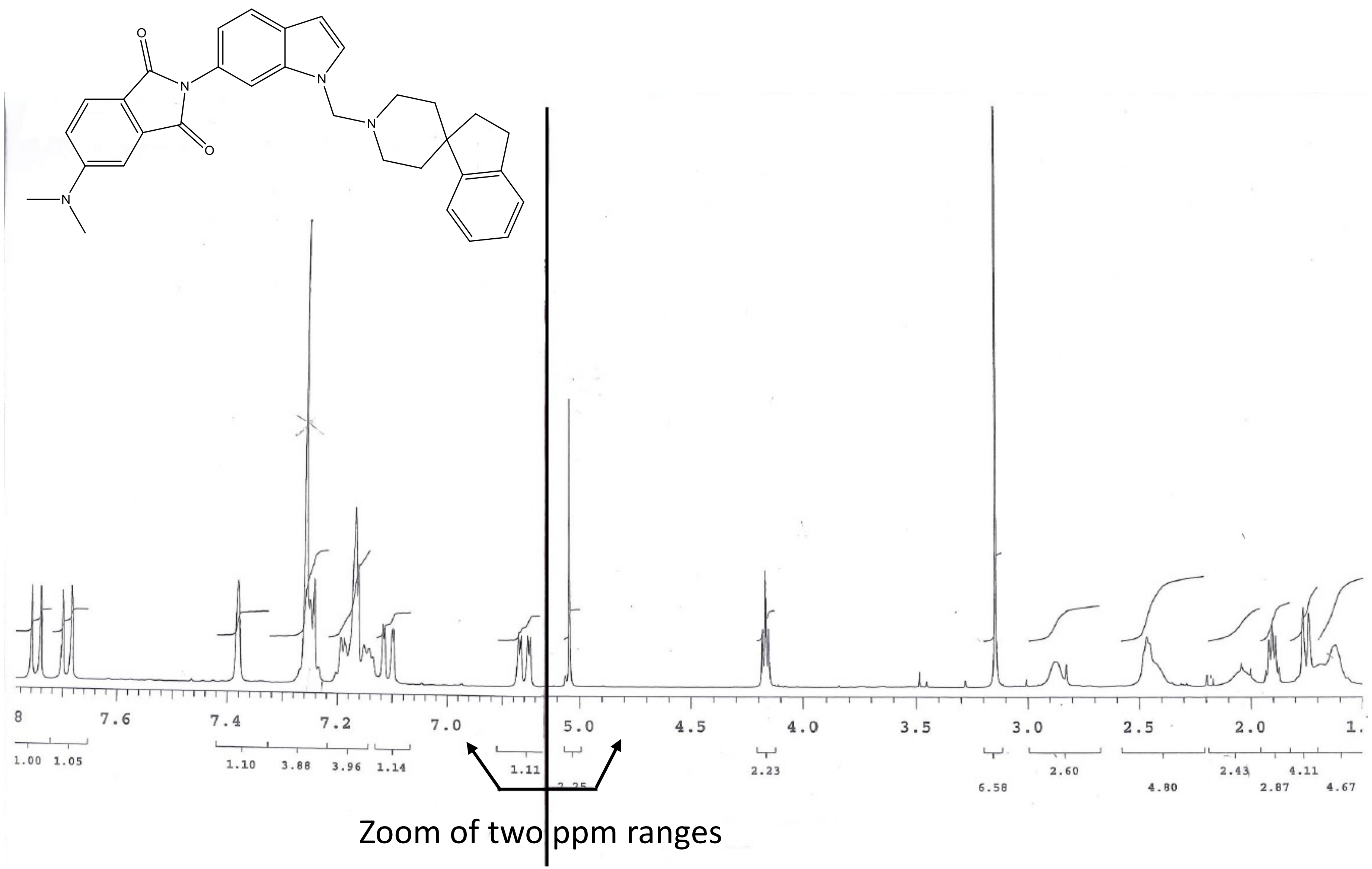

O
N
N
N
O
N
8
7.6
7.4
7.2
7.0
5.0
4.5
4.0
3.5
3.0
2.5
2.0
1.00 1.05
1.10 3.88 3.96 1.14
1.11
2.23
6.58
2.60
4.80
2.43 4.11
2.87 4.67
Zoom of two ppm ranges

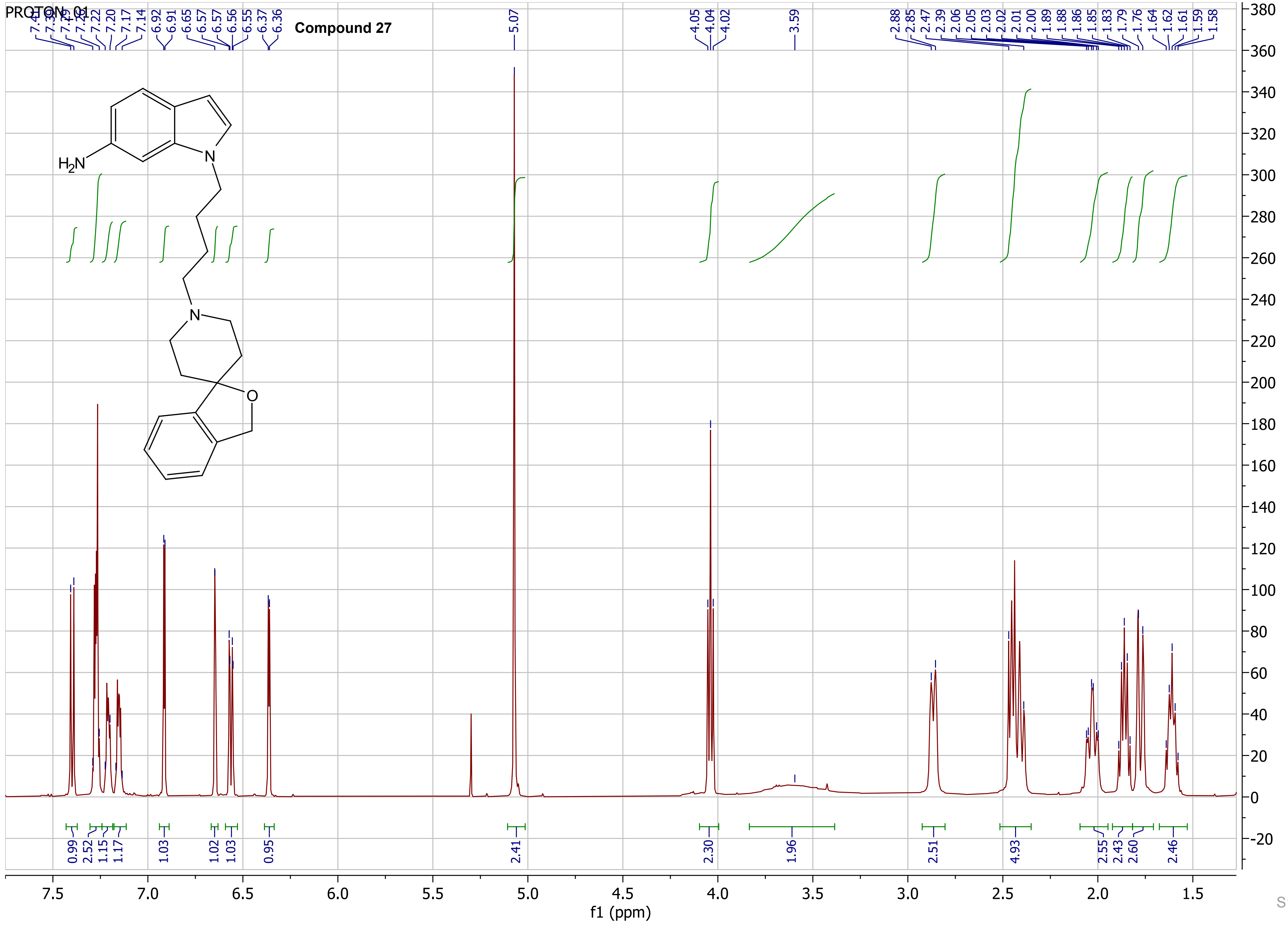
PROTON_01
Compound 27
H2N
N
N
O
7.22 7.20 7.17 7.14 6.92 6.91 6.65 6.57 6.57 6.56 6.55 6.37 6.36
5.07
4.05 4.04 4.02
3.59
2.88 2.85 2.47 2.39 2.06 2.05 2.03 2.02 2.01 2.00 1.89 1.88 1.86 1.85 1.83 1.79 1.76 1.64 1.62 1.61 1.59 1.58
0.99 2.52 1.15 1.17 1.03 1.02 1.03 0.95 2.41 2.30 1.96 2.51 4.93 2.55 2.43 2.60 2.46
f1 (ppm)

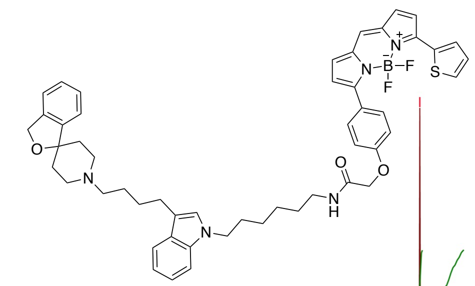

O
N
N
O
N
H
O
N
N
B
F
F
S

18

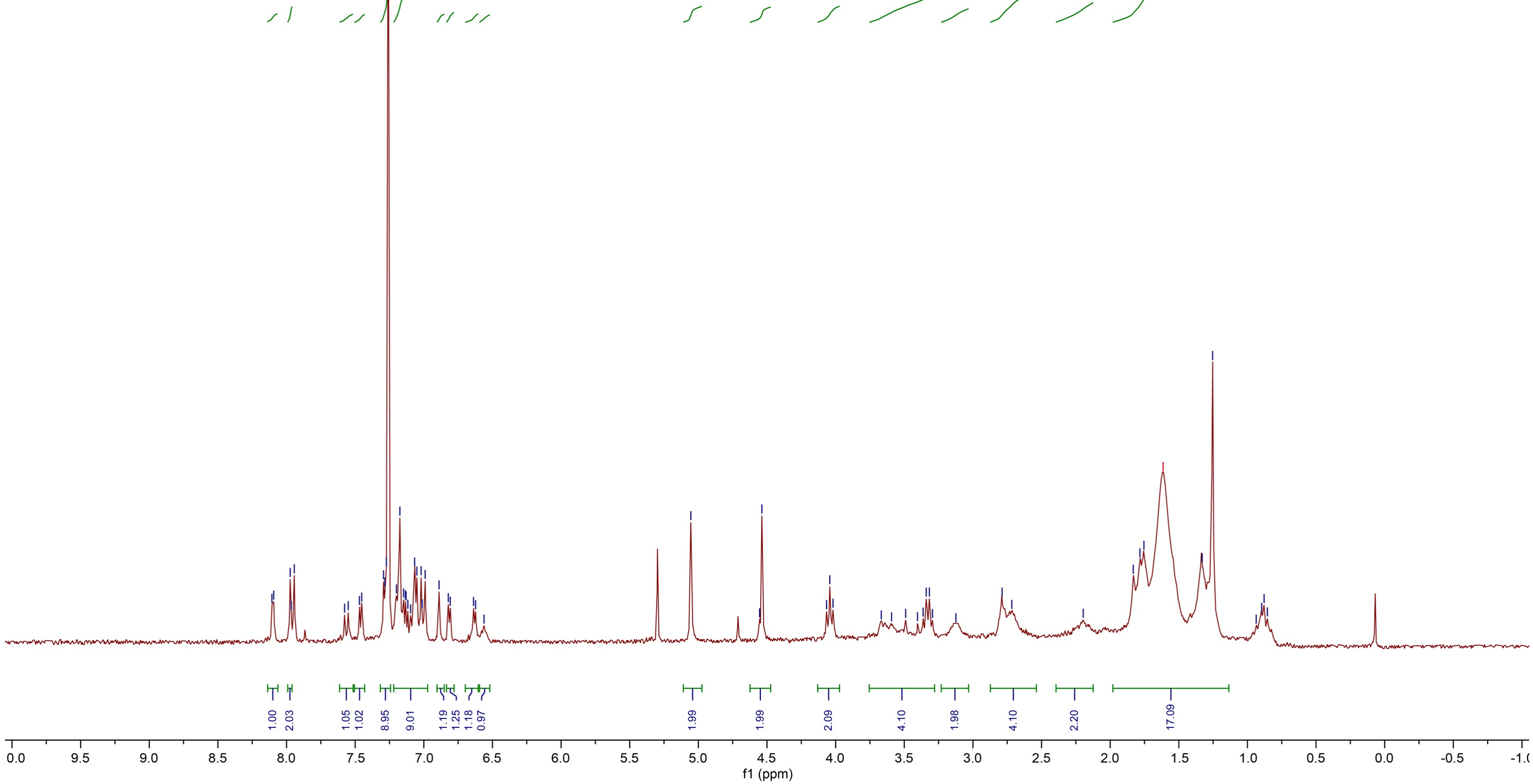

1.00
2.03
1.05
1.02
8.95
9.01
1.19
1.25
1.18
0.97
1.99
1.99
2.09
4.10
1.98
4.10
2.20
17.09
0.0 9.5 9.0 8.5 8.0 7.5 7.0 6.5 6.0 5.5 5.0 4.5 4.0 3.5 3.0 2.5 2.0 1.5 1.0 0.5 0.0 -0.5
f1 (ppm)

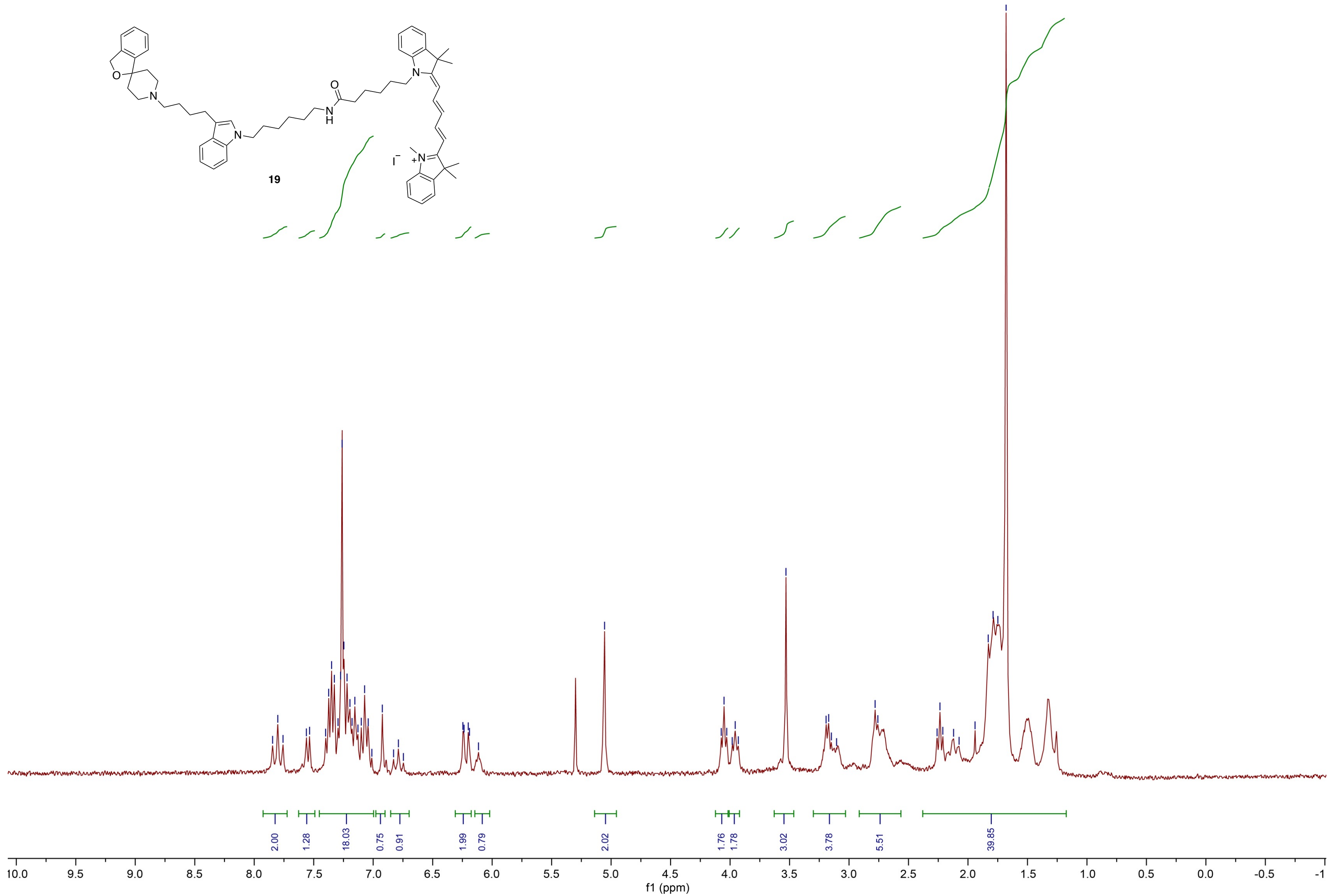
19
2.00
1.28
18.03
0.75
0.91
1.99
0.79
2.02
1.76
1.78
3.02
3.78
5.51
39.85
10.0 9.5 9.0 8.5 8.0 7.5 7.0 6.5 6.0 5.5 5.0 4.5 4.0 3.5 3.0 2.5 2.0 1.5 1.0 0.5 0.0 -0.5 -1
f1 (ppm)

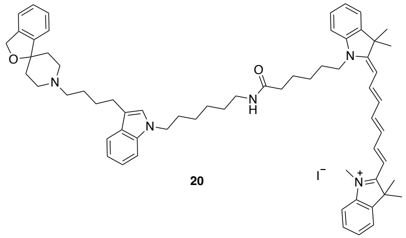
O
N
N
O
N
H
N
N+
I−

20

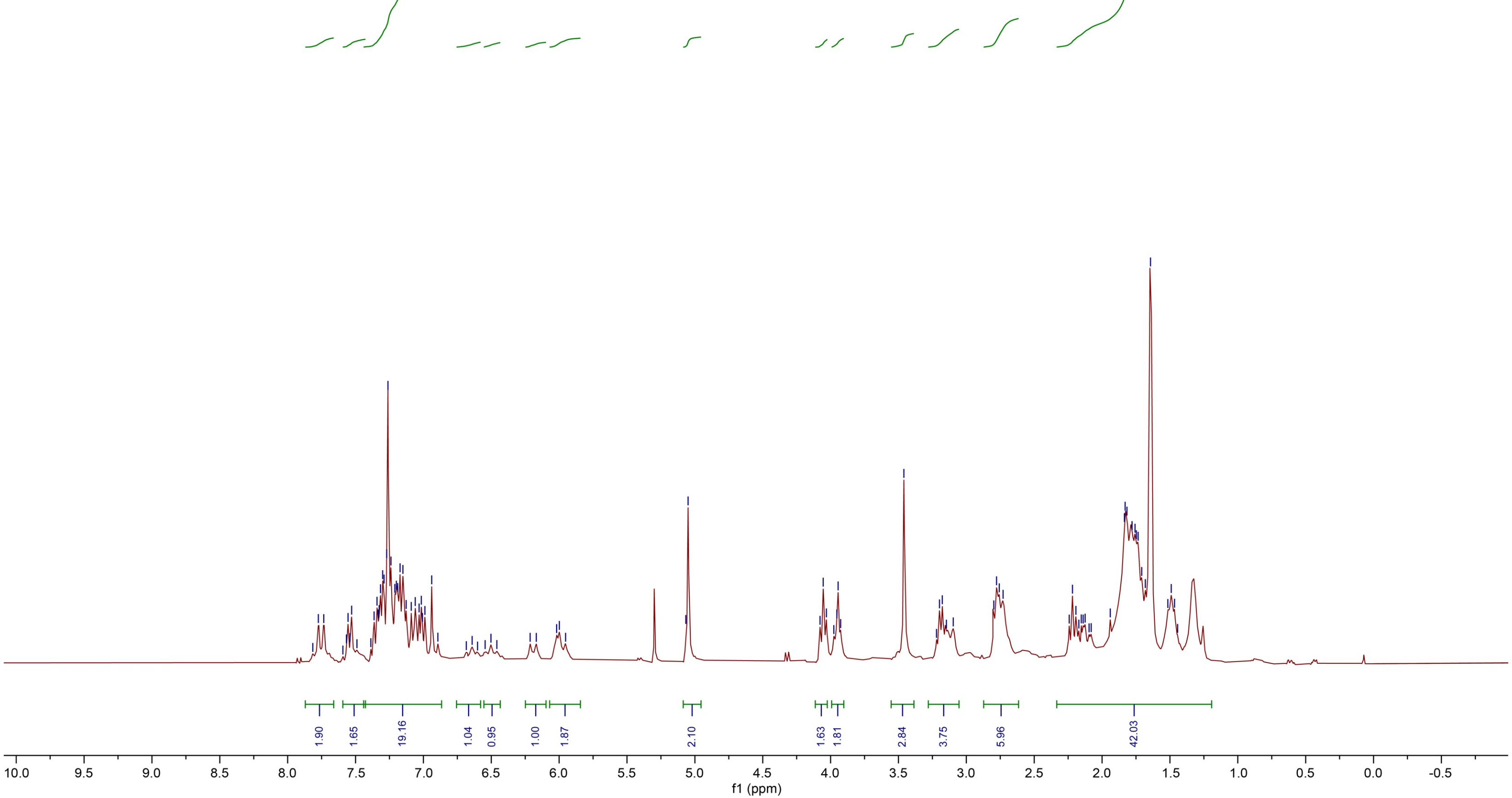
1.90
1.65
19.16
1.04
0.95
1.00
1.87
2.10
1.63
1.81
2.84
3.75
5.96
42.03
10.0 9.5 9.0 8.5 8.0 7.5 7.0 6.5 6.0 5.5 5.0 4.5 4.0 3.5 3.0 2.5 2.0 1.5 1.0 0.5 0.0 -0.5
f1 (ppm)

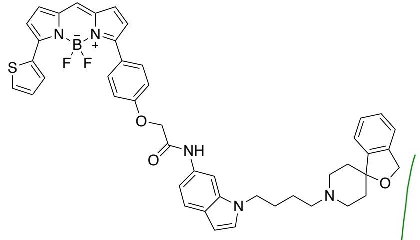

28

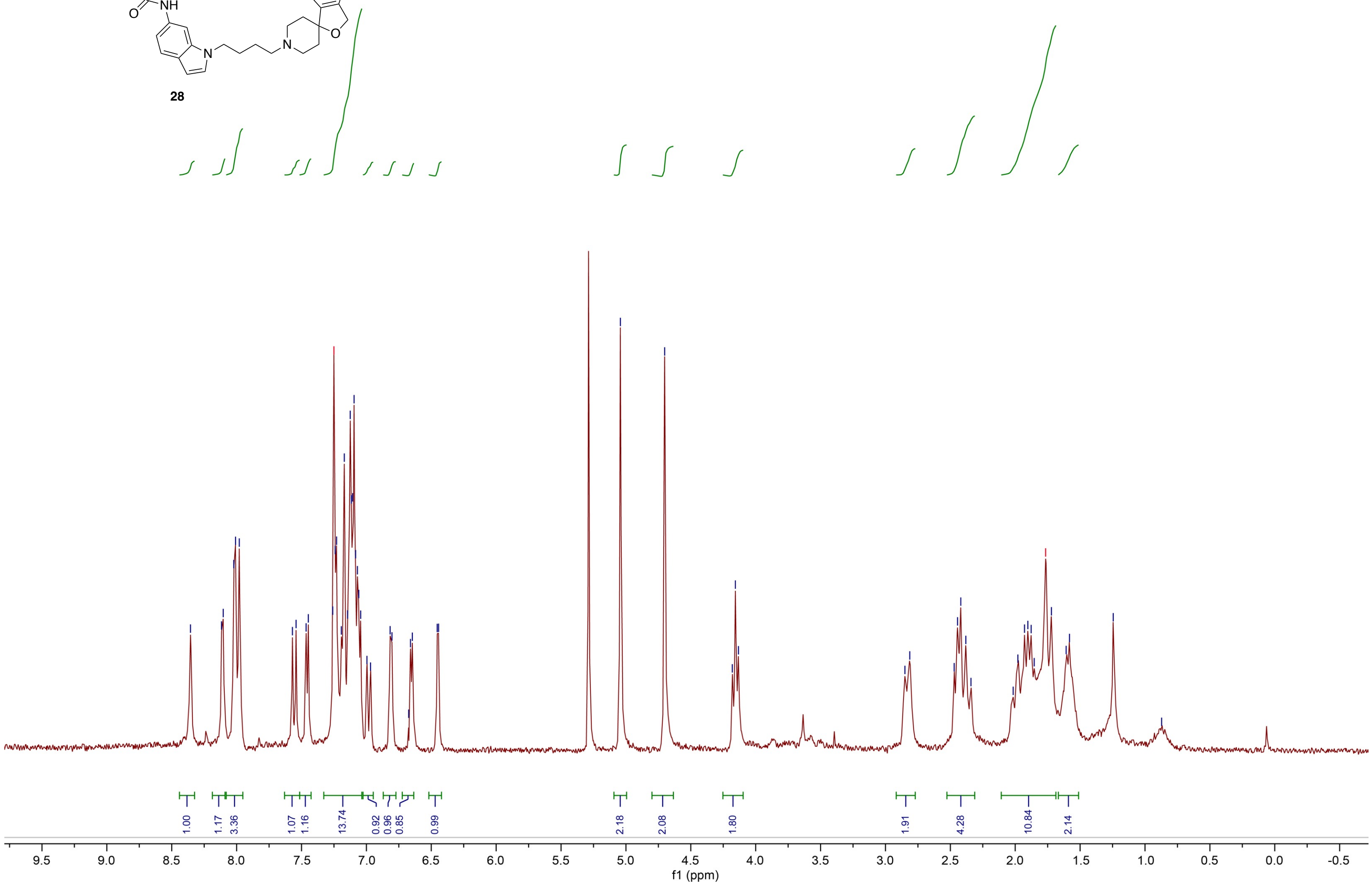

1.00
1.17
3.36
1.07
1.16
13.74
0.92
0.96
0.85
0.99
2.18
2.08
1.80
1.91
4.28
10.84
2.14
9.5
9.0
8.5
8.0
7.5
7.0
6.5
6.0
5.5
5.0
4.5
4.0
3.5
3.0
2.5
2.0
1.5
1.0
0.5
0.0
-0.5
f1 (ppm)

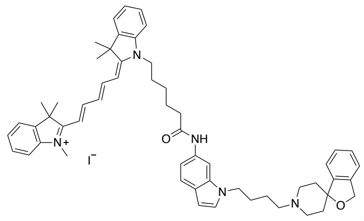

N
N+
I−
O
NH
N
N
O

29

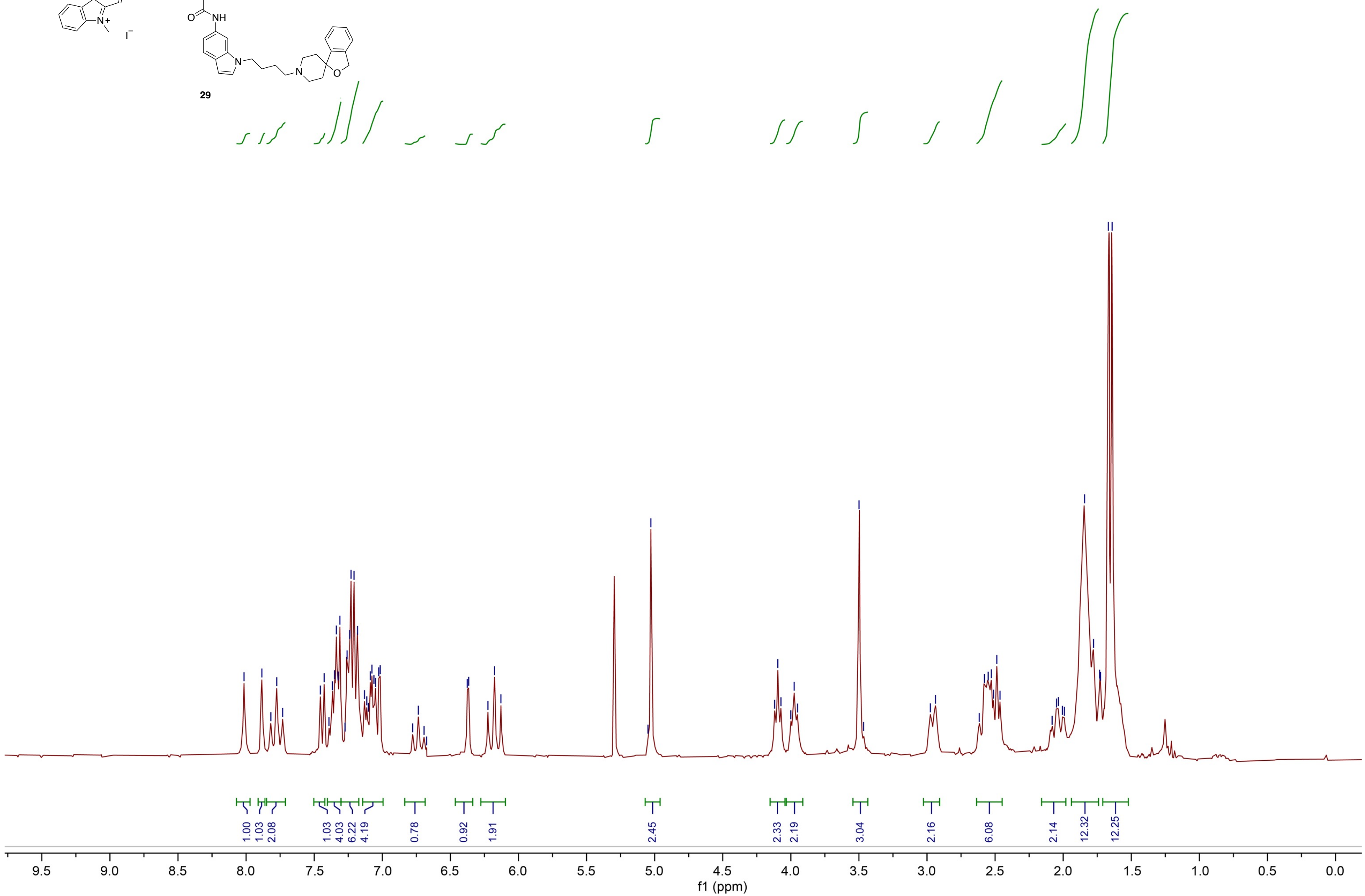

1.00
1.03
2.08
1.03
4.03
6.22
4.19
0.78
0.92
1.91
2.45
2.33
2.19
3.04
2.16
6.08
2.14
12.32
12.25
9.5
9.0
8.5
8.0
7.5
7.0
6.5
6.0
5.5
5.0
4.5
4.0
3.5
3.0
2.5
2.0
1.5
1.0
0.5
0.0
f1 (ppm)

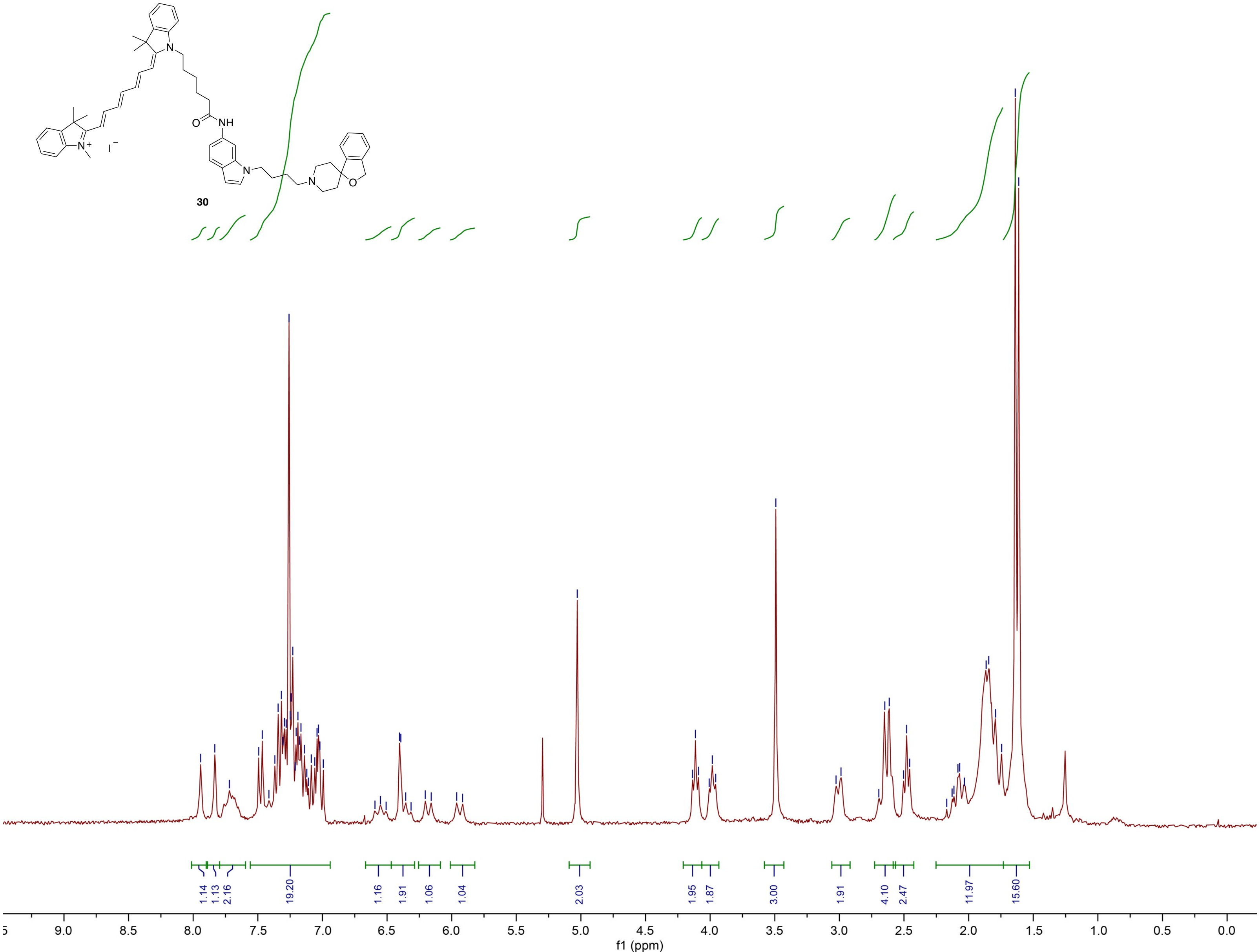

N
N+
I−
O
NH
N
N
O
30
1.14
1.13
2.16
19.20
1.16
1.91
1.06
1.04
2.03
1.95
1.87
3.00
1.91
4.10
2.47
11.97
15.60
9.0
8.5
8.0
7.5
7.0
6.5
6.0
5.5
5.0
4.5
4.0
3.5
3.0
2.5
2.0
1.5
1.0
0.5
0.0
f1 (ppm)
